# A worm-like nucleic acid nanostructure for gene delivery and endosomal escape via ClC3 ion exchanger

**DOI:** 10.1126/sciadv.adw0891

**Published:** 2026-03-06

**Authors:** Yu Xiao, Cecilia Ka Wing Chan, Leo Kit Cheung Lee, Moldir Shyngys, Yee Ting Elaine Chiu, Evelyn Y. Xue, Kathy Oi-lan Lui, Ho Yin Edwin Chan, Sharon Shui Yee Leung, Xiaoqiang Yao, Chung Hang Jonathan Choi

**Affiliations:** ^1^ Department of Biomedical Engineering, The Chinese University of Hong Kong, Shatin, New Territories, Hong Kong SAR.; ^2^ Department of Surgery, The Chinese University of Hong Kong, Shatin, New Territories, Hong Kong SAR.; ^3^ Department of Chemical Pathology, The Chinese University of Hong Kong, Shatin, New Territories, Hong Kong SAR.; ^4^ School of Life Sciences, The Chinese University of Hong Kong, Shatin, New Territories, Hong Kong SAR.; ^5^ School of Pharmacy, The Chinese University of Hong Kong, Shatin, New Territories, Hong Kong SAR.; ^6^ School of Biomedical Sciences, The Chinese University of Hong Kong, Shatin, New Territories, Hong Kong SAR.; ^7^ Center for Neuromusculoskeletal Restorative Medicine, Hong Kong Science Park, Shatin, New Territories, Hong Kong SAR.

## Abstract

Nanoparticle-based gene delivery can enable therapeutic applications with lower cytotoxicity than viral vectors, but its efficacy is often hampered by endosomal entrapment. We present a nucleic acid nanotechnology approach to circumvent this delivery bottleneck by adsorbing therapeutic nucleic acids (DNA, small interfering RNA, microRNA, or messenger RNA) to a gold-polydopamine nanoworm template, thereby assembling a three-dimensional worm-like nucleic acid nanostructure. Devoid of cationic groups, lipids, or mechanical stimuli, this nanostructure naturally activates the chloride voltage-gated channel 3 (ClC3) ion exchanger in endosomes given its worm-like shape; in turn, ClC3 mediates endosomal H^+^ and Cl^−^ accumulation and eventual membrane rupture for cytosolic release, contributing to robust endosomal escape with a correlation coefficient <0.2 between the nanostructure and endosomes. We showcase in vitro gene regulation for primary macrophage polarization and mesenchymal stromal cell differentiation, ex vivo programmable mesenchymal stromal cell–based therapy for kidney fibrosis, and in vivo hepatocyte delivery for treating liver injury. Our versatile nucleic acid nanostructure will empower safe and effective gene therapies.

## Teaser

A worm-like nucleic acid nanostructure robustly escapes endosomes by activating the ClC3 ion exchanger to empower gene delivery.

## INTRODUCTION

Intracellular delivery of nucleic acids forms the technological basis of many therapeutic applications. Nanoparticles (NPs) are established gene carriers with low immunogenicity relative to viral vectors (*1*), but their endosomal escape remains inefficient (*2*). Since 2019, there have been only 14 of >38,000 research articles reporting a correlation coefficient <0.2 between the NP-gene complex and endolysosomes (which denotes little spatial overlap; tables S1 and S2). Although the absence of explicit investigation in those studies does not necessarily imply a failure of endosomal release, it is important to understand the bottleneck of endosomal escape via rigorous, quantitative studies, ultimately benefiting the design of more effective gene carriers. The two mainstream gene carriers for endosomal escape are cationic NPs and lipid-based NPs. Cationic NPs (*3*, *4*) escape endosomes via the “proton sponge effect”; the amine groups buffer endosomal pH, trigger endosomal accumulation of H^+^ (and counterbalancing Cl^−^), and contribute to an elevated osmotic pressure, membrane rupture, and cytosolic release (*5*). The positive charge often induces cytotoxicity (*6*, *7*), a weakness albeit addressable by ionizable lipid NPs (LNPs) (*8*). Lipid-based NPs (liposome and LNP) escape endosomes via “membrane destabilization,” where lipids interact with the luminal endosome membrane for rupture (*9*). Yet, lipid screening and engineering can be challenging, and lipids can induce membrane damage and inflammation (*10*).

Nucleic acid nanostructures [e.g., spherical nucleic acid (SNA) (*11*), origami (*12*), tile-based nanostructure (*13*), and nucleic acid nanogel (*14*)] offer a promising alternative for gene delivery and are widely used in preclinical [and, recently, clinical (*15*)] applications. Their strengths include biocompatibility, transfection-free cellular uptake, entry to cells via specific cell-surface receptors with their three-dimensional (3D) nanoarchitecture (*16*), and mitigated cytotoxicity and immunogenicity given their negative surface charge (*17*). However, their endosomal escape is inefficient; unlike cationic and lipid-based NPs, nucleic acid nanostructures lack an intrinsic mechanism for doing so. Instead, they often require the incorporation of cationic groups (*18*), cell-penetrating peptides (*19*), or mechanical stimuli (*20*) that paradoxically undermine the original merits of nucleic acid nanostructures. Innovative nanostructures with an intrinsic mechanism for endosome escape remain to be desired.

We present a worm-like nucleic acid nanostructure for efficient gene delivery and endosome escape. Specifically, we adsorb therapeutic nucleic acids to the surface of a structural template made of a hybrid gold-polydopamine core-shell nanoworm (Au@PDA NW) (*21*), thereby assembling a 3D worm-like nucleic acid nanostructure. The Au cores enable the ultrastructural tracking of endosomal escape by transmission electron microscopy (TEM) and quantification of cellular transport by inductively coupled plasma mass spectrometry (ICP-MS). The polydopamine (PDA) shell preserves the worm structure and enables the adsorption of diverse gene cargoes [DNA, small interfering RNA (siRNA), microRNA (miRNA), and with extra lipids, mRNA]. The overall nanostructure is anionic, enters cells without transfection agents, and escapes endosomes with a Pearson correlation coefficient (PCC) <0.2 in four cell types, all without cationic/lipidic functional groups or mechanical stimuli. We find that this nanostructure naturally activates the chloride voltage-gated channel 3 (ClC3) ion exchanger thanks to its worm-like shape; in turn, ClC3 mediates vesicular accumulation of H^+^ and Cl^−^ and endosomal escape, a unique mechanism for NP-based gene carriers. Our nanostructure enables in vitro miRNA-enabled macrophage polarization and siRNA-enabled stromal cell differentiation, ex vivo mRNA-enabled cell-based therapy for reducing kidney fibrosis, and in vivo mRNA delivery to hepatocytes for treating liver injury, outperforming Lipofectamine (Lipo; a commercial transfection agent) in endosomal escape and efficacy.

## RESULTS

### Preparation of a worm-like nucleic acid nanostructure

**Fig. 1.**
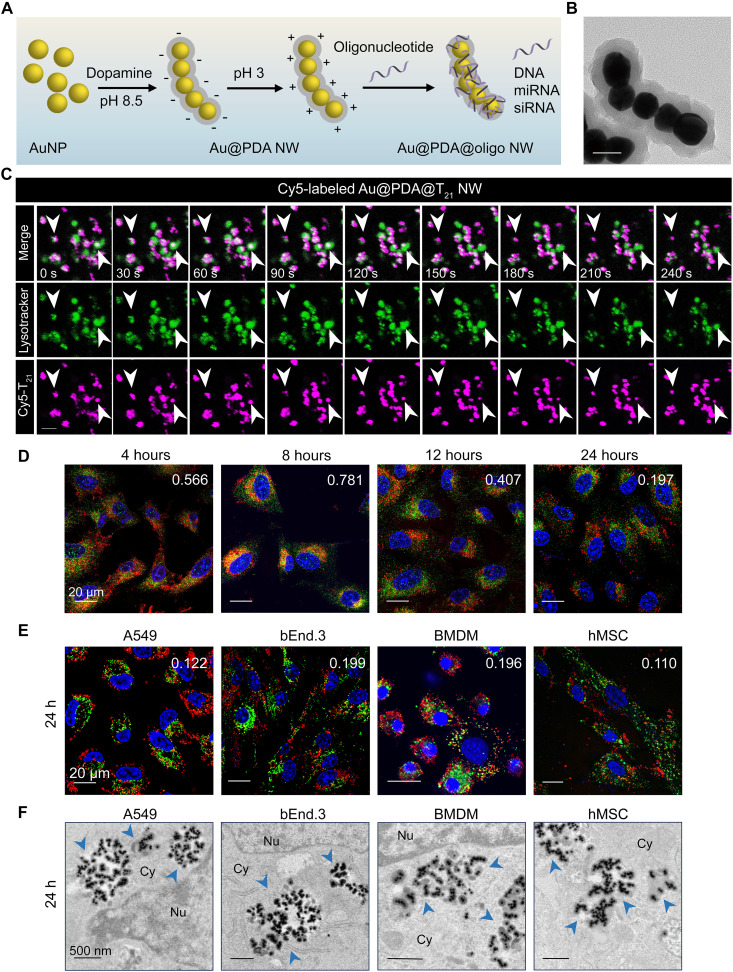
Au@PDA@oligonucleotide NW for intracellular delivery and endosomal escape. (**A**) Preparation route of Au@PDA@oligonucleotide NW for delivering multiple types of oligonucleotides. (**B**) The representative TEM image of T_21_ DNA-adsorbed NWs (Au@PDA@T_21_ NWs) shows the core-shell Au@PDA NW structure. Scale bar, 50 nm. (**C**) Time-lapse confocal images reveal the escape of some Au@PDA@T_21_ NWs (magenta) from acidic vesicles (green; LysoTracker) after 8 hours of incubation. White arrows indicate the onset of endosomal escape, an event marked by the shrinkage or disappearance of the fluorescence of acidic vesicles (green). Scale bar, 2 μm. (**D**) Trafficking of Cy5-labeled Au@PDA@T_21_ NWs (red) in A549 cells as a function of incubation time. Most Au@PDA@T_21_ NWs escape from late endosomes after 8 hours of incubation. Rab9: marker of late endosome. (**E**) Confocal images of four different cell types show limited colocalization of Cy5-labeled Au@PDA@T_21_ NW with acidic vesicles 24 hours postincubation. [(D) and (E)] Blue, nuclei. The white number indicates PCC between Cy5-labeled Au@PDA@T_21_ NW (red) and Rab9 or LysoTracker (green). (**F**) Representative TEM images of four different cell types show cytosolic accumulation of NW (blue arrows) 24 hours postincubation. Nu, nucleus; Cy, cytosol.

Before gene loading, we prepared the worm-shape template core (Au@PDA NW) by sonicating citrate-capped 40-nm Au NPs with monomeric dopamine at alkaline pH for 1 hour (*21*). Previously, we proved that NW formation involves two steps: (i) dopamine-induced 1D assembly of citrate-capped Au NPs to form the Au NW core template and (ii) growth of the outer PDA shell surrounding the NW core template by self-polymerization of dopamine (*21*). The catechol groups of PDA enable π-π stacking with nucleobases, and its hydroxyl and amine groups form hydrogen bonds with the nitrogenous bases and phosphates of oligonucleotides (*22*, *23*). We flipped the surface charge of Au@PDA NW from negative to positive by incubation at pH 3, facilitating the electrostatic interactions between protonated PDA and negatively charged oligonucleotides. In our proof-of-concept studies, we electrostatically adsorbed polythymidine oligonucleotides with 21 thymidines (Ts; T_21_) to construct the worm-like nucleic acid nanostructure (Au@PDA@T_21_ NW) (Fig. 1A). We chose T_21_, a noncoding sequence, to (i) prevent any regulation or expression of proteins that affect cellular trafficking or endosomal escape of the NW and (ii) enhance oligonucleotide loading on the NW as T has the smallest size among the four standard nucleobases (*24*). On average, each Au@PDA@T_21_ NW had four or five Au cores, an overall shell of PDA and T_21_ of ~20 nm in thickness, a length of ~224 nm (Fig. 1B and figs. S1 and S3), a zeta potential of −37.8 mV, and ~1024 DNA strands (1.9 strand/100 nm^2^ of PDA surface); the NW was stable in 10% fetal bovine serum (FBS) (tables S3 and S4) and not toxic on A549 lung epithelial cells, bEnd.3 brain endothelial cells, mouse bone marrow–derived macrophages (BMDMs), or human mesenchymal stromal cells (hMSCs) 24 hours postincubation (fig. S4). Treatment with amiloride severely reduced the association of Au@PDA@T_21_ NWs to all four cell types, indicating macropinocytosis as a major uptake pathway like PDA-coated NPs (*25*). Treatment with fucoidan attenuated cellular association, suggesting a minor role of scavenger receptor in uptake like SNAs (figs. S5 and S6) (*16*).

### Endosomal escape of oligonucleotide-adsorbed NWs

We treated A549 cells with Cyanine 5 (Cy5)–labeled Au@PDA@T_21_ NWs and monitored their cellular trafficking. Time-lapse confocal imaging revealed the onset of NW escape from acidic vesicles after 8 hours of incubation (Fig. 1C). By labeling different vesicles using immunofluorescence, we detected that the NWs abundantly entered cells by 4 hours postincubation and became strongly localized to late endosomes (PCC = 0.781) 8 hours postincubation but no longer overlapped with late endosomes 12 hours (PCC = 0.407) or 24 hours (PCC = 0.197) postincubation (Fig. 1D). They never strongly resided in early endosomes (PCC ~ 0.2) or lysosomes (PCC ~ 0.25) (fig. S7). So, most NWs escaped from late endosomes before reaching lysosomes. For the other three cell types tested, Au@PDA@T_21_ NWs also showed limited overlap with acidic vesicles (PCC ~ 0.1 to 0.2) (Fig. 1E). These PCC values were consistently lower than those obtained by using conventional carriers to deliver the same amounts of T_21_ [Lipo, polyethylenimine (PEI), and ionizable LNP] to the four cell types (fig. S8). Lipo, PEI, and LNP reduced cell viability to different extents (fig. S9). TEM images of the four cell types confirmed cytosolic accumulation of NW (Fig. 1F and fig. S10). Furthermore, changing the sequence from T_21_ to A_21_ (polyadenosine with 21 As, also noncoding) on the NW did not affect endosomal escape; Au@PDA@A_21_ NW escaped endosomes in the four cell types (PCC < 0.2) (fig. S11). This result agrees with the endosomal escape of NWs that were loaded with other types of gene cargoes (miRNA, siRNA, and mRNA; vide infra). Thus, endosomal escape of NW should not be sequence-dependent.

We prepared a control NP called “Au@PDA@T_21_ NP” by adsorbing a similar loading density of T_21_ (1.8 strand/100 nm^2^) to an unassembled Au@PDA NP with the same Au core size and PDA shell thickness as Au@PDA@T_21_ NW (fig. S1B). Au@PDA@T_21_ NPs were mostly inside acidic vesicles 24 hours postincubation (PCC = 0.755; fig. S12), matching our reports on PDA NPs (*25*) and Au-cored SNA (*26*). Furthermore, a Au@PDA@T_21_ nanorod (NR) of a similar dimension, PDA thickness, and aspect ratio to Au@PDA@T_21_ NW barely localized with acidic vesicles (PCC = 0.137), but it entered cells 65% less abundantly than NW (figs. S2 and S13). Overall, an aspect ratio of 3 to 4 and a total length of 200 to 240 nm are suitable parameters for endosomal escape.

### Role of *ClC3* in endosomal escape

**Fig. 2.**
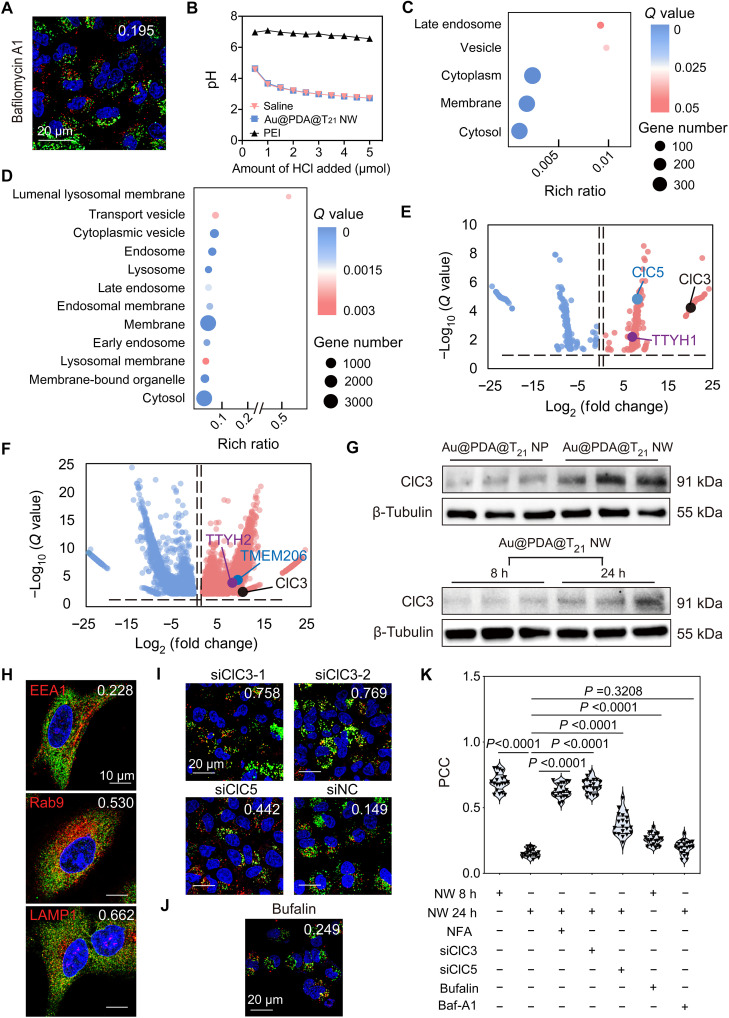
*ClC3* mediates the endosomal escape of Au@PDA@T_21_ NW in A549 cells. (**A**) Inhibition of V-ATPase by bafilomycin A1 did not affect the endosomal escape of Au@PDA@T_21_ NW. (**B**) pH titration curves of Au@PDA@T_21_ NW. Saline and branched PEI are controls. (**C** and **D**) Key enriched biological processes (*Q* < 0.05) by comparing (C) “Au@PDA@T_21_ NW 24 hours” to “Au@PDA@T_21_ NP 24 hours” groups and (D) “Au@PDA@T_21_ NW 24 hours” to “Au@PDA@T_21_ NW 8 hours” groups. (**E** and **F**) The volcano plot shows DETs by comparing (E) “Au@PDA@T_21_ NW 24 hours” to “Au@PDA@T_21_ NP 24 hours” groups and (F) “Au@PDA@T_21_ NW 24 hours” to “Au@PDA@T_21_ NW 8 hours” groups. (**G**) Western blotting revealed upregulation of ClC3 in the “Au@PDA@T_21_ NW 24 hours” group over “Au@PDA@T_21_ NP 24 hours” (top row) and the “Au@PDA@T_21_ NW 8 hours” (bottom row) groups. (**H**) Subcellular location of ClC3 ion exchanger (green). EEA1, Rab9, and LAMP1 (red) are markers of early endosome, late endosome, and lysosome, respectively. (**I**) RNA interference–mediated knockdown of *ClC3* disrupted the escape of Cy5-labeled Au@PDA@T_21_ NW (red) from acidic vesicles (green). Knockdown of *ClC5* showed minor disruption. siNC (scrambled siRNA sequence) is a negative control. (**J**) Activation of *ClC3* by bufalin accelerates the endosomal escape of Au@PDA@T_21_ NW 8 hours postincubation. [(A) and (H) to (J)] The white number indicates PCC between Cy5-labeled Au@PDA@T_21_ NW (red) and LysoTracker (green). Blue, nuclei. [(C) to (G)] *n* = 3 biological replicates for all groups across one experiment. (**K**) Summary of PCC values under different treatments. “NW” represents “Au@PDA@T_21_ NW.” Data are presented as the means ± SEM. Statistical significance was calculated by unpaired Student’s *t* test. ns, not significant (*P* > 0.05). *n* = 20 frames per group (~200 cells were calculated, with 1 mean value calculated per frame).

We validated that the proton sponge effect did not contribute to NW endosomal escape. Pretreatment of A549 cells by bafilomycin A1 (an inhibitor of endosomal acidification) did not impair endosomal escape (Fig. 2A). The NW retained a similar negative charge (fig. S14) and did not buffer pH between 4.5 (lysosome) and 7.5 (extracellular space) (Fig. 2B). Next, we considered the roles of other ion channels. Pretreatment with niflumic acid (a blocker of Cl^−^ channel) disrupted NW endosomal escape, but pretreatment with lidocaine (a blocker of Na^+^ channels), amiodarone (a blocker of K^+^ channel), or nifedipine (a blocker of Ca^2+^ channel) did not (fig. S15). Therefore, we hypothesized that the Cl^−^ channel contributes to endosomal escape.

We used unbiased RNA sequencing to analyze changes in gene expression upon incubating A549 cells with Au@PDA@T_21_ NW. On the effect of nanostructure shape, a pairwise comparison of the gene changes upon treatment with Au@PDA@T_21_ NW versus Au@PDA@T_21_ NP for 24 hours revealed enriched gene ontology (GO) terms related to late endosome, vesicle, and membrane (Fig. 2C and table S5). On the effect of incubation time, a pairwise comparison of the gene changes upon incubation of Au@PDA@T_21_ NW for 24 hours (when NWs were in the cytosol) versus 8 hours (when NWs were in late endosomes) revealed enriched GO terms related to transport vesicle, late endosome, and endosome membrane (Fig. 2D and table S6). Next, we screened for mRNA differentially expressed transcripts (DETs) related to Cl^−^ with a log_2_ fold change >2 or <−2 and a statistical significance of *Q* < 0.05. Notably, we detected marked upregulation of *ClC3* (a 2Cl^−^/H^+^ exchanger) in both pairwise comparisons (Fig. 2, E and F), with log_2_ fold changes of 20.12 for “Au@PDA@T_21_ NW 24 hours versus Au@PDA@T_21_ NP 24 hours” (*Q* = 5.69 × 10^−5^) and 9.93 for “Au@PDA@T_21_ NW 24 hours versus Au@PDA@T_21_ NW 8 hours” (*Q* = 0.029). In mammals, there are nine ClC isoforms: ClC1, ClC2, ClC-Ka, and ClC-Kb are plasma membrane Cl^−^ channels, and ClC3 through ClC7 are 2Cl^−^/H^+^ exchangers in the endosome or lysosome for regulating luminal acidification and Cl^−^ accumulation (*27*). DET analysis also revealed the upregulation of *ClC5*, *TTYH1* (tweety family member 1) and *TTYH2* (both Cl^−^ channels), and *TMEM206* (a proton-activated Cl^−^ channel), albeit less pronounced than *ClC3*. Western blotting revealed higher ClC3 expression in A549 cells in the “Au@PDA@T_21_ NW 24 hours” group than “Au@PDA@T_21_ NP 24 hours” or “Au@PDA@T_21_ NW 8 hours” groups (Fig. 2G and fig. S16). Immunofluorescence verified ClC3 expression in lysosomes (PCC = 0.662) and, moderately, late endosomes (PCC = 0.530) (Fig. 2H and fig. S17). The activation of *ClC3* is not observed in the conventional gene carriers (i.e., Lipo, PEI, or LNP) (fig. S18). Thus, we hypothesized that *ClC3* mediates endosomal escape.

Genetic knockdown using two different siRNA sequences against *ClC3* (fig. S19) increased NW accumulation in acidic vesicles of A549 cells 24 hours postincubation (PCC > 0.75), but treatment with a nonspecific control siRNA sequence did not (Fig. 2I). Activation of *ClC3* using bufalin (*28*) [as verified by quantitative reverse transcription polymerase chain reaction (qRT-PCR); fig. S20] yielded limited overlap of NWs with acidic vesicles 8 hours postincubation (PCC = 0.249; Fig. 2J), while NWs were entrapped in late endosomes without bufalin treatment (Fig. 1D). Our data underscore a pivotal role of *ClC3* in endosomal escape (Fig. 2K). siRNA knockdown of *ClC5* yielded moderate accumulation in acidic vesicles (PCC = 0.442; Fig. 2I and fig. S21), suggesting a minor role of *ClC5*.

### Ionic accumulation and endosomal membrane rupture

**Fig. 3.**
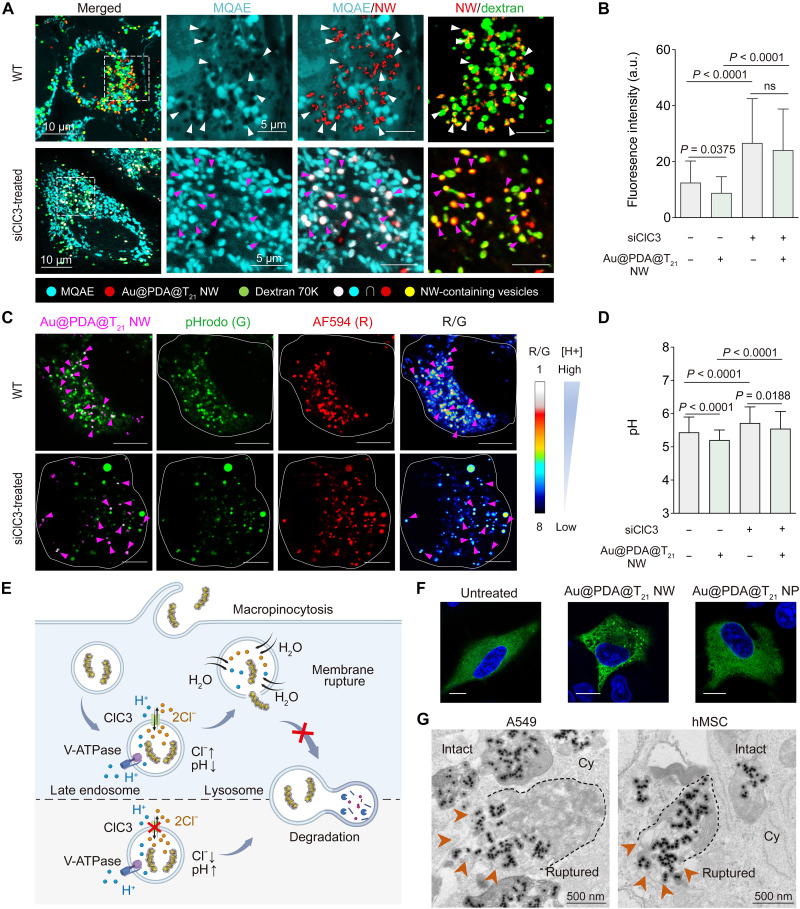
Ion concentration and membrane rupture of endosomes in A549 cells. (**A**) Cl^−^ concentration of intracellular vesicles containing Cy5-labeled Au@PDA@T_21_ NWs (red) in cells without (upper row) or with siClC3 treatment (lower row) 8 hours postincubation. TRITC-conjugated dextran 70K (green) is a tracer of macropinocytosis. The overlapping fluorescence (yellow) of dextran and NW indicates NW-containing vesicles. NW-containing vesicles in wild-type cells have a higher Cl^−^ concentration (quenched MQAE fluorescence; white arrows) than siClC3-treated cells (strong MQAE fluorescence and white color yielded by colocalization of MQAE and Cy5 fluorescence; magenta arrows). (**B**) Quantification of MQAE fluorescence per vesicle. a.u., arbitrary units. (**C**) pH of intracellular vesicles without (upper row) or with siClC3 treatment (lower row) 8 hours postincubation with Cy5-labeled Au@PDA@T_21_ NWs (magenta), dextran 10K pHrodo (green), and dextran 10K AF594 (red). The H^+^ concentration in NW-containing vesicles (white arrows) is calculated by the R/G intensity ratio (inversely proportional to the H^+^ concentration). Scale bars, 10 μm. (**D**) Quantification of the pH of individual vesicles in wild-type and siClC3-treated cells on the basis of R/G ratio. [(B) and (D)] *n* = 300 intracellular vesicles per group. Data are presented as the means ± SEM. Statistical significance was calculated by a one-way ANOVA with Tukey’s test for post hoc analysis. ns, not significant (*P* > 0.05). (**E**) Proposed mechanism for the endosomal escape of Au@PDA@T_21_ NW. Created in BioRender. C. H. J. Choi (2025); https://biorender.com/t4d0rf1. (**F**) Endosomal membrane rupture induced by Au@PDA@T_21_ NW. Green clusters indicate the recruitment of Gal8-GFP to the ruptured vesicle membrane 24 hours postincubation; similar clusters were undetectable in the Au@PDA@T_21_ NP and untreated groups. Blue, nuclei. Scale bar, 10 μm. (**G**) TEM images show the escape of Au@PDA@T_21_ NW (orange arrow) from the ruptured membrane of intracellular vesicles in A549 cells and hMSCs; the dotted line denotes the intact portion of the membrane after rupture.

There is no commercially available dye to specifically stain Cl^−^ in endosomes (*29*), nor is it feasible to conjugate dyes to Au@PDA NW as PDA quenches fluorescence (*30*). To monitor the vesicular concentration of Cl^−^, we incubated wild-type A549 cells with Cy5-labeled Au@PDA@T_21_ NW and tetramethylrhodamine (TRITC)–labeled dextran, a tracer NP that enters cells via micropinocytosis (the same pathway as NW) (*25*) and primarily stays in endosomes (*31*); this design enabled the tracking of NWs strictly in endosomes 8 hours postincubation. Confocal imaging revealed punctate dots with overlapping Cy5 and TRITC fluorescence, indicating NW-containing endosomes. Next, we added a fluorescent Cl^−^ indicator called *N*-(ethoxycarbonylmethyl)-6-methoxyquinolinium bromide (MQAE) to stain intracellular Cl^−^; MQAE fluorescence drops with increasing Cl^−^ concentration. Notably, we detected quenched MQAE fluorescence in NW-containing vesicles relative to NW-free regions of the same cell (Fig. 3A, upper row), suggesting Cl^−^ hotspots in NW-containing vesicles. Control cells treated with dextran and MQAE only did not cause substantial quenched MQAE fluorescence, verifying that dextran did not affect the Cl^−^ concentration (fig. S22). Last, control *ClC3*-silenced A549 cells showed little MQAE fluorescence quenching by Cl^−^ in NW-containing and NW-free vesicles (Fig. 3, A and B), indicating that *ClC3* mediates vesicular Cl^−^ accumulation upon NW treatment.

There are commercially available dye-dextran conjugates to specifically stain H^+^ in acidic vesicles and quantify vesicular pH by a ratiometric method (*32*). Here, we incubated wild-type A549 cells with Cy5-labeled Au@PDA NW and two ratiometric dyes, Alexa Fluor 594–conjugated dextran (a pH-insensitive dye; red) and pHrodo green–conjugated dextran (a pH-sensitive dye; green) for 8 hours. Colocalization of the three fluorescence signals enables the quantification of H^+^ concentration in NW-containing vesicles on the basis of the red-to-green (R/G) intensity ratio (fig. S23); the R/G ratio is inversely proportional to the H^+^ concentration. The mean pH of NW-containing vesicles was 5.20. As control, cells treated with dye-dextran conjugates only exhibited a mean vesicular pH of 5.44, implying that NW treatment led to vesicle acidification. For *ClC3*-silenced A549 cells, vesicular mean pH values were 5.55 and 5.70 with and without NW treatment, respectively (Fig. 3, C and D), less acidic than wild-type cells. Therefore, ClC3 mediates vesicular H^+^ accumulation upon NW treatment, matching past reports on *ClC3* overexpression leading to endosomal H^+^ and Cl^−^ accumulation (*33*, *34*). Such NW-induced vesicular acidification prompted us to assess the activity of vacuolar-type adenosine triphosphatase (V-ATPase), because NW treatment did not affect its expression by RNA sequencing (fig. S24). Confocal images portrayed enhanced colocalization of V0 and V1 (major subunits of V-ATPase) upon NW treatment for 8 hours (fig. S25), indicating higher V-ATPase activity (*35*). We reasoned that the activated V-ATPase pumped more H^+^ to the endosome to (i) balance the influx of Cl^−^ (because of ClC3 activation) and (ii) compensate for the efflux of H^+^ from the endosome via ClC3 (a 2Cl^−^/H^+^ ion exchanger), yielding a net accumulation of H^+^ in vesicles and a more acidic pH. Overall, the NW trafficked to late endosomes, where they upregulated *ClC3*, caused the accumulation of Cl^−^ and H^+^, membrane rupture, and endosomal escape (Fig. 3E).

To verify this claim, we prepared A549 cells that express galectin-8 (Gal8) fused with the green fluorescent protein (GFP). Gal8-GFP is a cytosolic protein that will be recruited to the inner ruptured endosomal membrane, yielding strong GFP fluorescence (*36*, *37*). Treatment of Gal8-GFP–expressing cells with Au@PDA@T_21_ NW yielded punctate spots (~15 dots per cell), proof of endosomal rupture, but there were no obvious Gal8 spots in untreated or Au@PDA@T_21_ NP–treated cells (Fig. 3F and fig. S26). TEM imaging of A549 cells also captured the impaired endosomal membrane and NW escape to the cytosol, with rupture sites on membrane segments near NWs. To demonstrate the versatility of endosomal membrane rupture induced by Au@PDA@T_21_ NW, we further repeated the TEM imaging experiment on another cell type, i.e., hMSCs, and observed similar results (Fig. 3G and fig. S27). As excessive endosomal membrane rupture caused inflammation (*10*), we verified that NW did not trigger a strong inflammatory response (fig. S28).

### In vitro delivery of diverse oligonucleotides to various cell types

**Fig. 4.**
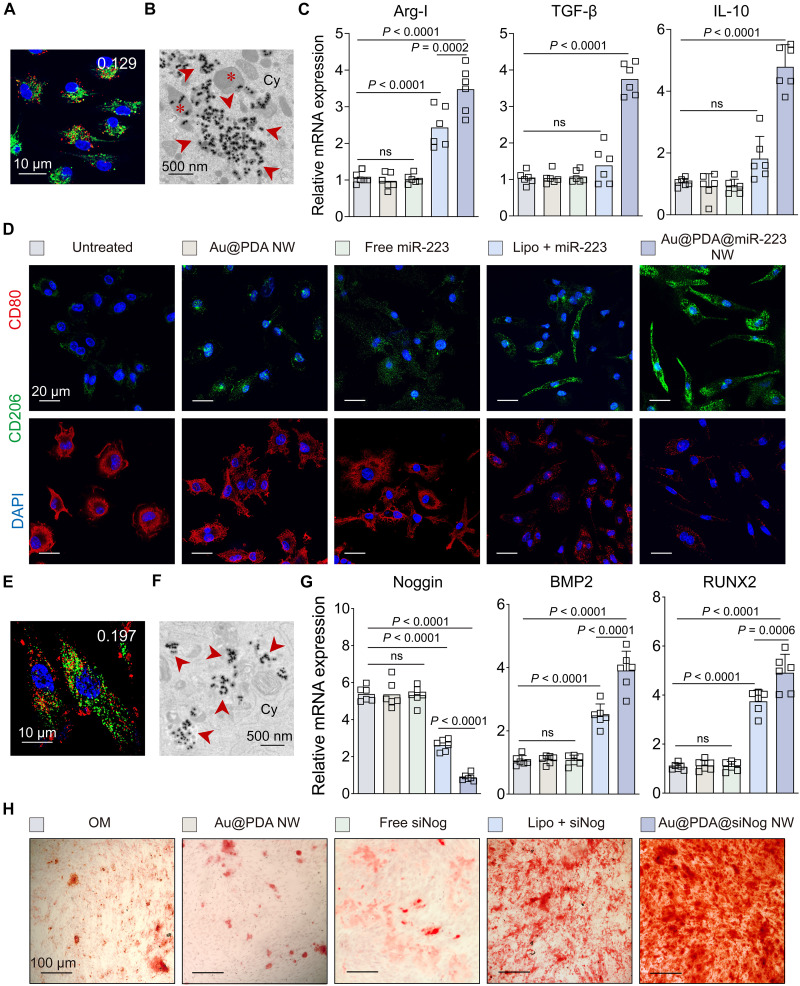
In vitro NW-enabled transfection of oligonucleotides for inducing the polarization of BMDMs and osteogenic differentiation of hMSCs. (**A**) Cy5-labeled Au@PDA@miR-223 NWs (red) did not accumulate in acidic organelles (green) in BMDMs 24 hours postincubation. The white number indicates PCC between Cy5-labeled NW (red) and LysoTracker (green). (**B**) The representative TEM image shows the endosomal escape of Au@PDA@miR-223 NWs in BMDMs 24 hours postincubation. Red asterisks denote vesicles, and red arrows denote free Au@PDA@miR-223 NWs in the cytosol. (**C**) qRT-PCR measurements of M2 phenotype markers in BMDMs. (**D**) Confocal images show the repolarization of BMDMs from M1 to M2, as evidenced by more intense fluorescence of M2 markers (CD206; green) and weaker M1 markers (CD80; red). (**E**) Cy5-labeled Au@PDA@siNog NW did not accumulate in acidic organelles (green) 24 hours postincubation in hMSCs. The white number indicates PCC between Cy5-labeled NW (red) and LysoTracker (green). [(A), (D), and (E)] Blue, nuclei. (**F**) The representative TEM image shows the endosomal escape of Cy5-labeled Au@PDA@siNog NWs in hMSCs 24 hours postincubation. Red arrows denote free Au@PDA@siNog NWs in the cytosol. (**G**) qRT-PCR measurements of markers of osteogenesis in hMSCs 14 days postinduction of differentiation. (**H**) Calcium deposit of hMSCs 14 days postosteogenic differentiation stained by Alizarin Red. *n* = 3 biological replicates per group across one experiment. [(C) and (G)] Data are presented as the means ± SEM. Statistical significance was calculated by a one-way ANOVA with Tukey’s test for post hoc analysis. ns, not significant (*P* > 0.05). *n* = 6 biological replicates per group across two experiments.

We used Au@PDA NWs to deliver antisense DNA, miRNA, or siRNA to different cell types. Using the same method of adsorbing T_21_ to Au@PDA NW, these oligonucleotide-adsorbed NWs had a similar size, surface charge, and DNA/RNA loading to Au@PDA@T_21_ NW; were stable in FBS (tables S3 and S4); entered cells without transfection agents; were not cytotoxic (fig. S4); and escaped endosomes 24 hours postincubation (Fig. 4, A, B, E, and F, and figs. S29 to S31). For all three applications below, we added identical amounts of oligonucleotides and NWs to cells across all groups.

Our first application was antisense gene regulation, where we delivered antisense DNA against enhanced GFP (asEGFP) to EGFP-expressing bEnd.3 cells. Treatments were free asEGFP, Au@PDA@asEGFP NW, and a mixture of Lipo and asEGFP (Lipo + asEGFP). By flow cytometry analysis, Au@PDA@asEGFP NW and Lipo reduced the number of EGFP-positive cells by 61.7 and 24.3% relative to untreated cells 72 hours postincubation, respectively, but there was no effect for free asEGFP (fig. S32).

Our second application was macrophage repolarization by delivering *miR-223* (an M2 macrophage stimulant) (*38*) to M1 BMDMs. Treatments were free *miR-223*, Au@PDA NW, Au@PDA@*miR-223* NW, and Lipo + *miR-223* for 48 hours. Au@PDA@*miR-223* NW activated M2-related genes [interleukin-10 (*IL-10*), *CD206*, transforming growth factor–β (*TGF-*β), and arginase-I (*Arg-I*)] and inhibited M1-related genes [*IL-12*, *CD80*, *TNF-*α, and interferon-γ (*IFN-*γ)] most effectively (Fig. 4C and fig. S33). Au@PDA@*miR-223* NW yielded intense signals of *CD206* and weak signals of *CD80*, yet Au@PDA NW or free *miR-223* showed opposite trends. Lipo + *miR-233* modestly triggered repolarization, but it was less effective than *miR-223*–adsorbed NW (Fig. 4D).

Our third application was hMSC differentiation, where we delivered siRNA against *Noggin* (a negative regulator of osteogenic differentiation) (*39*) to hMSCs. The treatments, including free si*Nog*, Au@PDA NW, Au@PDA@si*Nog* NW, and Lipo + si*Nog*, lasted for 14 days. Au@PDA@siNog NW inhibited Noggin and activated osteogenesis-related genes [i.e., bone morphogenetic protein 2 (*BMP2*) and Runt-related transcription factor 2 (*RUNX2*)] most effectively and yielded the most calcium deposits. Lipo + siNog modestly led to hMSC differentiation (Fig. 4, G and H).

### Preparation of mRNA-adsorbed NWs

**Fig. 5.**
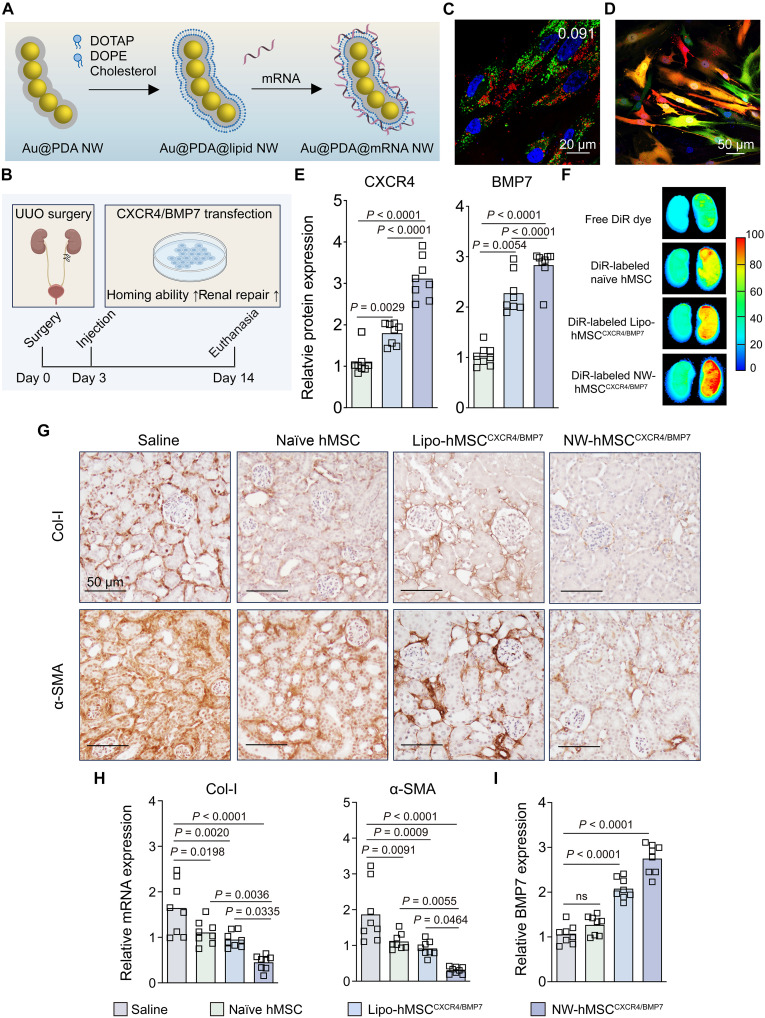
Ex vivo NW-enabled transfection of mRNA in hMSCs as cell-based therapy of renal fibrosis. (**A**) Schematic illustration of the synthesis of mRNA-adsorbed, lipid-coated Au@PDA NWs. (**B**) Disease development and treatment plan. After ligating the left ureter twice with sutures, UUO mice received a single intravenous injection of different treatments on day 3 and were euthanized on day 14 post–UUO surgery. Created in BioRender. C. H. J. Choi (2025); https://biorender.com/t4d0rf1. (**C**) Cy5-labeled Au@PDA@mCXCR4/mBMP7 NWs did not accumulate in acidic organelles 24 hours postincubation. The white number indicates PCC between Cy5-labeled Au@PDA@mCXCR4/mBMP7 NWs (red) and LysoTracker (green). Blue, nuclei. (**D**) The confocal image shows hMSCs that were cotransfected by mRNA expressing CXCR4-GFP (green) and mRNA expressing BMP7-OFP (red) using Au@PDA@lipid NWs 24 hours postincubation. Blue, nuclei. (**E**) Quantification of CXCR4 and BMP7 levels in hMSCs 24 hours posttransfection measured by ELISA. (**F**) Ex vivo fluorescence images of UUO kidneys show the strongest fluorescence for the DiR-labeled Au@PDA@mCXCR4/mBMP7 NW–transfected hMSC (NW-hMSC^CXCR4/BMP7^) group 24 hours postinjection. Lipo-hMSC^CXCR4/BMP7^: Lipo + mCXCR4/mBMP7–transfected hMSC. (**G**) Immunohistochemistry images show the expression of Col-I and α-SMA in the UUO kidney sections. (**H**) mRNA expression of Col-I and α-SMA as quantified by qRT-PCR. (**I**) Quantification of the BMP7 level in the UUO kidney on day 14 by ELISA. [(E), (H), and (I)] Data are presented as the means ± SEM. Statistical significance was calculated by a one-way ANOVA with Tukey’s test for post hoc analysis. *n* = 8 biological replicates per group across two experiments.

We leveraged Au@PDA NW to deliver mRNA. Direct adsorption of mRNA encoding EGFP (mEGFP) to NW did not yield sufficient mRNA loading or protein expression. mRNA has a much higher molecular weight and a more negative charge than the oligonucleotide, so electrostatic repulsion between both the negatively charged mRNA and PDA may become too enormous to be overcome by any mutual attraction because of π-π stacking and hydrogen bonding. Past studies rarely described the direct adsorption of mRNA onto PDA without using a third coating. To circumvent this obstacle, we added small amounts of cationic lipid [1,2-dioleoyl-3-trimethylammonium-propane (DOTAP)], neutral lipid [1,2-distearoyl-*sn*-glycero-3-phosphocholine (DOPE)], and cholesterol to Au@PDA NW as an intermediate coating between mRNA and PDA to reduce their mutual electrostatic repulsion, hence facilitating mRNA loading (Fig. 5A). The Au@PDA@lipid NW had a thicker shell than Au@PDA NW, a hydrodynamic size of 274.5 nm, and a positive charge (+30.5 mV). Upon adsorption with mEGFP, the resultant Au@PDA@mEGFP NW was larger (280.3 nm) and negatively charged (−35.2 mV), had ~68 mEGFP strands (figs. S34 to S36 and tables S7 and S8), and escaped from acidic vesicles 10 hours postincubation with hMSCs (fig. S37). Au@PDA@mEGFP NW also enhanced *ClC3* expression, V-ATPase activity, and vesicular concentrations of Cl^−^ and H^+^ (figs. S38 to S40). Au@PDA@mEGFP NW yielded higher transfection efficiency and cell viability than Lipo (fig. S41). Below, we administered the same amounts of mRNA, NW, hMSCs to cells and mice across all groups.

### Ex vivo mRNA delivery to hMSCs for reducing kidney fibrosis

We used NWs to deliver mRNA to hMSCs for cellular programming and then injected the genetically engineered hMSCs into mice with unilateral ureteral obstruction (UUO), an established model of kidney fibrosis, for ex vivo cell-based therapy (Fig. 5B). To the lipid-coated NW, we adsorbed equal amounts of mRNA encoding BMP7 [an antifibrosis protein (*40*)] fused with orange fluorescence protein (OFP) and mRNA encoding C-X-C chemokine receptor type 4 (CXCR4; for enhancing the homing ability to the fibrosis site) (*41*) fused with GFP. The Au@PDA@mCXCR4/mBMP7 NW had similar physicochemical parameters (288.1 nm, −33.7 mV, and 29 mRNA strands) and serum stability to Au@PDA@mEGFP NW (tables S7 and S8), entered hMSCs without toxicity, barely localized with acidic vesicles (PCC = 0.091) (Fig. 5C), and enabled both protein expressions in ~85% of cells (Fig. 5D and fig. S42).

Into UUO mice, we intravenously injected a single dose of saline, naïve hMSCs, hMSCs transfected with Au@PDA@mCXCR4/mBMP7 NW, or hMSCs transfected with Lipo + mCXCR4/mBMP7. Three days post–UUO surgery is required for developing fibrosis in the right UUO kidney; the left contralateral kidney remains healthy (fig. S43). In vitro, Au@PDA@mCXCR4/mBMP7 NW transfected hMSCs more effectively than Lipo (Fig. 5E). Ex vivo fluorescence imaging verified that NW-transfected hMSCs accumulated in the UUO kidney most abundantly 24 hours postinjection, a proof of homing to the fibrosis site (Fig. 5F and fig. S44). ICP-MS measurements of the blood Au contents revealed a circulation half-life of 10 min for NW-transfected hMSCs; the Au cores offer a chemical handle for tracking hMSCs in vivo because the NW did not readily exit hMSCs upon cellular entry in vitro, presumably due to its large size (fig. S45) (*42*). At euthanasia (14 days post–UUO surgery), NW-transfected hMSCs increased BMP7 (our therapeutic gene) and inhibited fibrosis [as evidenced by reduced levels of type I collagen (Col-I) and α-smooth muscle actin (α-SMA)] in the UUO kidney most effectively (Fig. 5, G to I, and fig. S46), all without in vivo toxicity (figs. S47 and S48). The results proved the enhanced efficacy of hMSCs as a result of NW transfection.

### In vivo mRNA delivery to hepatocytes for reducing liver injury

**Fig. 6.**
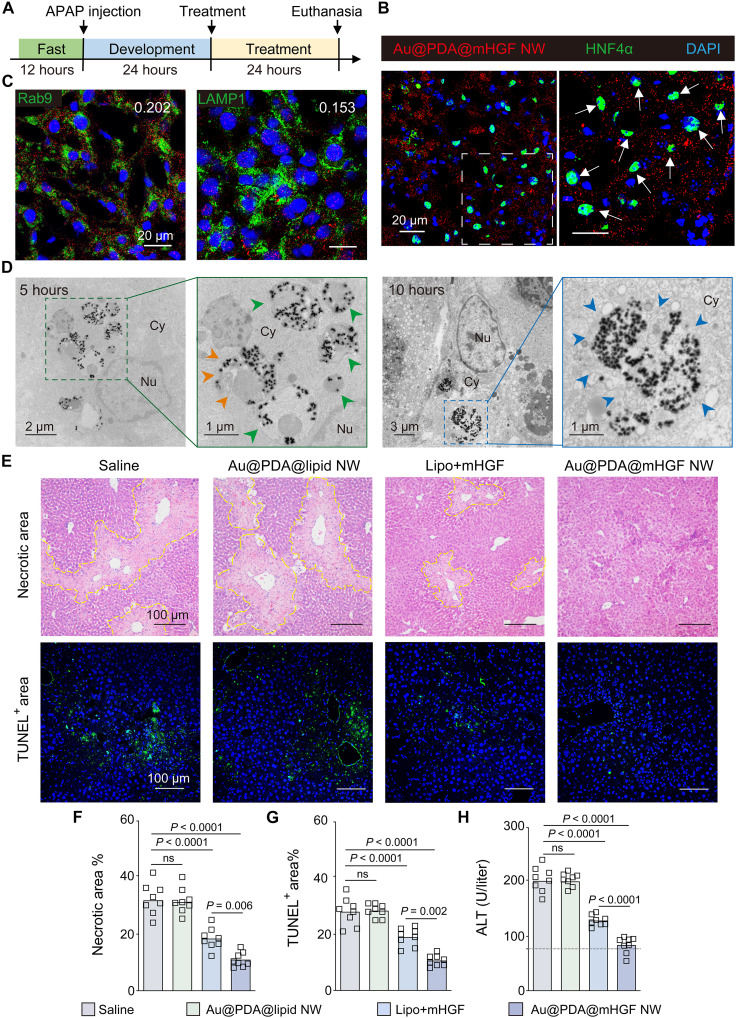
In vivo NW-enabled transfection of mRNA in hepatocytes for reducing ALI. (**A**) Disease development and treatment plan. After intraperitoneally injecting APAP to induce ALI, mice received a single intravenous injection of saline, Au@PDA@lipid NW, Au@PDA@mHGF NW, or Lipo + mHGF on day 1 and were euthanized on day 2. (**B**) Cellular-level distribution of Cy5-labeled Au@PDA@mHGF NW (red) in the liver 24 hours postinjection. Green, hepatocytes (HNF4α). Blue, nuclei. (**C**) Confocal images of the ALI liver reveal limited colocalization of Cy5-labeled Au@PDA@mHGF NWs with acidic vesicles (Rab9: late endosomes; LAMP1: lysosomes) 10 hours postinjection. The white number indicates PCC between Cy5-labeled Au@PDA@mHGF NW (red) and acidic vesicle (green). Blue, nuclei. (**D**) TEM images of the ALI liver show that Au@PDA@mHGF NW accumulated inside intracellular vesicles 5 hours postinjection (green arrow) and in the cytosol 10 hours postinjection (blue arrow). Orange arrows indicate NW escape from the ruptured membrane. (**E** to **H**) Au@PDA@mHGF NW most effectively reduced the necrosis area (dotted yellow line) and apoptotic cells (TUNEL-positive; green) in the ALI liver and the serum ALT level 24 hours posttreatment. Blue, nuclei. The dotted line denotes the upper limit of the normal range. [(F) and (G)] Quantification of necrotic and TUNEL-positive areas based on (E) . Data are presented as the means ± SEM. All statistics are a one-way ANOVA with Tukey’s multiple comparisons test. ns, not significant (*P* > 0.05). *n* = 8 biological replicates per group across two experiments.

We leveraged NW to intravenously deliver mRNA encoding hepatocyte growth factor (mHGF) to hepatocytes for reducing acetaminophen (APAP)–induced acute liver injury (ALI) in mice (Fig. 6A) (*43*). The Au@PDA@mHGF NW, with similar physicochemical properties to Au@PDA@EGFP NW (table S7), had a blood half-life of ~0.65 hours and a liver uptake of ~39% of the injected dose 24 hours postinjection by ICP-MS measurements (fig. S49). By loading Cy5-labeled mHGF to the NW, we showed by confocal imaging their entry to hepatocytes (Fig. 6B) with limited overlap with late endosomes (PCC = 0.202) or lysosomes (PCC = 0.153) (Fig. 6C and fig. S50). TEM imaging of the liver captured NW entrapment in cellular vesicles 5 hours postinjection and cytosolic localization (with ruptured vesicle membranes) 10 hours postinjection (Fig. 6D), a proof of endosomal escape. Notably, qRT-PCR measurements revealed *ClC3* upregulation in the liver only in mice injected with Au@PDA@mHGF NW, not other benchmark gene carriers, such as Lipo + mHGF (which adopts both the proton sponge effect and membrane fusion for endosomal escape), PEI + mHGF (which adopts the proton sponge effect), and conventional Lipo + mHGF (which adopts membrane destabilization but not the proton sponge effect) (figs. S51 and S52). Genetic knockdown of *ClC3* in the ALI liver by RNA interference rendered the endosomal escape of intravenously injected Au@PDA@mHGF NW less efficient, as evidenced by confocal immunofluorescence data that showed an increase in PCC from 0.153 (Fig. 6C) to 0.501 (fig. S53) between the Cy5-labeled mHGF and lysosomes in the liver. These data highlight the role of ClC3 in mediating the endosomal escape of Au@PDA@mHGF NW in vivo.

We intravenously injected a single dose of Au@PDA@mHGF NW, Au@PDA@lipid NW, Lipo + mHGF, or saline into mice 24 hours post–APAP induction (time duration needed for ALI to develop), keeping the Au and mHGF amounts injected constant. Au@PDA@mHGF NW most effectively elevated HGF expression, reduced the necrotic area and apoptotic cells, and restored serum liver function marker levels [alanine aminotransferase (ALT) and aspartate aminotransferase (AST)] to near normalcy 24 hours postinjection (Fig. 6, E to H, and fig. S54), a proof of in vivo NW-aided robust transfection and anti-ALI efficacy. Lipo + mHGF reduced ALI less pronouncedly than Au@PDA@mHGF NW, probably due to less efficient endosomal escape (PCC = 0.423) (fig. S55). We further examined the performance of two other benchmark gene carriers, keeping the same dose of mHGF as Au@PDA@mHGF NW. PEI + mHGF showed limited anti-ALI efficacy, possibly due to its limited delivery to the liver, matching literature precedent (figs. S56 and S57) (*44*). Conventional liposome-mHGF did not yield appreciable efficacy despite its liver accumulation and moderate endosomal escape (PCC = 0.595) (figs. S58 and S59), possibly owing to its less efficient mHGF loading to a neutrally charged liposome [encapsulation efficiency (ee) ~14%] than the cationic Lipo (ee ~ 86.1%). Last, Au@PDA@mHGF NW exhibited no evident toxicity or accumulation in major organs, except the liver and spleen, 8 months postinjection; >60% of NWs were cleared from the liver and spleen (figs. S60 to S64).

## DISCUSSION

This work offers a nucleic acid nanotechnology solution for overcoming the long-standing gene delivery bottleneck of endosomal escape via a 3D, noncationic NW. It is now possible to design an NP-based gene carrier that intrinsically escapes endosomes. Its key advantages are simplicity (requiring no cationic or lipidic groups, cell-penetrating peptides, physical forces, lipid screening, or polymer engineering) and versatility (enabling the loading, cellular delivery, and endosomal escape of diverse gene cargoes in multiple cell types, in vitro and in vivo). With more robust endosomal escape (average PCC: ~0.14) than other NP gene carriers (Lipo, PEI, conventional liposome, and ionizable LNP), we are optimistic that our worm-like nucleic acid nanostructure will enable safer and more effective gene therapy for disease management (e.g., cancer therapy and vaccination). The NW shape may facilitate endosomal escape for several reasons. From the materials perspective, a worm-like NP generally has a larger surface area–to–volume ratio than a spherical NP, possibly enabling closer contact and more prolonged interactions with the endosomal membrane to promote membrane disruption (*45*). Next, the elongated worm-like shape can impart additional curvature stress (*46*) on the endosomal membrane to promote NW escape. From the biology perspective, our large NW (~240 nm long) enters the cell via macropinocytosis, an uptake pathway associated with fluid intake and the formation of larger, less rigid, and more deformable macropinosomes (*47*) that facilitate endosomal escape. Future structural engineering will aim at improving its distribution to organs other than the liver and its biodegradability [as Au NP (*48*) and PDA (*49*) require weeks, if not months, for intracellular degradation]. To improve clinical relevance, switching the Au NP to another more biodegradable NP (e.g., iron oxide) for constructing the NW (*50*) or 1D nanochain core template will support safer and more effective therapies (*51*). We anticipate the birth of next-generation nucleic acid nanostructures that escape endosomes by interacting with various cellular components (e.g., organelles, receptors, and ion channels).

Elucidating how our worm-like nucleic acid nanostructure escapes endosomes has led us to find the role of the ClC3 ion exchanger in endosomal escape. Several Cl^−^ channels and ion exchangers regulate endosomal Cl^−^ concentration (*52*, *53*), with ClC3 regulating ion homeostasis, cellular response, and pathology (*54*). Yet, the specific ion channel or exchanger mediating the endosomal escape of NPs was elusive. Two decades ago, Verkman and colleagues reported that vesicular Cl^−^ accumulation improved gene transfection by polyamine NPs (*55*) and later showed that ClC3 overexpression caused vesicular Cl^−^ and H^+^ accumulation (*34*); collectively, these authors envisioned that ClC3 overexpression enhances gene delivery. Here, we realize this vision using a worm-like nucleic acid nanostructure that naturally triggers ClC3, which mediates vesicular accumulation of H^+^ and Cl^−^ and endosomal escape. While our NW causes vesicular ion accumulation like other gene carriers, it activates ClC3, but Lipo, PEI, LNP, or conventional liposome do not. Researchers can apply this mechanism of ClC3 activation to other NP-based gene carriers or synergize it with other endosome-escaping mechanisms to improve gene therapies.

## MATERIALS AND METHODS

### Synthesis of Au@PDA NWs and Au@PDA NRs

Citrate-capped Au NPs (Cit-Au NPs) of ~40 nm in diameter were synthesized according to an established seed-mediated growth method (*56*). Cit-Au NRs (45 nm by 200 nm) were purchased from Nanopartz. Au@PDA NWs (derived from the assembly of Au NPs) and unassembled Au@PDA NRs were prepared using our published method (*21*). Briefly, 1 ml of 0.1 nM Cit-Au NPs or Cit-Au NRs was diluted by adding 1 ml of tris buffer (10 mM, pH 8.5). Then, 2 ml of freshly prepared dopamine solution (1 mg/ml) in tris buffer was quickly injected into the diluted Au NP or NR solution under sonication. After 60 min, the resultant NWs and NRs were collected and purified by centrifugation at 5000 rpm for 15 min twice. The purified NPs were resuspended in Nanopure water and stored at 4°C.

### Synthesis of unassembled Au@PDA NPs

Briefly, 1 ml of Cit-Au NPs of 40 nm stock solution (0.1 nM) was injected into 1 ml of HS-PEG_5000_-COOH (Jenkem; 0.1 mg/ml) in Nanopure water. After 1 hour of sonication, the resultant Au@PEG NPs (0.1 nM) were collected by centrifugation at 10,000 rpm for 15 min twice. Next, 1 ml of Au@PEG NPs was diluted to 0.05 nM by adding 1 ml of tris buffer (5 mM, pH 8.5), followed by a quick injection of 2 ml of freshly prepared dopamine (0.5 mg/ml) in tris buffer. Upon 1 hour of sonication, the resultant unassembled Au@PDA NPs were collected and purified by centrifugation at 5000 rpm for 15 min twice. The purified Au@PDA NPs were resuspended in Nanopure water and stored at 4°C.

### Synthesis of Au@PDA@oligonucleotide NWs and unassembled Au@PDA@T_21_ NPs and NRs

Concentrated Au@PDA NWs were resuspended in 5 ml of citrate buffer (20 mM, pH 3, in nuclease-free water) to a final concentration of 0.25 nM (by Au core concentration). After 30 min of sonication to tune the negative PDA shell of NW to a positive surface charge, 5 ml of 0.5 μM oligonucleotides [T_21_ or A_21_ DNA, ASO against EGFP (asEGFP), miR-223, or siRNA against Noggin (siNog) (Idobio); in nuclease-free water] was added. After 1 hour of incubation, the resultant Au@PDA@oligonucleotide NWs were collected by centrifugation at 5000 rpm for 15 min at 4°C twice, resuspended in nuclease-free water, and stored at 4°C. Loading T_21_ DNA on unassembled Au@PDA NP and Au@PDA NR followed the same protocol as Au@PDA@T_21_ NW, keeping the identical Au core and T_21_ concentrations. asEGFP: 5′-GAGCTGCACGCTGCCGTC-3′. miR-223: sense strand, 5′-UGUCAGUUUGUCAAAUACCCCA-3′; antisense strand, 5′-GGGUAUUUGACAAACUGACAUU-3′. siNog: sense strand, 5′-AACACUUACACUCGGAAAUGAUGGG-3′; antisense strand, 5′-CCCAUCAUAUUUCGCUGAAGAUUA-3′.

### Characterization of Au@PDA@T_21_ NWs, NPs, and NRs

The concentrations of Au@PDA@T_21_ NW, Au@PDA@T_21_ NP, and Au@PDA@T_21_ NR were determined by ICP-MS (Agilent 7900) with reference to a standard curve of known Au concentration (Au-197 isotope) in parts per billion (ppb). To quantify the Au content in solutions, tissues, and cells, we converted the ICP-MS raw data from ppb to μg/liter using the equation 1 ppb = 1 μg/liter, followed by multiplication of the volume of dilute 2% nitric acid containing the digested samples. The optical spectra of all synthesized nanomaterials were recorded by a Cary 6000i UV-Vis-NIR spectrophotometer (Agilent).

The hydrodynamic diameter and zeta potential measurements were performed at room temperature (RT) using a dynamic light scattering (DLS) analyzer (DelsaMax PRO, Beckman Coulter). The DLS data quality was interpreted by analyzing the autocorrelation function and its fit, where the sum-of-square threshold was set to be under 100. To test colloidal stability, the NWs were dispersed in Dulbecco’s modified Eagle’s medium (DMEM; Gibco, no. 12100046) containing 10% FBS (Gibco, 10270106). After 24 hours of incubation at 37°C, the hydrodynamic diameter and zeta potential were analyzed using DLS. Reported DLS values represent the means ± SD from three independent measurements.

The loading of different types of nucleic acids (DNA, miRNA, siRNA, or mRNA) on the Au@PDA NW, NR, or NP was calculated on the basis of a fluorescence-based assay (*16*). The concentration of Cy5-labeled nucleic acids before and after adsorption onto the Au@PDA NW, NR, or NP was measured by a microplate reader (MULTISKAN GO, Thermo Fisher Scientific), followed by division of the difference in nucleic acid concentration by the concentration of nanomaterial to obtain the loading of nucleic acid. The excitation wavelength for Cy5 is 650 nm, and the emission wavelength is 655 to 750 nm.

### Cell culture

All cells were maintained at 37°C and 5% CO_2_. A549 lung epithelial cells (American Type Culture Collection, no. CCL185) and bEnd.3 brain endothelial cells (American Type Culture Collection, no. CRL2299) were cultured in complete DMEM, supplemented with 10% FBS and 1% penicillin-streptomycin (P/S; Gibco, 15140122). hMSCs (Lonza, no. PT2501) were cultured in α-minimum essential medium (α-MEM; Gibco, no. 11900073) supplemented with 20% FBS, 1% P/S, and 1% l-glutamine (Gibco, no. 25030081). BMDMs were isolated from BALB/c male mice aged 9 to 11 weeks following literature precedent (*57*). Briefly, hind legs were dislocated from the hip bone and sterilized in three changes of 75% ethanol for 1 min. After removing the bones below the knee joint by cutting the ligaments, the separated femurs and tibias were rinsed with precooled phosphate-buffered saline (PBS; Gibco, no. 21600010). The ends of the femur and tibia were cut, and each marrow cavity was irrigated with 5 ml of RPMI 1640 medium (Gibco, no. 11875093) containing 10% FBS. Cells were pelleted by centrifugation at 1500 rpm for 5 min, treated with iced red blood cell lysis buffer (BioLegend, no. 420301) for 2 min, pelleted again by centrifugation at 1500 rpm for 5 min, and cultured in DMEM containing 10% FBS, 1% P/S, and colony-stimulating factor–1 (50 ng/ml; Sino Biological, 51112-MNAH) for 7 days.

### Pharmacological blocking

Cells were plated on 24-well plates and cultured until ~80% confluence was reached. Cells were pretreated with 0.3 ml of complete DMEM containing chemical blockers for 1 hour before incubation with NWs. The choice and working concentration of blockers followed our previous reports (*25*, *58*) as well as other publications. These inhibitors include fucoidan (50 μg/ml; Cayman Chemical) (*16*), methyl-β-cyclodextrin (10 mM; Tokyo Chemical Industry, M1356), filipin III (2.5 μg/ml; MCE, HY-N6718) (*59*), dynasore (120 μM; Cayman Chemical, no. HY-N6718) (*60*, *61*), sodium azide (0.1%; Sigma-Aldrich, S2002) (*62*), and amiloride (0.5 mg/ml; Sigma-Aldrich, BP008) (*63*, *64*). Next, the blocker-containing medium was removed, and 0.3 ml of fresh complete DMEM containing the same blocker at the original concentration and 0.1 nM Au@PDA@T_21_ NWs was added to the cells. After 2 hours of incubation, cells were rinsed with PBS twice and trypsinized (0.25% trypsin-EDTA, Gibco, no. 25200072) for cell counting by a hemocytometer. The cells were further centrifuged at 1500 rpm for 5 min to form a pellet. Cell pellets were digested in 0.25 ml of aqua regia [3:1 (v/v) ratio of 38% HCl (RCI Labscan) and 68% HNO_3_ (VMR Chemical)] overnight and diluted to 5 ml by the matrix solution (2% HCl and 2% HNO_3_ in Nanopure water) for ICP-MS measurements of the Au content associated with the cells.

### Cytotoxicity

Cells were plated on 96-well plates and cultured until ~80% confluence was reached. Then, cells were incubated with Au@PDA@oligonucleotide NWs (0.1 nM) formulated in 0.1 ml of complete culture medium or Au@PDA@mRNA NWs (containing 100 ng of mRNA) in 0.1 ml of complete α-MEM for 24 hours. After two PBS rinses, cell viability was measured by the alamarBlue assay (Invitrogen, DAL1025) according to the manufacturer’s protocol.

### Bio-TEM imaging

Freshly harvested cell pellets or tissue specimens were fixed with glutaraldehyde [2.5% in phosphate buffer; Electron Microscopy Sciences (EMS), no. 16000] for 2 hours and stained with osmium tetroxide (1% in phosphate buffer; EMS, no. 19100) for another 2 hours. Samples were dehydrated in increasing ethanol gradients (30, 50, 70, 80, 90, and 100%) and propylene oxide (Sigma-Aldrich, no. 540048), embedded in Epon 812 resins (EMS, no. 14120), and polymerized at 55°C for 48 hours. Ultrathin sections of ~70 nm in thickness were prepared by using a diamond knife (Ultra 45°, 3.0 mm; DiaTOME) deposited onto 200-mesh copper grids (EMS, G200-Cu) and stained with 4% uranyl acetate (EMS, no. 541-09-3) in 50% methanol/water for 30 min and Reynold’s lead citrate [containing 80 mM lead(II) nitrate (Sigma-Aldrich, no. 10099-74-8) and 120 mM sodium citrate (Sigma-Aldrich, C7254) in Nanopure water] (*65*) for 15 min for observation under a transmission electron microscope at a beam voltage of 100 kV (Hitachi H7700).

### Preparation of T_21_-containing LNP

The LNPs were prepared by Genscript, following a standardized microfluidic mixing protocol on the basis of previously published methods (*66*). Briefly, T_21_-containing LNPs were formulated using a microfluidic chip device by mixing an aqueous phase containing T_21_ with an ethanol phase containing lipids at a 3:1 volume ratio (aqueous:ethanol). The ethanol phase was prepared by dissolving lipids, including ALC-0315 (Broadpharm), cholesterol (Sigma-Aldrich), DMG-PEG2000 (Avanti Research), and DSPC (Broadpharm), at predefined molar ratios of 50:38.5:1.5:10. The aqueous phase consisted of T_21_ dissolved in citrate buffer (pH 4.0). Rapid mixing in the microfluidic device resulted in the formation of LNPs encapsulating T_21_. The LNPs were further purified through overnight dialysis against 1× PBS at 4°C to remove residual ethanol. The resulting LNP formulation was filtered through a 0.2-μm membrane filter to ensure sterility.

### Intracellular trafficking and endosomal escape

Cells were seeded in a 35-mm confocal dish (SPL Life Sciences) and cultured until 80 to 90% confluence was reached. Cells were treated with 1 ml of 0.1 nM Au@PDA@T_21_ NWs (formulated in complete DMEM) for various time durations up to 24 hours. Then, cells were rinsed with PBS twice and stained with 1 ml of 100 nM LysoTracker Green DND-26 (Thermo Fisher Scientific, L7526) formulated in phenol red–free DMEM (Gibco, 31053028) for 30 min. Next, cells were rinsed, stained with 1 ml of Hoechst 33342 (1 μg/ml; Thermo Fisher Scientific, no. 62249) for 10 min, rinsed again, and replenished with 1 ml of phenol red–free complete DMEM for confocal microscopy. The excitation wavelengths for Hoechst 33342, LysoTracker, and Cy5-labeled Au@PDA@T_21_ NWs are 405, 488, and 650 nm, respectively, and their corresponding emission wavelengths are 410 to 480, 495 to 580, and 655 to 750 nm, respectively.

As benchmarks against Au@PDA@T_21_ NW, cells were also treated with different conventional gene transfection agents for delivering T_21_ DNA. (i) For PEI treatment, 10 pmol of Cy5-labeled T_21_ [in 25 μl of Opti-MEM (Gibco, 11058021)] was mixed with 0.5 μl of PEI (1 mg/ml; branched, 25 kDa) (Sigma-Aldrich, no. 408727), vortexed for 10 s, and incubated at RT for 10 min. The resultant mixture was diluted by adding 75 μl of Opti-MEM before addition to the confocal dish. (ii) For Lipofectamine 3000 treatment, 10 pmol of Cy5-labeled T_21_ (diluted in 25 μl of Opti-MEM) was added dropwise to 1 μl of the Lipofectamine 3000 (Invitrogen, L3000015) stock (diluted in 25 μl of Opti-MEM), mixed by gentle pipetting several times, and incubated at RT for 10 min. After adding another 50 μl of Opti-MEM, the resultant mixture was added to the confocal dish. (iii) For LNP treatment, GenScript custom-synthesized LNPs were loaded with Cy5-labeled T_21_. The concentration of the product LNP-T_21_ complex was 0.2 μg/μl by total weight or 30 μM by T_21_ DNA. Accordingly, 0.33 μl of LNP-DNA complex solution (containing 10 pmol of Cy5-labeled T_21_) was diluted by adding 0.1 ml of Opti-MEM by gentle pipetting before addition to the confocal dish. Staining of acidic vesicles by LysoTracker followed the same procedures as above.

### Immunofluorescence of the intracellular fate of NWs

A549 cells were seeded in a 35-mm confocal dish and cultured until 80% confluence was reached. Cells were incubated with 1 ml of complete DMEM containing 0.1 nM Cy5-labeled Au@PDA@T_21_ NWs for various time durations. After two PBS rinses and fixation with 1 ml of methanol for 10 min and 1 ml of acetone for 1 min, cells were blocked by 0.1 ml of 2% bovine serum albumin (BSA) for 1 hour at RT and stained with 0.1 ml of 2% BSA containing primary antibodies against EEA1 (1:100; Abcam, ab2900), Rab9 (1:100 dilution; Abcam, ab179815), or LAMP1(1:150 dilution; Abcam, ab24170) overnight at 4°C. After three PBS rinses, cells were stained with 0.1 ml of 2% BSA containing an Alexa Fluor 488–labeled, goat secondary antibody against rabbit (2 μg/ml; Invitrogen, A-11008) for 1 hour at RT and stained with 0.1 ml of PBS containing 4′,6-diamidino-2-phenylindole (DAPI; 1 μg/ml; Thermo Fisher Scientific, D1306) for 10 min at RT. The excitation wavelengths of DAPI, Alexa Fluor 488, and Cy5 are 405, 488, and 650 nm, respectively. The emission wavelengths of DAPI, Alexa Fluor 488, and Cy5 are 410 to 470, 495 to 600, and 655 to 750 nm, respectively.

### HCl titration

Au@PDA@T_21_ NWs were dispersed in 5 ml of nuclease-free water to a final concentration of 0.1 nM. As a control, 5 ml of saline [0.9% (w/v) of sodium chloride; Sigma-Aldrich, S9888] and 5 ml of branched PEI (1 mg/ml; 25 kDa; Sigma-Aldrich, no. 408727) in Nanopure water were also prepared. After adjusting the pH of these solutions to ~7.5 to mimic physiological pH, titration was initiation by adding 0.1 M HCl dropwise with stirring, and the corresponding pH was measured by a pH meter (Sartorius) until it dropped to 4.5. Reported pH values represent the means ± SD from three independent measurements.

### Influence of V-ATPase and different ion channels on the endosomal escape of NW

A549 cells were plated on 35-mm confocal dishes and cultured until ~80% confluence was reached. Cells were pretreated with 1 ml of complete DMEM containing different blockers for 1 hour, including 200 nM bafilomycin A1 (MCE, HY-100558), 10 mM lidocaine (MCE, HY-B0185), 20 μM amiodarone (MCE, HY-14187), 100 μM nifedipine (MCE, HY-B0284), and 100 μM niflumic acid (MCE, HY-B0493). After removing the blocker-containing medium, 1 ml of fresh complete DMEM containing 0.1 nM Au@PDA@T_21_ NW and different blockers was added to the confocal dish. Twenty-four hours postincubation, cells were stained with LysoTracker by following the same procedures as above.

### Whole-transcriptome analysis with total RNA sequencing

A549 cells with 80 to 90% confluence were seeded in six-well plates in complete DMEM overnight, followed by splitting into three groups (*n* = 3 per group) for studying two pairwise comparisons: (i) shape-dependent effect: incubation with Au@PDA@T_21_ NWs for 24 hours versus incubation with Au@PDA@T_21_ NPs for 24 hours; (ii) time-dependent effect: incubation with Au@PDA@T_21_ NWs for 8 hours versus incubation with Au@PDA@T_21_ NWs for 24 hours. Upon incubation, cells were sent to the Beijing Genomics Institute (BGI) Group for cDNA preparation and whole-transcriptome sequencing. Using a standardized procedure monitored by BGI’s Quality Control System, mRNA was isolated from total RNA using oligo(dT) magnetic beads following the manufacturer’s instructions for cDNA library construction. Double-stranded cDNA was sequenced using the DNBseq platform. At least 20 million clean reads per sample on the DNBseq platform were generated for data analysis. DET detection, GO analysis of DET, and other analyses based on gene expression were performed by BGI. GO terms with corrected *P* values (*Q* values) <0.05 were considered notably enriched among the DETs.

### Immunoblotting

For cell samples, A549 cells, seeded on a six-well plate, were lysed with 1 ml of ice-cold radioimmunoprecipitation assay buffer (Thermo Fisher Scientific, no. 89900) with Pierce protease inhibitors (Thermo Fisher Scientific, A32963). For tissue samples, ~20 mg of liver tissues was homogenized in 1 ml of precooled T-PER tissue protein extraction reagent (Thermo Fisher Scientific, no. 78430) containing Pierce protease inhibitors on ice. After incubating on ice for 5 min (for cells) or 30 min (for tissues), the lysates were centrifuged at 15,000 rpm at 4°C to pellet the cell debris, and the supernatant was subject to the bicinchoninic acid assay (Thermo Fisher Scientific, no. 23225) for quantifying the protein concentration. Twenty micrograms of protein was electrophoresed through a 10% TGX (tris-glycine extended) denaturing polyacrylamide gel (Bio-Rad) and transferred to a polyvinylidene difluoride membrane by a Power Blotter Station (Invitrogen). The membranes were then blocked by 3% BSA (Rockland Immunochemicals) in TBST (tris-buffered saline with Tween 20) at RT for 1 hour and incubated with primary antibodies against ClC3 (1:1000 in 3% BSA/TBST; Thermo Fisher Scientific, MA5-27709) and β-tubulin (1:1000 in 3% BSA/TBST; Abcam, ab108342) at 4°C overnight. After three PBS rinses, the blots were incubated with HRP (horseradish peroxidase)–conjugated secondary antibodies (1:1000 anti-rabbit, Bio-Rad, no. 1706515; 1:3000, anti-mouse, Invitrogen, no. 31430) in 5% nonfat milk (Bio-Rad)/TBST for 1 hour at RT. After five washes with TBST, the membranes were treated with Clarity Max ECL Western Blotting Substrate (Bio-Rad, no. 1705062), and the protein bands were visualized by the ChemiDoc Touch imaging system (Bio-Rad).

### Colocalization of ClC3 with intracellular vesicles by immunofluorescence

A549 cells were plated on 35-mm confocal dishes and cultured until ~80% confluence was reached. After fixation and BSA blocking, cells were first stained with 1 ml of 2% BSA containing primary antibodies against ClC3 (1:100) overnight at 4°C. After two PBS rinses, cells were stained with 1 ml of 2% BSA containing a Cy5-labeled goat secondary antibody against mouse (2 μg/ml; Invitrogen, A10524) for 1 hour at RT and rinsed with PBS twice. Then, cells were incubated with primary antibodies against EEA1 (1:100 dilution), Rab9 (1:100 dilution), or LAMP1 (1:150 dilution) in 2% BSA overnight at 4°C. After rinses, cells were stained with 1 ml of 2% BSA containing an Alexa Fluor 488–labeled, goat secondary antibody against rabbit (2 μg/ml) for 1 hour at RT, stained with 1 ml of PBS containing DAPI (1 μg/ml) for 10 min at RT, and rinsed with PBS twice. The excitation wavelengths of DAPI, Alexa Fluor 488, and Cy5 are 405, 488, and 650 nm, respectively. The emission wavelengths of DAPI, Alexa Fluor 488, and Cy5 are 410 to 470, 495 to 600, and 655 to 750 nm, respectively.

### siRNA transfection

A549 cells were plated on 24-well plates or 35-mm confocal dishes and cultured until ~80% confluence was reached. The transfection solution, formulated in 50 μl of Opti-MEM, contains 1.2 μl of Lipofectamine 3000 and 40 pmol of siRNA that specifically targets human ClC3 (Dharmacon SMARTpool; Santa Cruz, sc-390010), human ClC5 (Santa Cruz, sc-42385), or nontargeting siRNA control (Idobio). After incubation for 10 min and further dilution by adding 350 μl of complete DMEM, 0.4 ml of transfection mixture was added to each well of a 24-well plate. Cells were incubated for 48 hours before harvesting the RNA for qRT-PCR analysis or confocal imaging.

### Staining of intracellular Cl^−^ by MQAE

A549 cells were plated on 35-mm confocal dishes and cultured until ~80% confluence was reached. After two PBS rinses, cells were incubated with 1 ml of Opti-MEM containing 10 mM MQAE (Invitrogen, E3101) at 37°C for 30 min. After removing the staining solution and two PBS rinses, cells were replenished with 1 ml of phenol red–free DMEM for confocal imaging. The excitation wavelength of MQAE is 405 nm, and the emission wavelength range is 410 to 480 nm.

### Ratiometric imaging for intracellular pH measurement

A549 cells were plated on 35-mm confocal dishes and cultured until ~80% confluence was reached. Cells were co-incubated with 1 ml of Opti-MEM containing Cy5-labeled Au@PDA@T_21_ NW (0.1 nM), dextran (10 kDa)–conjugated pHrodo green (100 μg/ml; Invitrogen, P10361), and dextran (10 kDa)–conjugated Alexa Fluor 594 (100 μg/ml; Invitrogen, D22913) for 8 hours. The pH of intracellular acidic vesicles was analyzed by ratiometric analysis of the fluorescence intensities of pHrodo green and Alexa Fluor 594. To plot the calibration curve, cells were incubated with both dextran dyes at 100 μg/ml formulated in 1 ml of Opti-MEM for 8 hours, fixed with 1 ml of 4% formaldehyde for 10 min at RT, and incubated with 1 ml of calibration buffer (Invitrogen, P35379) at a range of pH values (4.5, 5.5, 6.5, and 7.5) for 2 hours before confocal imaging. Individual integral intensities of both dyes in each acidic vesicle were measured at different pH values in three independent experiments with three replicates per experiment (*n* = 300 vesicles). Then, the R/G fluorescence ratio of Alexa Fluor 594 to pHrodo green was calculated, and the calibration curve was plotted on the basis of pH and R/G ratio. The pH of individual vesicles was determined by measuring the R/G ratio and converting it into pH values using the established calibration curve.

### Measurement of V-ATPase activity

Cells were plated on 35-mm confocal dishes and cultured until ~80% confluence was reached. After fixation and blocking in 2% BSA (Rockland Immunochemicals), cells were first incubated with 1 ml of 2% BSA containing a primary antibody against ATP6V1H (1:100; Santa Cruz, sc-166227) overnight at 4°C. After three PBS rinses, cells were stained with 1 ml of 2% BSA containing an Alexa Fluor 488–labeled, goat secondary antibody against mouse (2 μg/ml; Invitrogen, A11001) for 1 hour at RT. After three PBS rinses, cells were incubated with a primary antibody against ATP6V0A2 (1:100; Abcam, ab96803) in 2% BSA overnight at 4°C. After three PBS rinses, cells were stained with an Alexa Fluor 546–labeled, goat secondary antibody against rabbit (2 μg/ml; Invitrogen, A11035) in 2% BSA for 1 hour at RT. Rinsed cells were stained with 1 ml of PBS containing DAPI (1 μg/ml) for 10 min at RT. The excitation wavelengths of DAPI, Alexa Fluor 488, and Alexa Fluor 546 are 405, 488, and 546 nm, respectively. The emission wavelengths of DAPI, Alexa Fluor 488, and Alexa Fluor 546 are 410 to 470, 495 to 540, and 555 to 650 nm, respectively.

### Gal8 recruitment assay

A549 cells were plated on 35-mm confocal dishes and cultured until ~80% confluence was reached. Next, 500 ng of Gal8-GFP plasmid DNA (pDNA; Addgene, no. 127191) and 1.5 μl of Lipofectamine 3000 were added to 50 μl of Opti-MEM; after 10 min of incubation, the mixture was diluted by adding 100 μl of complete DMEM to form the transfection medium and added to the confocal dish. After incubation with the transfection medium for 4 hours, cells were replenished with 1 ml of fresh DMEM and incubated for another 36 hours. Cells were incubated with 1 ml of complete DMEM containing 0.1 nM Au@PDA@T_21_ NWs or Au@PDA@T_21_ NPs for another 24 hours. Last, cells were fixed with 1 ml of cold methanol for 10 min and stained with 1 ml of DAPI (1 μg/ml) for 10 min at RT for confocal microscopy. The excitation wavelengths for DAPI and Gal8-GFP are 405 and 488 nm, respectively, and the emission wavelengths are 410 to 480 and 495 to 600 nm, respectively.

### BMDM polarization

BMDMs were plated on 35-mm confocal dishes and cultured until ~80% confluence was reached. Day 7 BMDMs were stimulated to the M1 phenotype by adding 1 ml of complete DMEM containing lipopolysaccharides (LPSs; 50 ng/ml; Sigma-Aldrich, L7895) and IFN-γ (25 ng/ml; Sino Biological, 11725-H08H). After 24 hours, the LPS/IFN-γ–containing medium was removed, and cells were rinsed with PBS twice and repolarized to the M2 phenotype by incubating with 1 ml of DMEM (1% FBS) containing 0.1 nM Au@PDA NWs, 0.1 nM Au@PDA@miR-223 NWs, or Lipo + miR-223 for 48 hours. After fixation and BSA blocking, polarized BMDMs were stained with 1 ml of 2% BSA containing primary antibodies against CD206 (1:150; Abcam, ab64693) or CD80 (1:100; Abcam, ab254579) overnight at 4°C. After three PBS rinses, cells were stained with 1 ml of 2% BSA containing an Alexa Fluor 488–labeled, goat secondary antibody against rabbit (1 μg/ml) for 1 hour at RT. Rinsed cells were stained with 1 ml of PBS containing DAPI (1 μg/ml) for 10 min at RT. The excitation wavelengths of DAPI and Alexa Fluor 488 are 405 and 488 nm, respectively. The emission wavelengths of DAPI and Alexa Fluor 488 are 410 to 470 and 495 to 650 nm, respectively.

### hMSC osteogenic differentiation

hMSCs were seeded in a 24-well plate until ~90% confluence was reached. Next, hMSCs were treated with 0.4 ml of osteogenic medium (0.05 mM ascorbic acid, 10 mM glycerophosphate, and 0.1 μM dexamethasone in complete α-MEM) containing 0.1 nM Au@PDA NWs, 0.1 nM Au@PDA@siNog NWs, and Lipo + siNog for 14 days, respectively. The medium was changed every 3 days. After fixation in 95% ethanol at RT for 10 min and three PBS rinses, cells were incubated with 400 μl of 2% Alizarin Red S staining solution (Abcam, ab146374) at 37°C for 45 min, washed three times with Nanopure water, and observed under a light microscope (Nikon Eclipse Ni DS-Ri2) to confirm stem cell differentiation. To quantify calcium deposits, cells were treated with 200 μl of 10% acetic acid (Sigma-Aldrich, 695092) at RT for 30 min with shaking to detach the cell monolayer, transferred to a microcentrifuge tube, heated at 85°C for 10 min, and cooled on ice for 5 min. Upon centrifugation at 10,000 rpm for 15 min and removal of the supernatant, 75 μl of 10% ammonium hydroxide (Sigma-Aldrich, no. 338818) was added to dissolve the pellet. Last, the absorbance of the pellet sample at 405 nm was measured by a microplate reader.

### RNA isolation and qRT-PCR analysis

RNA was isolated using RNAiso Plus (Takara, no. 9108) and reverse transcribed using the RevertAid First Strand cDNA Synthesis Kit (Thermo Fisher Scientific, K1622) to generate cDNA. qRT-PCR was performed on the StepOnePlus Real-Time PCR System (Applied Biosystems) using the TB SYBR Green Premix Ex Taq kit (Takara, RR82WR) following the manufacturer’s instructions. Transcript levels were analyzed using the ΔΔCT method and normalized to the housekeeping gene glyceraldehyde 3-phosphate dehydrogenase (GAPDH). Gene expression levels were quantified using predesigned primers purchased from Shanghai Rui Mian Bio-Tech (see the sequences below).

Name of gene (H, human; M, mouse): forward (5′ → 3′); reverse (3′ → 5′): GAPDH (M): ATGGTGAAGGTCGGTGTGAA; GAGGTCAATGAAGGGGTCGT. GAPDH (H): GTCTCCTCTGACTTCAACAGCG; ACCACCCTGTTGCTGTAGCCAA. IL-12 (M): ACGA-GAGTTGCCTGGCTACTAG; CCTCATAGATGCTACCAAGGCAC. IL-10 (M): CGGGAAGACAATAACTGCACCC; CGGTTAGCAGTATGTTGTCCAGC. CD80 (M): CCTCAAGTTTCCATGTCCAAGGC; GAGGAGAGTTGTAACGGCAAGG. CD206 (M): GTTCA-CCTGGAGTGATGGTTCTC; AGGACATGCCAGGGTCACCTTT. TNF-α (M): GGTGCCTATGTCTCAGCCTCTT; GCCATAGAACTGATGAGAGGGAG. TGF-β (M): TGATACGCCTGAGTGGCTGTCT; CACAAGAGCAGTGAGCGCTGAA. IFN-γ (M): CAGC-AACAGCAAGGCGAAAAAGG; TTTCCGCTTCCTGAGGCTGGAT. Arg1 (M): CATTGGCTTGCGAGACGTAGAC; GCTGAAGGTCTCTTCCATCACC. Noggin (H): GCCAGCACTATCTCCAC-ATCCG; AGCAGCGTCTCGTTCAGATCCT. BMP2 (H): TGTATCGCAGGCACTCAGGTCA; CCACTCGTTTCTGGTAGTTCTTC. RUNX2 (H): CCCAGTATGAGAGTAGGTGTCC; CCACTCGTTTCTGGTAGTTCTTC. ClC3 (H): CTGGCATTCATGTCATTTC; CCTCTTTCCAAAGTATAGCAC. ClC3 (M): GGATTTGCCCTCAGAAGAGACC; GGACTTTCTGCTGGAAGAGATGG. Col-I (M): TGTTCAGCTTTGTGGACCTC; TCAAGCATACCTCGGGTTTC. α-SMA (M): GAGGCACCACTGAACCCTAA; CATCTCCAGAGTCCAGCACA. IFN-γ (H): GAGTGTGGAGACCATCAAGGAAG; TGCTTTGCGTTGGACATTCAAGTC. TNF-α (H): CTCTTCTGCCTGCTGCACTTTG; ATGGGCTACAGGCTTGTCACTC. IL-6 (H): AGACAGCCACTCACCTCTTCAG; TTCTGCCAGTGCCTCTTTGCTG. IL-1α (H): TGTATGTGACTGCCCAAGATGAAG; AGAGGAGGTTGGTCTCACTACC.

### Preparation of mRNA-encased, lipid-coated Au@PDA NWs

#### Step 1: Synthesis of lipid-coated Au@PDA NWs (Au@PDA@lipid NWs)

DOTAP (Cayman, no. 15110), cholesterol (Sigma-Aldrich, C8667), and DOPE (Cayman, no. 15091) were added to 10 ml of chloroform (≥99.8%, Fisher Chemical) at a molar ratio of 35:49:16, keeping the total lipid concentration at 1 mg/ml. Upon rotary evaporation of the lipid mixture at 40°C to remove chloroform, 10 ml of Au@PDA NWs (0.5 mg/ml dispersed in nuclease-free water) was added dropwise to the dried lipid film. After sonicating the mixture for 1 hour, the excessive lipids were removed by centrifugation at 5000 rpm for 15 min three times, and the purified Au@PDA@lipid NWs were dispersed in Nanopure water and stored at 4°C.

#### Step 2: Synthesis of mRNA by in vitro transcription

Circular pDNA of EGFP (Addgene, BPK1098) was diluted to a stock solution (1 μg/μl) in nuclease-free water. After diluting 1 μl of the stock pDNA with 16 μl of nuclease-free water, the pDNA was treated with 1 μl of FastDigest BshTI (Thermo Fisher Scientific, FD1464) and 2 μl of digestion buffer for DNA linearization at 37°C for 1 hour. Similarly, the circular pDNA of pHGF (Sino Biological, HG10463-CH), pBMP7-OFP (Sino Biological, G10083-ACR), or pCXCR4-GFP (Sino Biological, HG11325-ACG) was linearized by FastDigest XbaI (Thermo Fisher Scientific, FD0684) according to the procedures mentioned above.

After column purification by the Monarch PCR &DNA cleanup kit [New England Biolabs (NEB), T1030S], the linearized DNA template underwent in vitro transcription using the T7 HiScribe ARCA mRNA Transcription Kit with tailing (NEB, E2060S) per the manufacturer’s instructions. Upon purification by the Monarch RNA Cleanup kit (NEB, T2040L), the mRNA product was characterized by NanoDrop (Thermo Fisher Scientific) for its concentration and by gel electrophoresis for its molecular weight. The 1.2% native agarose gel, made in diethyl pyrocarbonate (Macklin, D6079)–treated TBE (tris-borate-ethylenediaminetetraacetic acid) buffer, contains SYBR Au nucleic acid gel stain (1:10,000 dilution; Thermo Fisher Scientific, S11494). For each gel lane, 2 μl of single-strand RNA ladder (NEB, N0362S) or mRNA product (200 ng) was mixed with 8 μl of RNA loading dye, heated at 90°C for 2 min, and cooled on ice for 2 min before loading. Upon electrophoresis at 80 mV for 1 hour, the gel was imaged by the ChemiDoc Touch imaging system. The final mRNA product was stored at −80°C. Cy5-labeled mRNA was prepared with the Label IT Cy5 Labeling Kit (Mirus Bio) per the manufacturer’s instructions.

#### Step 3: Preparation of mRNA-encased NWs

Typically, 0.2 ml of mRNA (10 μg/ml) was added to 1 ml of Au@PDA@lipid NW (0.1 mg/ml; in nuclease-free water) at a weight ratio of 1:50 (mRNA to NW). Upon incubation for 30 min, the excess mRNA was removed by centrifugation at 5000 rpm for 15 min at 4°C twice.

### Animals

All procedures followed the guidelines stipulated by the Animal Experimentation Ethics Committee at The Chinese University of Hong Kong (approval numbers 21-092-MIS and 22-358-MIS). Male BALB/c and C57 mice between 9 and 11 weeks of age were randomly assigned to various treatment groups. All mice were housed in a temperature- and humidity-controlled environment with a 12-hour light/dark cycle. For distribution and efficacy studies, the sample size (*N*) indicates biological replicates.

### Mouse model of UUO

Male mice were selected for the UUO model for their higher sensitivity to UUO surgery–induced kidney inflammation, fibrosis, and apoptosis than age-matched females (*67*). Male BALB/c mice aged 9 to 11 weeks were anesthetized by an intraperitoneal injection of 0.2 ml of saline containing ketamine (100 mg/kg; Alfasan International B.V.) and xylazine (10 mg/kg; Alfasan International). The peritoneum was cut along the midline, and the left ureter was isolated and ligated twice using a 5-0 suture (NingBo Cheng-He Microsurgical Instruments). The bowel was laid back, and the peritoneum was closed with suture. Mice were placed under a heating lamp to maintain body temperature until recovery from anesthesia. For analgesia, a subcutaneous injection of 0.1 ml of saline containing buprenorphine (Temgesic, 0.05 mg/kg) was given to mice every 12 hours postsurgery. To verify the development of kidney tubulointerstitial fibrosis, mice were euthanized 3 days postsurgery, and the kidneys were weighted and processed for histological analysis.

### Ex vivo fluorescence imaging of hMSC biodistribution in UUO mice

hMSCs were seeded in a six-well plate until ~90% confluence was reached. To label the naïve hMSCs, Lipofectamine 3000–transfected hMSCs, and NW-transfected hMSCs with 1,1-dioctadecyl-3,3,3,3-tetramethylindotricarbocyanine iodide (DiR; Invitrogen, D12731), hMSCs were incubated with 1 ml of complete α-MEM containing a 10 μM DiR staining solution for 30 min at 37°C. After removing the dye solution and PBS rinses, hMSCs were trypsinized for cell counting by a hemocytometer, centrifuged at 1500 rpm for 5 min to pellet the cells, and resuspended in 0.2 ml of PBS. Three days post–UUO surgery, UUO mice received an intravenous injection of 0.2 ml of PBS containing DiR-labeled hMSCs at 5 × 10^6^ cells/kg body weight (*68*); as a control, some UUO mice received an intravenous injection of 0.2 ml of PBS containing free DiR dye (1 mg/kg body weight). Mice were euthanized 24 hours postinjection and perfused with PBS, and the internal organs (liver, lung, heart, spleen, and kidneys) were excised for fluorescence imaging using an Odyssey infrared imaging system (excitation wavelength: 700 nm; emission wavelength: ≥700 nm).

### Efficacy against kidney fibrosis

UUO mice received a single intravenous injection of 0.2 ml of PBS containing either (i) 5 × 10^5^ naïve hMSCs, (ii) 5 × 10^5^ hMSCs incubated with 0.5 mg of Au@PDA@lipid NWs, (iii) 5 × 10^5^ hMSCs transfected with Au@PDA@mCXCR4/mBMP7 NWs derived from loading 10 μg of total mRNA (i.e., 5 μg of mCXCR4 and 5 μg of mBMP7) on 0.5 mg of Au@PDA@lipid NWs, or (iv) 5 × 10^5^ hMSCs transfected with 10 μg of total mRNA by Lipofectamine 3000. For (iv), 10 μg of total mRNA (diluted in 100 μl of Opti-MEM) was added dropwise to 100 μl of transfection mixture (derived from mixing 10 μl of Lipofectamine 3000 stock with 90 μl of Opti-MEM); the final mixture was gently pipetted several times and incubated at RT for 10 min before incubation with hMSCs.

### Sample size calculation for efficacy against kidney fibrosis

We used Dunnett’s test to deduce the required size of each treatment group (*N*) (*69*). Dunnett’s test is a multiple comparison procedure that compares the efficacy of each treatment group with the same control group. Here, we tested “H0: All treatment groups are equivalent to the control group” against “H1: There exists one group that is superior to the control group.” We compared the treatment groups and the control group in a way that (i) the chance of committing type 1 error is <5% and that (ii) our comparison is of 80% power. Dunnett’s formalism states that *p* = √*N*·δ/σ, where *p* is the correlation coefficient that depends on *N*. There are three treatment groups for UUO mice (i.e., naïve hMSC, Lipo-transfected hMSC, and NW-transfected hMSC) and a control group (i.e., saline), so *p* is 4.3. If the superior treatment group gives an outcome (δ) of 1.5 SD (σ) better than the control group, the required *N* is (4.3/1.5)^2^ = 8.22 ≈ 8.

### Preparation of paraffin-embedded tissue sections

Major organs (heart, liver, spleen, lung, and kidneys) were fixed in 10% neutral buffered formalin (Sigma-Aldrich, HT501128) for 24 hours and stored in PBS at 4°C. Fixed tissues were dehydrated in ethanol, cleared in xylene, and embedded in paraffin blocks. Paraffin-embedded tissue sections (5 μm) were cut by a microtome (Thermo Fisher Scientific, HM325) and mounted on Superfrost Plus adhesion microscope slides (Epredia, J1800AMNZ).

### Histology

Tissue paraffin sections were deparaffinized in xylene (5 min × 3 times), rehydrated through a series of ethanol (100, 90, and 70%; 3 min × 2 times at each concentration) and Nanopure water (5 min × 3 times), and stained with Harris hematoxylin (Sigma-Aldrich, HHS16) for 3 min and eosin (Sigma-Aldrich, no. 45260) for 1 min. To evaluate tissue morphology, bright-field images were taken with a Nikon Eclipse Ti microscope.

### Immunohistochemistry

Deparaffinized and rehydrated tissue sections were immersed in antigen retrieval buffer (Beyotime) and heated in a microwave oven for 20 min. After cooling down, the slides were blocked with 2.5% normal horse serum (Vector Laboratories, S2012-50) for 2 hours and incubated with 60 μl of 2.5% normal horse serum containing primary antibodies [Col-I (1:400 dilution, Southern BioTech) and α-SMA (1:600 dilution; Abcam)] at 4°C overnight. Slides were washed with PBS, treated with 3% H_2_O_2_ (Merck Millipore) for 30 min, rinsed, and incubated with 60 μl of 2.5% normal horse serum containing secondary antibodies (ImmPRESS HRP Polymer Detection Kit, Vector Laboratories) for 30 min. The sections were developed sequentially using the 3,3′-diaminobenzidine (DAB) enzyme substrate (ImmPACT DAB, Vector Laboratories) for 2 min. Slides were counterstained with Mayer’s hematoxylin (Sigma-Aldrich, MHS1) for 3 min, washed with distilled water, dried in 90% ethanol, and mounted with DPX mountant (Sigma-Aldrich, no. 6522).

### Mouse model of APAP-induced ALI

Male mice were selected for the ALI model for their higher susceptibility to APAP-induced hepatotoxicity than age-matched female (*70*). Male C57BL/6 mice aged 10 to 12 weeks were fasted for 12 hours before APAP (Solarbio, IA0030) injection for inducing ALI. After receiving a single intraperitoneal injection of 0.3 ml of saline containing APAP (500 mg/kg; body weight), mice were allowed free access to food. To verify the establishment of ALI, mice were euthanized 24 hours post–APAP injection, serum was collected to measure the ALT level, and the liver was harvested for histological analysis.

### Blood pharmacokinetics

Mice received an intravenous injection of 0.2 ml of PBS containing 0.5 mg of Au@PDA@mHGF NW (loaded with 10 μg of mHGF). At different time points, ~0.5 ml of blood was withdrawn by terminal intracardiac puncture (using a 25-gauge needle) and stored inside EDTA-coated collection tubes (Becton Dickinson). The blood samples were digested by adding 1 ml of aqua regia and further diluted to 10 ml by the matrix solution (2% HCl and 2% HNO_3_) for ICP-MS measurements of the Au content. Data were fitted to a monoexponential decay model by using GraphPad Prism software.

### Inhibition of ClC3 in the ALI liver

siRNAs against ClC3 were obtained from Idobio. Two phosphorothioate linkages were introduced at both 5′ and 3′ ends of the sense and antisense strands of siRNA (underscored below), and two extra T’s were added as overhangs to enhance siRNA stability and nuclease resistance. siClC3 (2 nmol; diluted in 100 μl of PBS) was mixed with Lipofectamine 3000 (a mixture of 40 μl of Lipofectamine 3000 stock and 60 μl of PBS) by gentle pipetting and incubated at RT for 10 min before intravenous injection to the ALI mice. Sense (5′ → 3′): CACAGACGGAUCAACAGUAAATT. Antisense (5′ → 3′): UUUACUGUUGAUCCGUCUGUGTT.

### Confocal immunofluorescence in tissue sections

Fresh tissues were embedded in OCT (optimal cutting temperature) compound (Sakura, no. 4583) and stored at −80°C. Tissue cryosections of 8 μm in thickness were air dried for 20 min and fixed with 4% paraformaldehyde for 15 min at RT. Next, the cryosections were blocked with 2.5% horse serum at RT for 1 hour and stained with primary antibodies [HNF4α (1:100; Thermo Fisher Scientific, MA1199), Rab9 (1:100 dilution), or LAMP1 (1:150 dilution)] in 2.5% horse serum at 4°C overnight. After PBS rinses, the cryosections were stained with an Alexa Fluor 532–labeled, goat secondary antibody against mouse (1:1000; Invitrogen, A11002) in 2.5% horse serum at RT for 1 hour, stained with DAPI (1 μg/ml) in PBS for 10 min at RT, washed, and mounted with Antifade Mountant (Invitrogen, P36961) for confocal laser scanning microscopy. The excitation wavelengths of DAPI and Alexa Fluor 532 are 405 and 532 nm, respectively. The emission wavelengths of DAPI and Alexa Fluor 532 are 410 to 470 and 540 to 645 nm, respectively.

### Preparation of mHGF-loaded conventional liposomes

Soy phosphatidylcholine (Avanti Research) and cholesterol (Sigma-Aldrich) was mixed at a molar ratio of 85:15, dissolved in absolute ethanol to give a total lipid concentration of 100 mM, evaporated by nitrogen purging to form a thin lipid film, and vacuum dried at 40°C for 4 hours. After rehydrating the dried lipid film with 1 ml of mHGF stock solution (0.5 mg/ml) under stirring for 1 hour at 40°C, the suspension underwent three rounds of extrusion (2 × 10 times each round) through polycarbonate membranes (Nuclepore, Whatman) with pore sizes of 400, 200, and 100 nm using a miniextruder device (Avanti Research) to obtain the final homogeneous unilamellar liposomes. Free mRNA was removed by ultracentrifugation at 30,000*g* for 30 min at 4°C for three times, and the pellet was resuspended in nuclease-free water. Then, 100 μl of the supernatant was incubated in 100 μl of RiboGreen reagent working solution [from the Quant-it RiboGreen RNA assay kit (Invitrogen, R11490)] in the dark at RT for 5 min. The fluorescence of the mixture was determined by a microplate reader (excitation: 480 nm; emission: 520 nm). Encapsulation efficiency (%) was calculated using the following formula: [(Total mRNA added − Free mRNA)/Total mRNA added] × 100.

### Efficacy against ALI

ALI mice received a single intravenous injection of 0.2 ml of nuclease-free PBS containing either (i) 0.5 mg of Au@PDA@lipid NWs, (ii) Au@PDA@mHGF NWs derived from loading 10 μg of mHGF on 0.5 mg of Au@PDA@lipid NWs, (iii) Lipofectamine 3000 + mHGF [prepared by adding 10 μg of mHGF (in 100 μl of PBS) dropwise to 50 μl of transfection mixture (derived from mixing 20 μl of Lipofectamine 3000 stock and 30 μl of PBS), gentle pipette mixing for several times, incubation at RT for 10 min, and addition of 50 μl of PBS], (iv) PEI + mHGF [prepared by mixing 10 μg of mHGF (in 50 μl of mRNA-containing nuclease-free PBS), 50 μl of PEI (1 mg/ml; branched, 25 kDa), incubation at RT for 10 min, and dilution by 100 μl of PBS], and (v) conventional Lipo + mHGF with 10 μg of mHGF.

### ELISA

Tissues (~35 mg) were homogenized in 1.5 ml of precooled T-PER tissue protein extraction reagent containing Pierce protease inhibitors and phosphatase inhibitor cocktail (Bio-Platform) on ice. After incubating the homogenate on ice for 1 hour for complete lysis, the protein supernatant was collected by centrifugation at 15,000 rpm for 10 min at 4°C to remove any tissue debris. Aliquots (200 μl) of the supernatant were used for measuring the concentration of BMP7 in the UUO kidney by the Mouse BMP7 ELISA (enzyme-linked immunosorbent assay) Kit PicoKine (Boster Bio, EK1443) or the concentration of HGF in the ALI liver by the Mouse HGF ELISA Kit PicoKine (Boster Bio, EK1217).

### TUNEL assay staining

Apoptotic and necrotic cells were visualized and quantified using the Alexa Fluor 488 Click-iT Plus TUNEL (terminal deoxynucleotidyl transferase–mediated deoxyuridine triphosphate nick end labeling) Assay for In Situ Apoptosis Detection (Invitrogen, C10617). Liver paraffin sections of 5 μm in thickness were stained per the manufacturer’s instructions, costained with DAPI (1 μg/ml) in PBS for 10 min at RT, and mounted with Antifade Mountant for confocal laser scanning microscopy. The excitation wavelengths of DAPI and Alexa Fluor 488 are 405 and 488 nm, respectively. The emission wavelength ranges of DAPI and Alexa Fluor 488 are 415 to 480 and 495 to 650 nm, respectively.

### Sample size calculation for efficacy against ALI

We used Dunnett’s test to deduce the required size of each treatment group (*N*) (*69*). For efficacy data shown in the main text, there are three treatment groups for ALI mice (i.e., Au@PDA@lipid NW, Au@PDA@mHGF NW, and Lipo + mHGF) and a control group (i.e., saline), so *p* is 4.3. If the superior treatment group gives an outcome (δ) of 1.5 SD (σ) better than the control group, the required *N* is (4.3/1.5)^2^ = 8.22 ≈ 8. For efficacy data shown in the Supplementary Materials, there is one treatment group for each benchmark (PEI + mHGF or conventional Lipo + mHGF) and a control group (saline), so *p* is 3.52. Therefore, the required *N* is (3.52/1.5)^2^ = 5.51 ≈ 5.

### In vivo toxicity of Au@PDA@mHGF NW

#### Blood test

ALI mice received a single intravenous injection of 0.15 ml of PBS containing 0.5 mg of Au@PDA@mHGF NWs (with 10 μg of mHGF). Twenty-four hours later, 1 ml of whole blood was collected via intracardiac puncture under anesthesia, with 0.5 ml collected in an EDTA-coated collection tube (Becton Dickinson) for the enumeration of blood cells and the remaining 0.5 ml collected in a 1.7-ml centrifuge tube. About 200 μl of serum was extracted following centrifugation for the subsequent analysis of serum levels of ALT (alanine transaminase) and AST (aspartate transaminase). All samples were sent to the PathLab Medical Laboratories (Hong Kong) on the same day for blood biochemistry analysis.

#### Long-term toxicity and clearance

ALI and healthy C57 mice received a single intravenous injection of 0.15 ml of PBS containing 0.5 mg of Au@PDA@mHGF NWs (loaded with 10 μg of mHGF). Mice were maintained for 7 and 8 months. Blood samples were collected and sent out for analysis at the PathLab Medical Laboratories. Major internal organs and blood were collected and subjected to digestion in aqua regia for ICP-MS analysis.

### Data processing and statistical analysis

Prism (GraphPad) software was used for data analysis and graph construction. To determine the statistical significance in the comparison of two groups, an unpaired two-tailed *t* test was performed. To determine the statistical significance in the comparison of multiple groups, an unpaired one-way analysis of variance (ANOVA) was performed with Tukey’s test for post hoc analysis. The normality of sampling distribution of means was validated by the Shapiro-Wilk test. The homogeneity of variance was validated by Bartlett’s test. Results are considered significant at *P* < 0.05.

## Supplementary Materials

### The PDF file includes:

Figs. S1 to S64 Tables S1 to S10 Legends for data S1 and S2 References
