# Supplementary material for: A worm-like nucleic acid nanostructure for gene delivery and endosomal escape via ClC3 ion exchanger
Source: Sci Adv. 2026 Mar 6;12(10):eadw0891. doi: 10.1126/sciadv.adw0891 (PMC13150675; doi:10.1126/sciadv.adw0891)
Supplement: Supplementary file 1 — Figs. S1 to S64 Tables S1 to S10 Legends for data S1 and S2 References [file sciadv.adw0891_sm.pdf]

Science Advances
AAAS

Supplementary Materials for

A worm-like nucleic acid nanostructure for gene delivery and endosomal escape via ClC3 ion exchanger

Yu Xiao *et al.*

Corresponding author: Chung Hang Jonathan Choi, jchchoi@cuhk.edu.hk

Sci. Adv. **12**, eadw0891 (2026)
DOI: 10.1126/sciadv.adw0891

The PDF file includes:

Figs. S1 to S64
Tables S1 to S10
Legends for data S1 and S2
References

Other Supplementary Material for this manuscript includes the following:

Data S1 and S2

Supplementary Figures

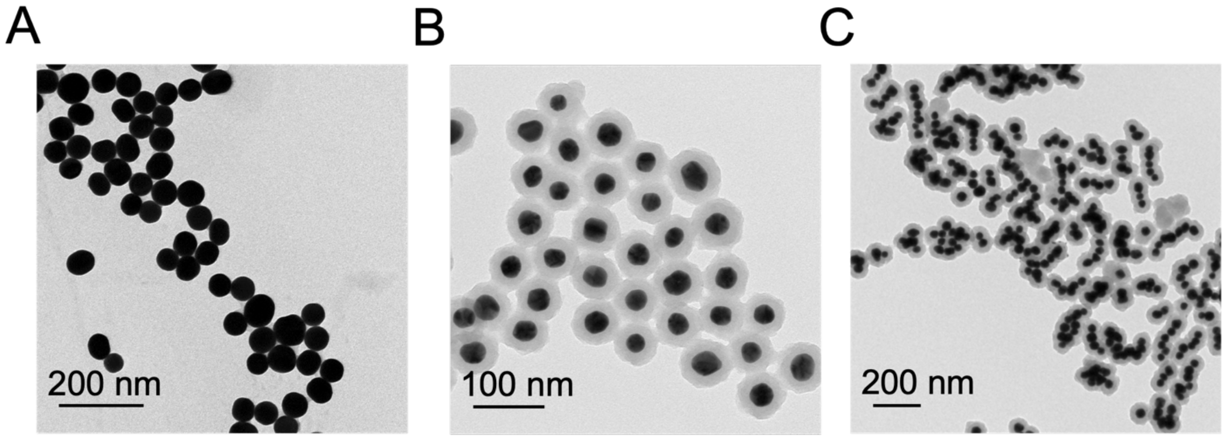

Fig. S1. Representative TEM images of (**A**) citrate-capped 40 nm Au NPs, (**B**) unassembled Au@PDA NPs, (**C**) self-assembled Au@PDA NWs. While our method of 1D assembly does not permit precise control over the number of Au cores per NW, it yields mostly single-chained Au@PDA NWs with reasonable structural uniformity without postsynthesis separation steps like differential centrifugation (*71*). By counting ~500 Au@PDA NWs under TEM, the average core number is 4–5 and the average physical dimension of Au@PDA NW is (200.1 ± 6.3) nm × (78.4 ± 3.8) nm (length × width).

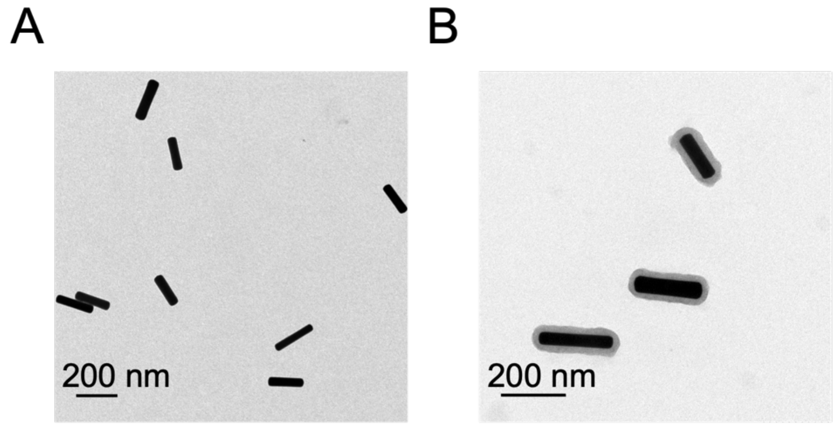

Figure S2. Representative TEM images of (**A**) citrate-capped Au NRs (200 nm × 45 nm) and (**B**) Au@PDA NRs. By counting ~500 Au@PDA NRs under TEM, the average physical dimension of Au@PDA NR is (220.1 ± 4.7) nm × (83.1 ± 4.9) nm (length × width).

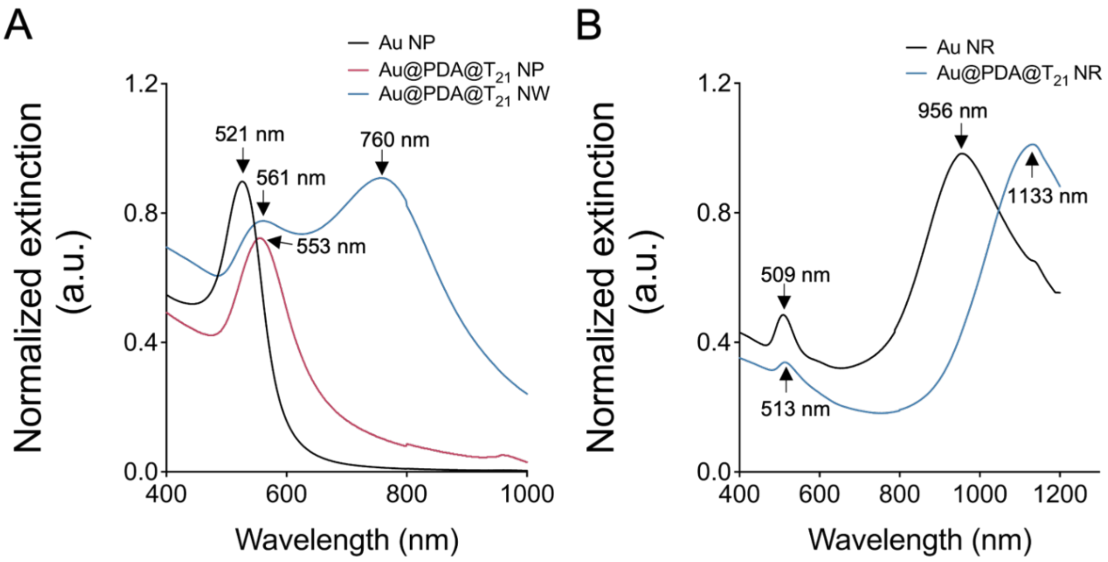

Figure S3. UV-Vis spectra of (**A**) citrate-capped Au NP (~40 nm), unassembled Au@PDA@T_{21} NP, and self-assembled Au@PDA@T_{21} NW, and (**B**) citrate-capped Au NR (200 nm × 45 nm), and Au@PDA@T_{21} NR. Arrow indicates the localized surface plasmon resonance (LSPR) peaks and number indicates the LSPR wavelengths.

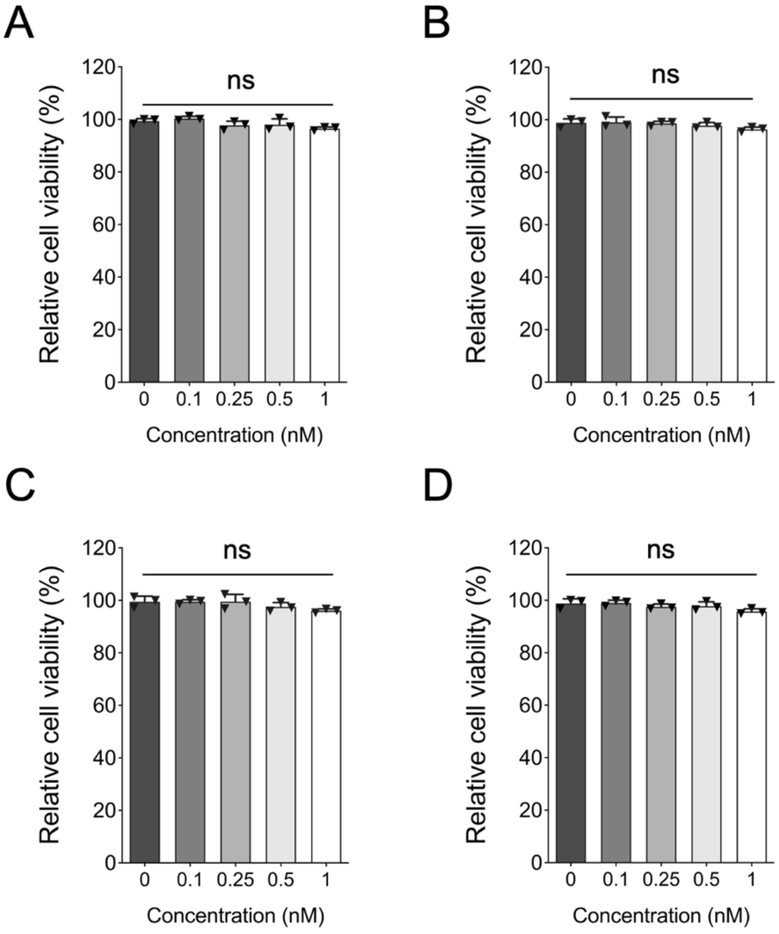

Figure S4. Cytotoxicity of oligonucleotide-encased NWs in vitro. (**A**) Au@PDA@T_{21} NWs, (**B**) Au@PDA@asEGFP NWs, (**C**) Au@PDA@miR-223 NWs, and (**D**) Au@PDA@siNog NWs were incubated with A549 cells, bEnd.3 cells, BMDMs, and hMSCs for 24 h, respectively. By the alamarBlue assay, the cells remained largely viable at all concentration tested. Data are presented as mean ± SEM. Statistical significance was calculated by Student's t-test. ns: not significant ($P > 0.05$). n = 3 biological replicates per group, across 1 experiment.

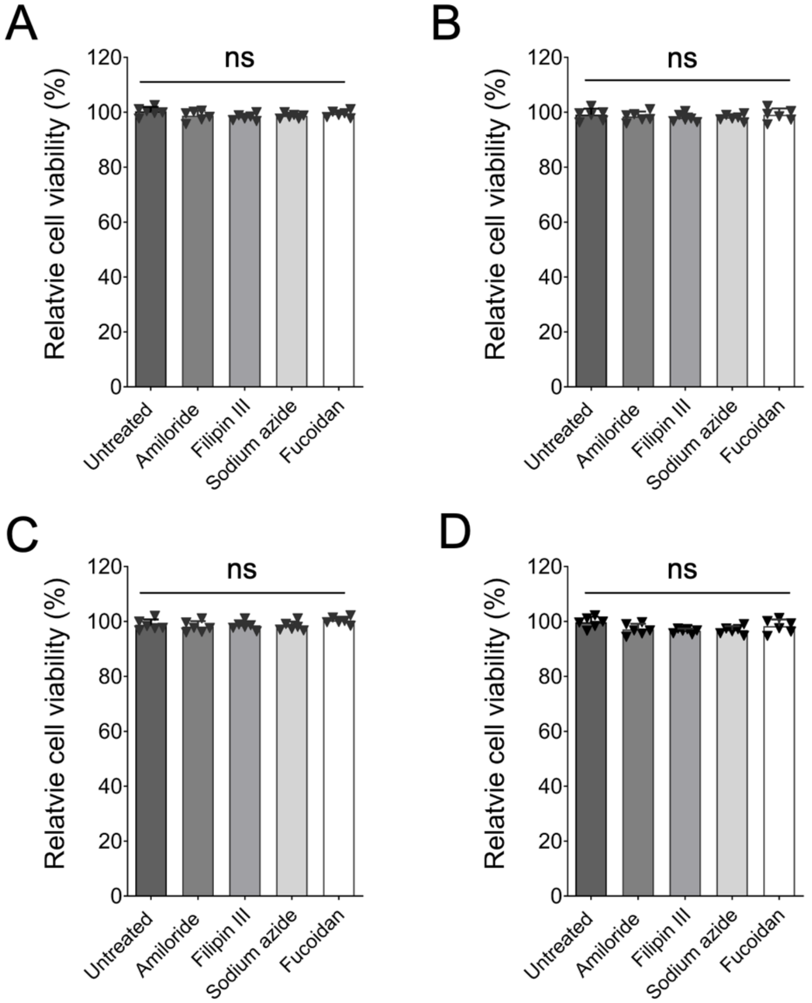

Figure S5. Cytotoxicity of pharmacological inhibitors. (**A**) A549 cells, (**B**) bEnd.3 cells, (**C**) BMDMs, and (**D**) hMSCs were incubated with various inhibitors of cellular uptake pathways for 4 h, including amiloride (an inhibitor of macropinocytosis), filipin III (an inhibitor of caveolae/lipid raft-mediated endocytosis), sodium azide (an inhibitor of energy-dependent endocytosis), and fucoidan (an inhibitor of scavenger receptor A-mediated endocytosis). By the alamarBlue assay, the cells remained largely viable after incubation with all the inhibitors tested. Data are presented as mean ± SEM. Statistical significance was calculated by Student's t-test. ns: not significant ($P > 0.05$). $n = 6$ biological replicates per group, across 1 experiment.

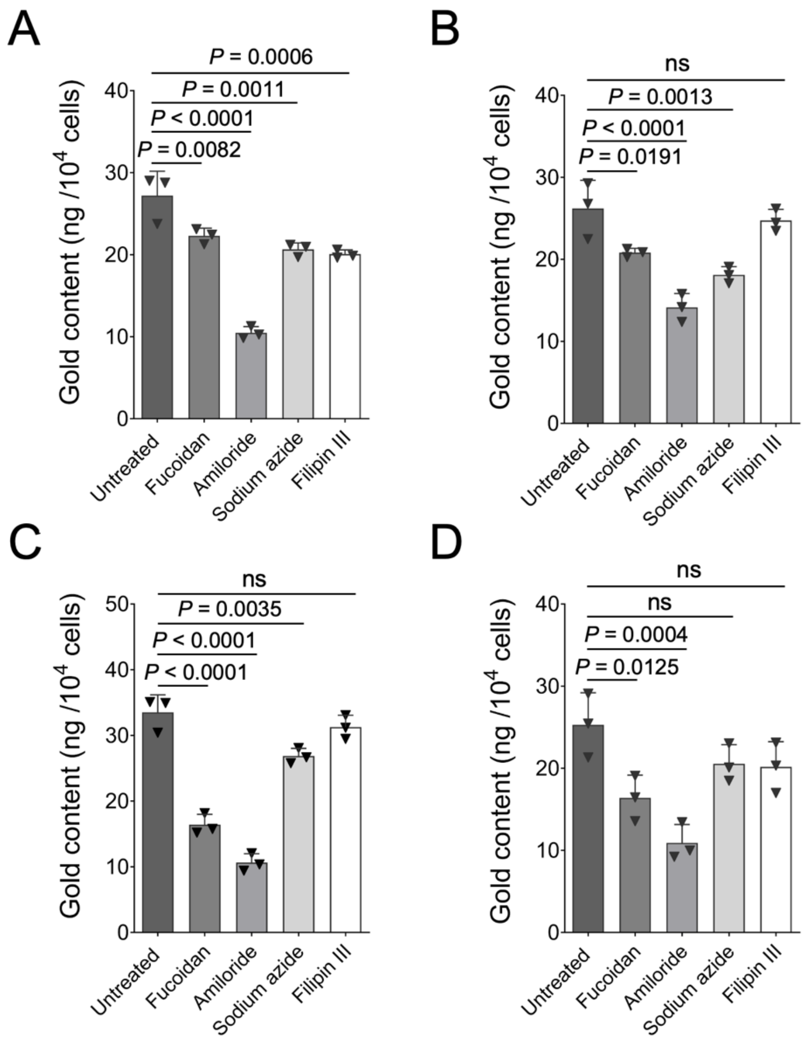

Figure S6. Cellular uptake pathway of oligonucleotide-encased NWs. A549 cells, bEnd.3 cells, BMDMs, and hMSCs were pre-incubated with various inhibitors of cellular uptake pathways for 1 h. Then the (**A**) A549 cells were co-incubated with Au@PDA@T_{21} NW, (**B**) bEnd.3 cells were co-incubated with Au@PDA@asEGFP NW, (**C**) BMDMs were co-incubated with Au@PDA@miR-223 NW, and (**D**) hMSCs were co-incubated Au@PDA@siNog NW for another 4 h. The level of cellular uptake was measured by ICP-MS. Data are presented as mean ± SEM. Statistical significance was calculated by Student's t-test. ns: not significant (P > 0.05). n = 3 biological replicates per group, across 1 experiment.

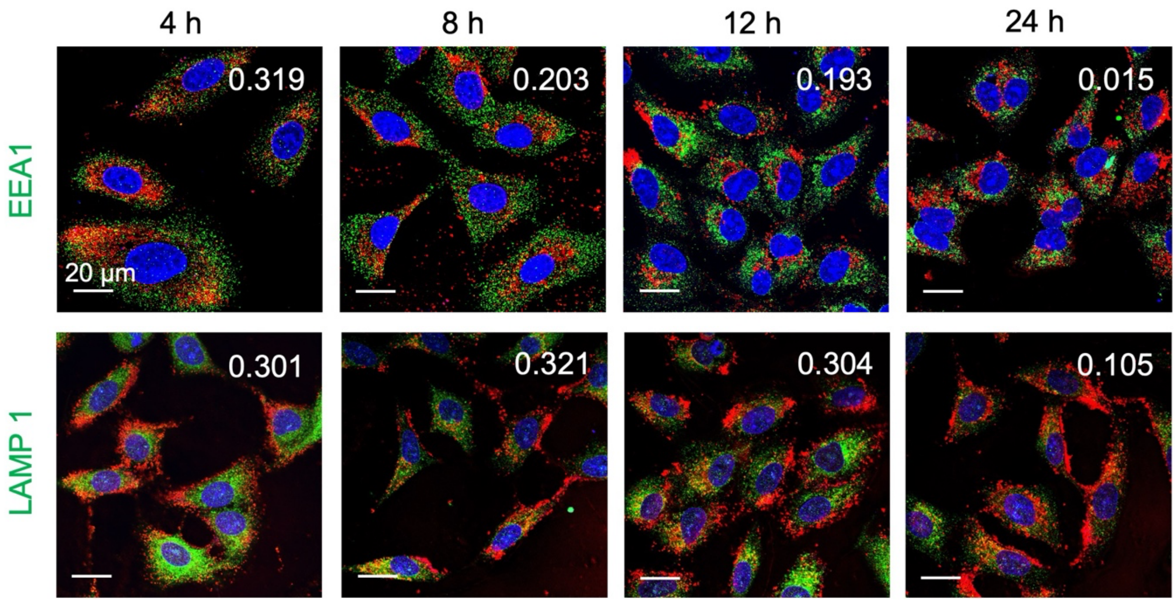

Figure S7. Intracellular trafficking of Cy5-labeled Au@PDA@T_{21} NWs in A549 cells as a function of incubation time. Time-lapse confocal images showed that Au@PDA@T_{21} NWs do not highly colocalize with early endosomes and lysosomes. EEA1: a marker of early endosome; LAMP1: a marker of lysosome. Blue = DAPI (nuclei). White number indicates Pearson colocalization coefficient (PCC) between Cy5-labeled Au@PDA@T_{21} NWs (red) and the marker of vesicle (green).

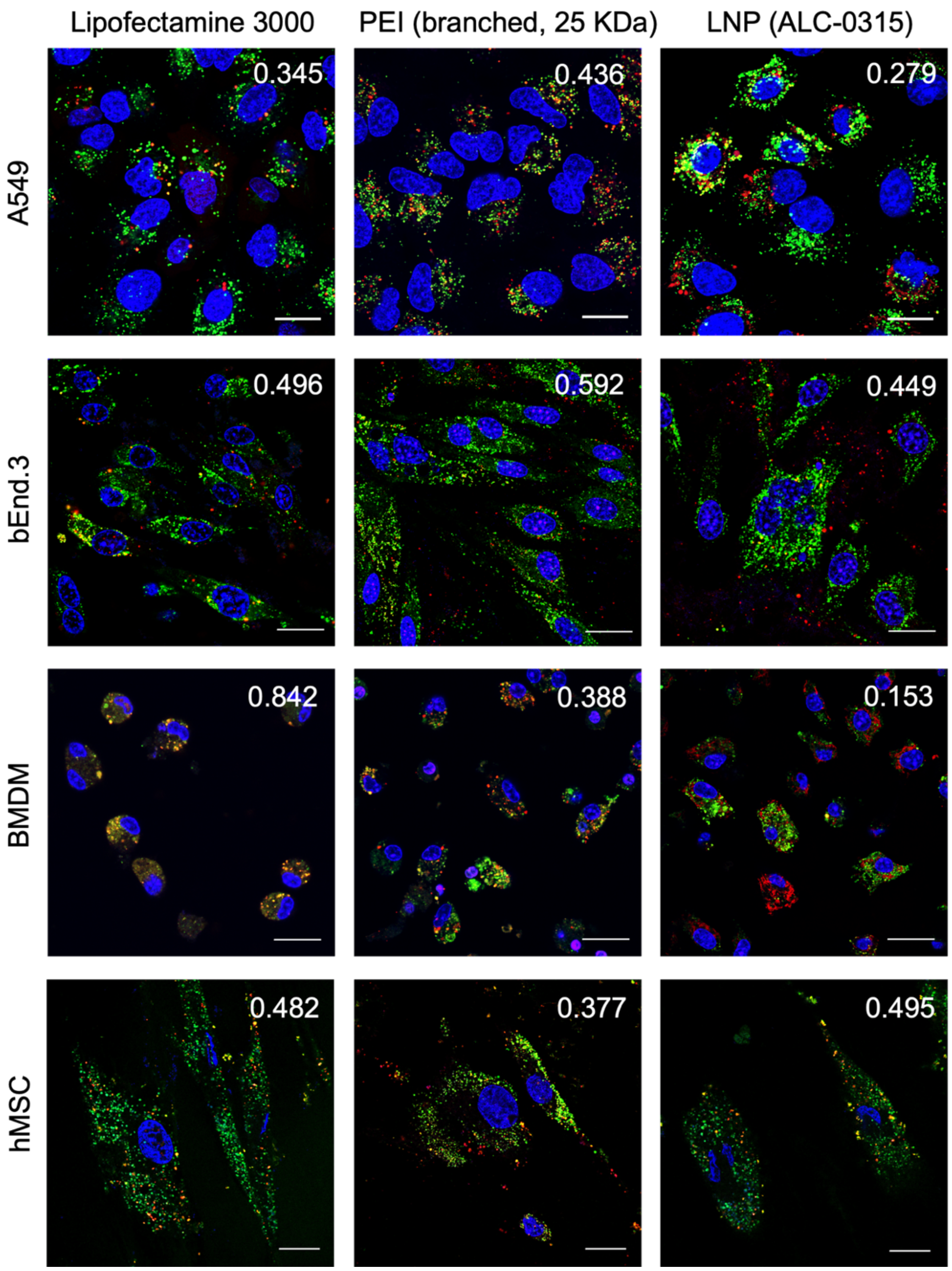

Figure S8. Endosomal escape of conventional gene carriers for delivering T_{21} DNA in vitro. A549 cells, bEnd.3 cells, BMDM, and hMSC were treated with Lipofectamine 3000, PEI (branched, 25 KDa), and LNP (formed with ionizable lipid ALC-0315) containing the same amount of Cy5-labeled T_{21} as Au@PDA@T_{21} NW for 24 h and then were stained with LysoTracker. No conventional gene carrier could deliver a PCC < 0.2 for all four cell types. Blue = nuclei. White number indicates PCC between Cy5-labeled gene carrier-T_{21} complexes (red) and LysoTracker (green). Scale bar = 20 μm.

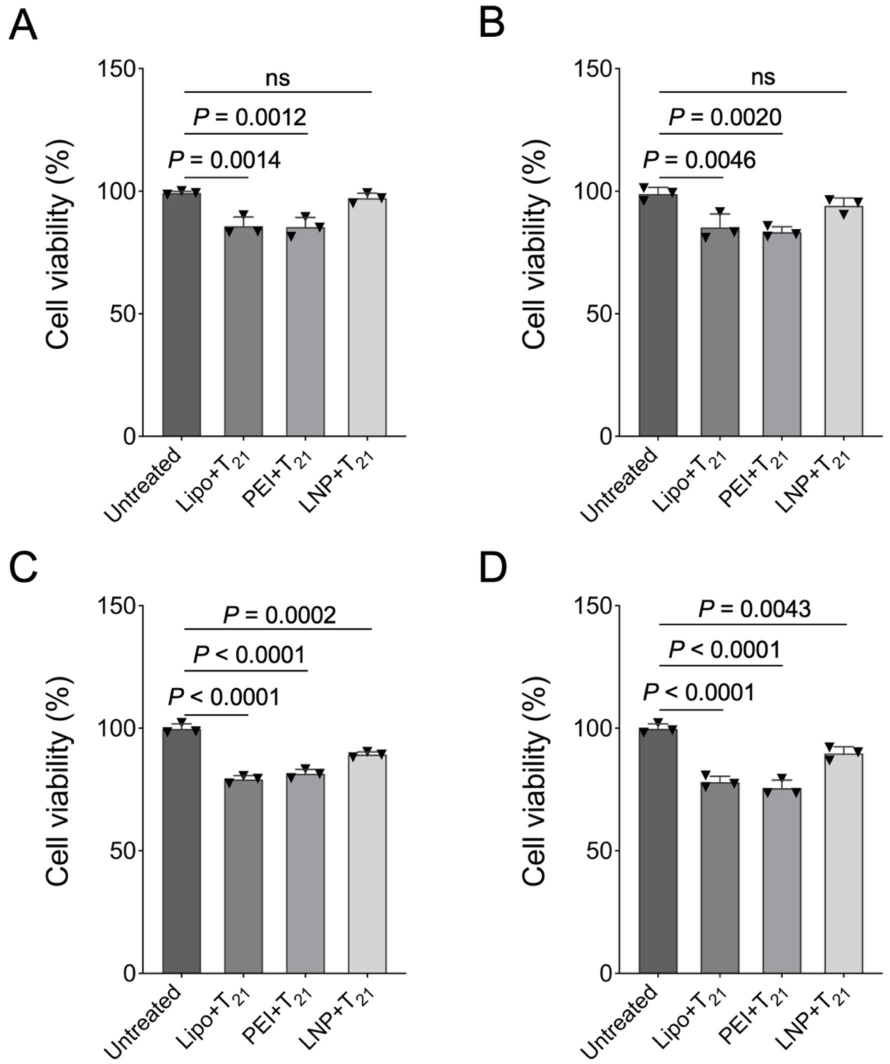

Figure S9. Cytotoxicity of conventional gene carriers for delivering T_{21} DNA in vitro. (**A**) A549 cells, (**B**) bEnd.3 cells, (**C**) BMDMs, and (**D**) hMSCs were incubated with Lipo+T_{21}, PEI+T_{21}, and LNP+T_{21} for 24 h. By alamarBlue assay, ionizable LNP reduced the viability of primary BMDMs and hMSCs, not A549 and bEnd.3 cells. Lipofectamine 3000 and PEI reduced the viability of all four cell types tested. Data are presented as mean ± SEM. Statistical significance was calculated by Student's t-test. ns: not significant ($P > 0.05$). n = 3 biological replicates per group, across 1 experiment.

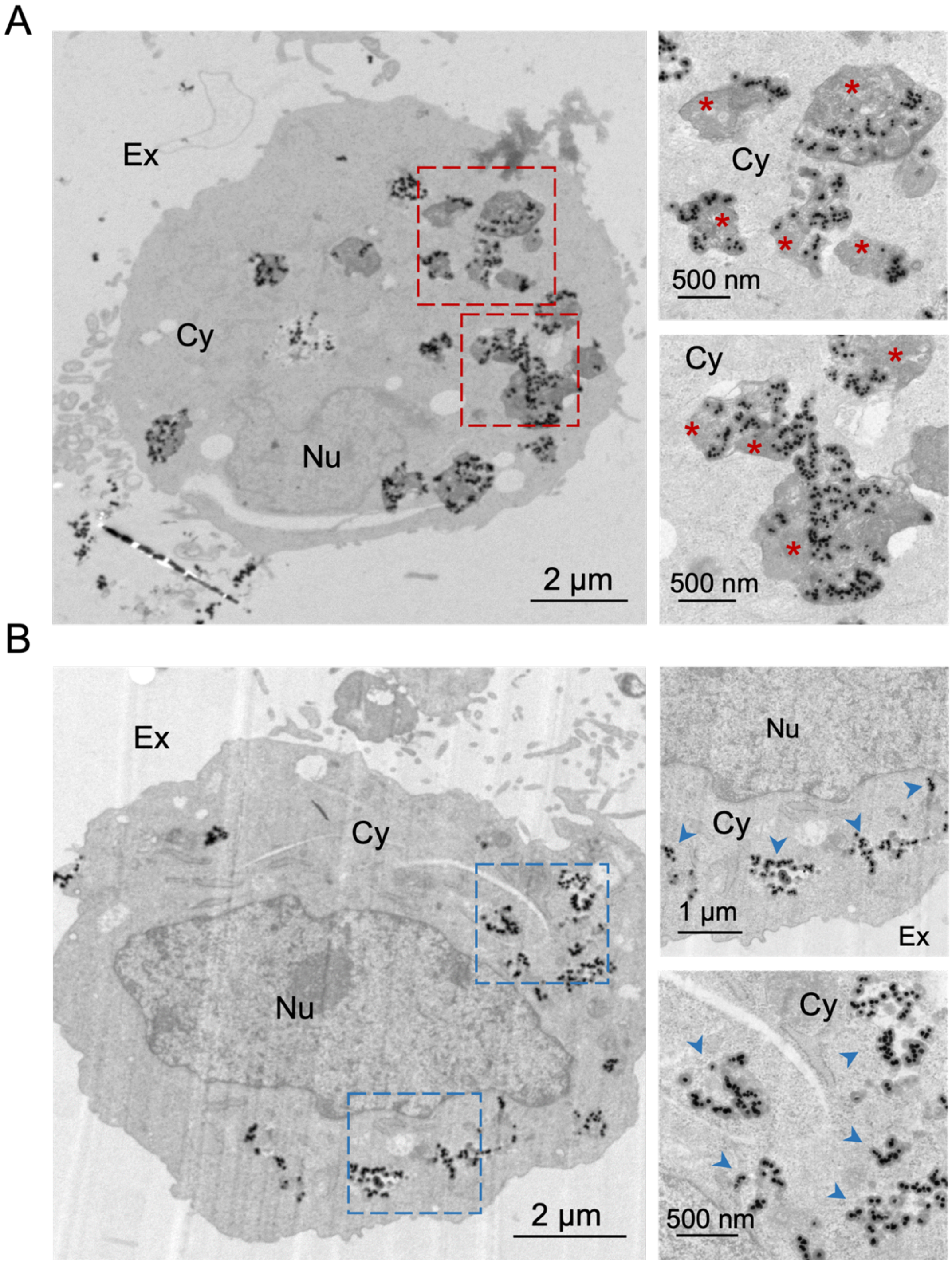

Figure S10. Representative TEM images of Au@PDA@T_{21} NWs in A549 cells. Most Au@PDA@T_{21} NWs were (**A**) entrapped in late endosomes 8 h post-incubation and (**B**) accumulated freely in the cytosol (or outside of vesicles) 24 h post-incubation. Nu = Nucleus. Cy = cytosol. Ex = Extracellular space. Red asterisks denote vesicles, and blue arrows denote free Au@PDA@T_{21} NWs in the cytosol. (**A** and **B**) The two smaller images on the right show the enlargement of the boxed areas (red or blue) of the larger image on the left.

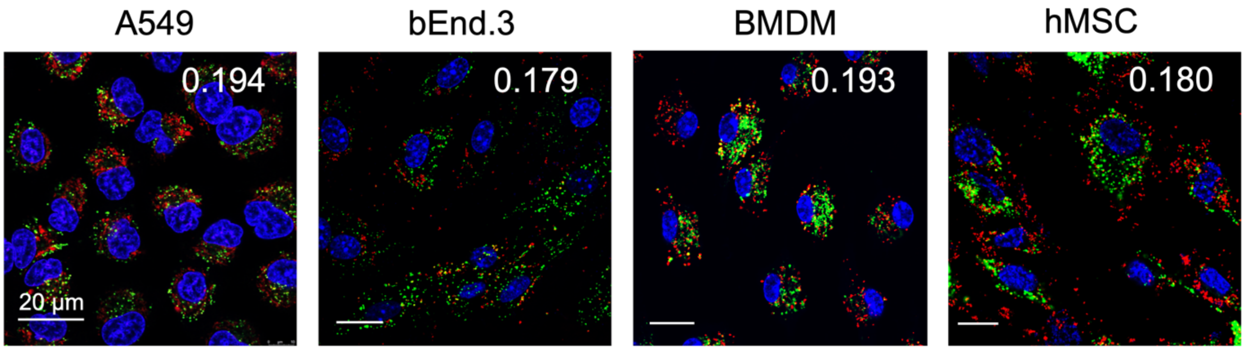

Figure S11. Intracellular trafficking of Cy5-labeled Au@PDA@A_{21} NWs in different types of cells 24 h post-incubation. Confocal images showed that Au@PDA@A_{21} NWs have limited colocalization with lysosomes, consistent with the results for Au@PDA@T_{21} NWs as shown in Figure 1. Blue = nuclei. White number indicates PCC between Cy5-labeled Au@PDA@A_{21} NWs (red) and LysoTracker (green). Scale bar = 20 μm.

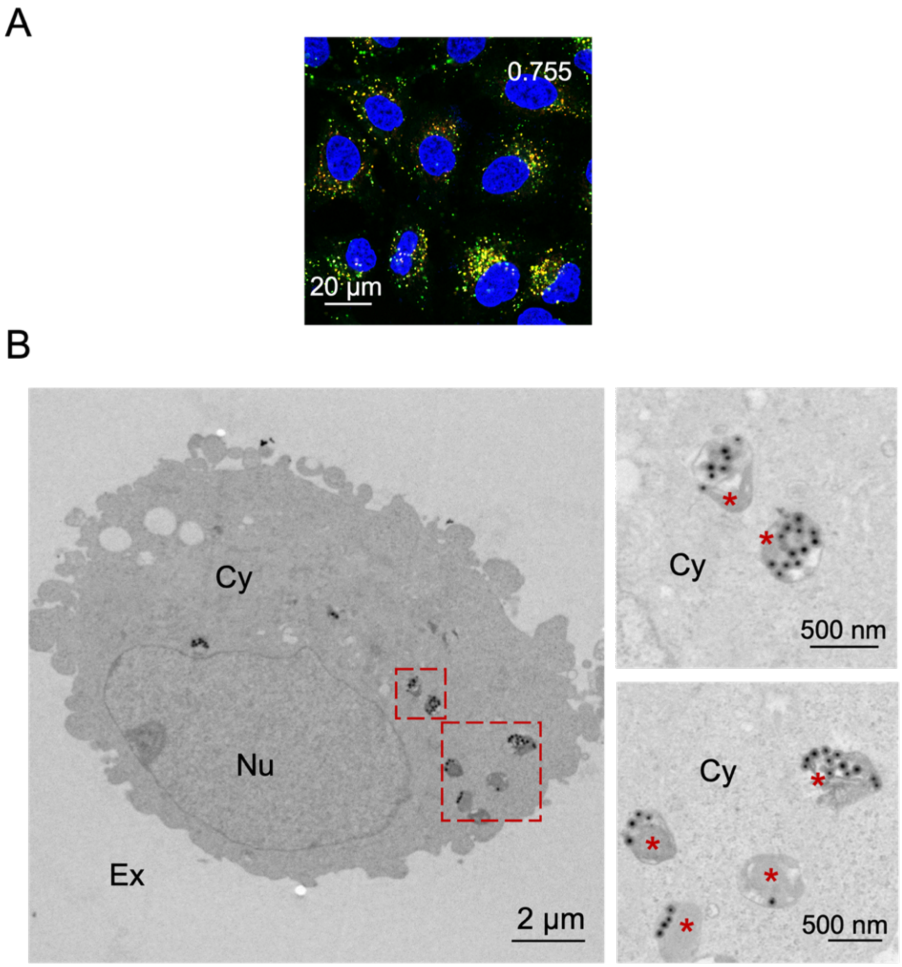

Figure S12. Confocal and TEM images of unassembled Au@PDA@T_{21} NPs in A549 cells. (**A**) Cy5-labeled Au@PDA@T_{21} NPs (red) were highly colocalized with LysoTracker 24 h post-

incubation. Blue = DAPI (nuclei). White number indicates PCC between Au@PDA@T_{21} NPs and LysoTracker (green). (**B**) TEM images confirmed most Au@PDA@T_{21} NPs were entrapped in the intracellular vesicles 24 h post-incubation. Nu = nucleus. Cy = cytosol. Ex = Extracellular space. Red asterisks denote intracellular vesicles. The two smaller images on the right show the enlargement of the boxed areas of the larger image on the left.

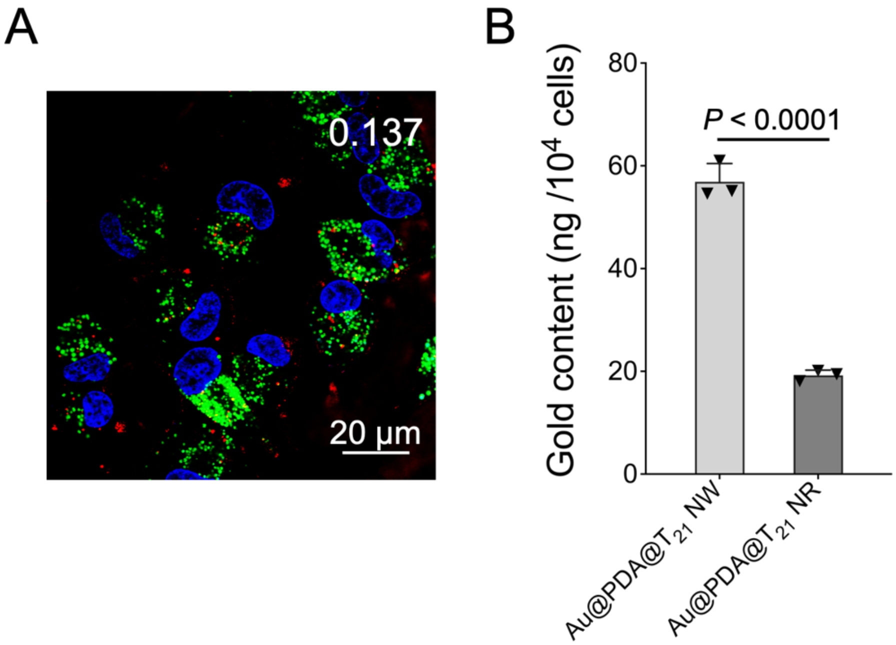

Figure S13. Endosomal escape and cellular uptake of Au@PDA@T_{21} NRs in A549 cells. (**A**) Colocalization of Au@PDA@T_{21} NRs in A549 cells 24 h post-incubation. Blue = nuclei. White number indicates PCC between Cy5-labeled Au@PDA@T_{21} NRs (red) and LysoTracker (green). (**B**) ICP-MS measurement showed that the uptake of Au@PDA@T_{21} NR was ~65% less than that of Au@PDA@T_{21} NW. The particle concentration (0.1 nM, in terms of the entity of NW or NR) added to the cells was kept the same. Data are presented as mean ± SEM. Statistical significance was calculated by Student's t-test. n = 3 biological replicates per group, across 1 experiment.

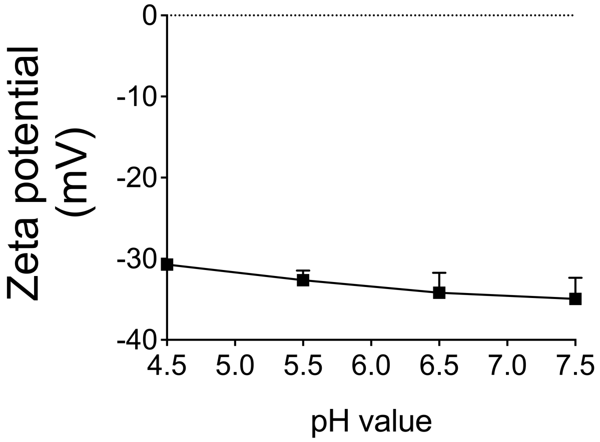

Figure S14. Zeta potentials of Au@PDA@T_{21} NW as a function of pH. Au@PDA@T_{21} NW remained negatively charged between pH 4.5 and pH 7.5. Data are presented as mean ± SEM. n = 3 biological replicates per group, across 1 experiment.

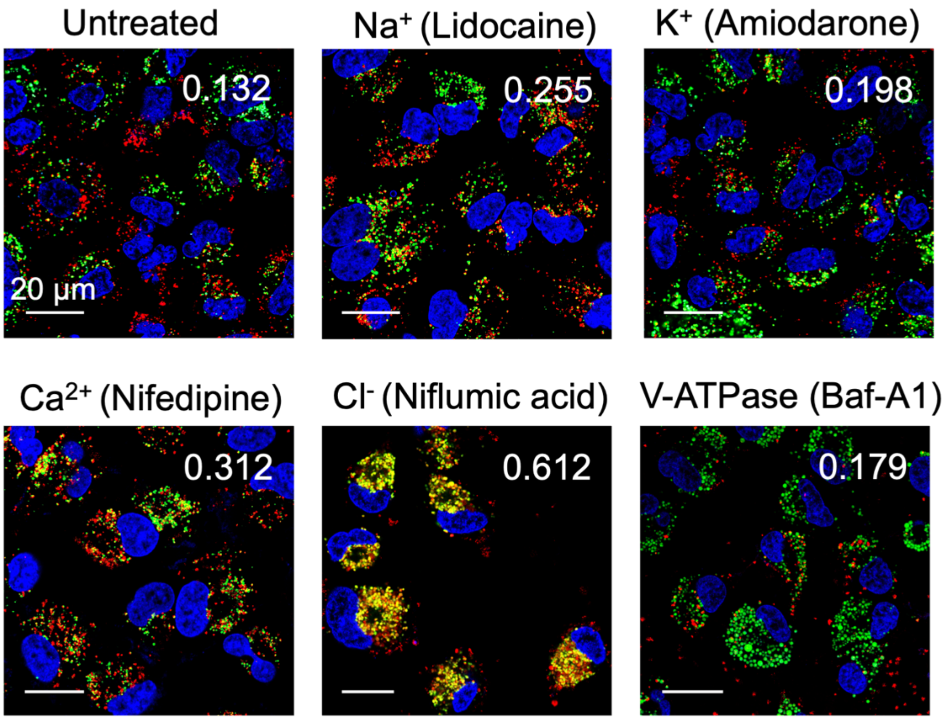

Figure S15. Influence of Na^+, K^+, Ca^{2+}, Cl^- channels and V-ATPase on the endosomal escape of Au@PDA@T_{21} NW in A549 cells. White number indicates PCC between Cy5-labeled Au@PDA@T_{21} NWs (red) and LysoTracker (green). Niflumic acid (a general Cl^- channel blocker) disrupted the endosomal escape of Au@PDA@T_{21} NWs, but other blockers did not 24 h post-incubation. Baf-A1: bafilomycin A1. Blue = nuclei. Scale bar = 20 µm.

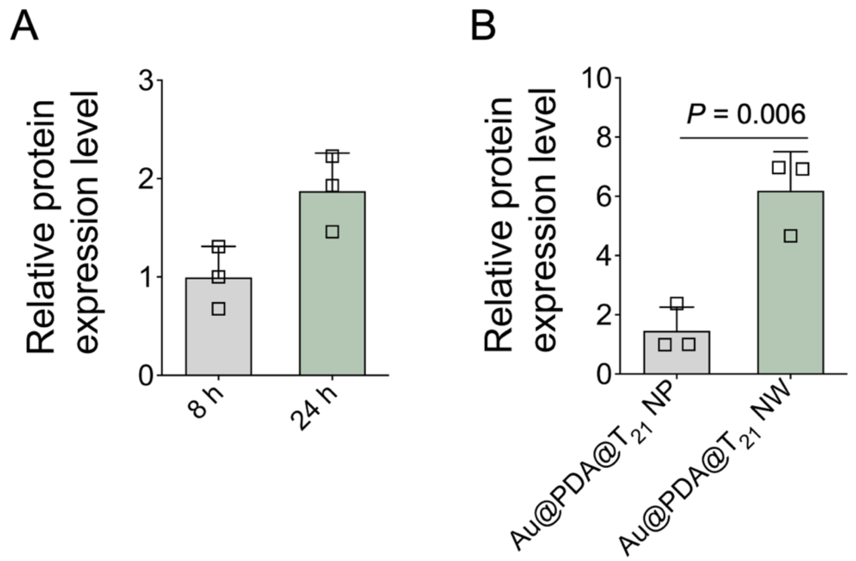

Figure S16. Quantification of western blot data of ClC3 expression in A549 cells under two pairwise comparisons, including (**A**) time-dependent comparison, "Au@PDA@T_{21} NW 8 h" vs. "Au@PDA@T_{21} NW 24 h" and (**B**) shape-dependent comparison, "Au@PDA@T_{21} NP 24 h" vs. "Au@PDA@T_{21} NW 24 h". Data are presented as mean ± SEM. Statistical significance was calculated by Student's t-test. n = 3 biological replicates per group, across 1 experiment. The original western blot is shown in Fig. 2G.

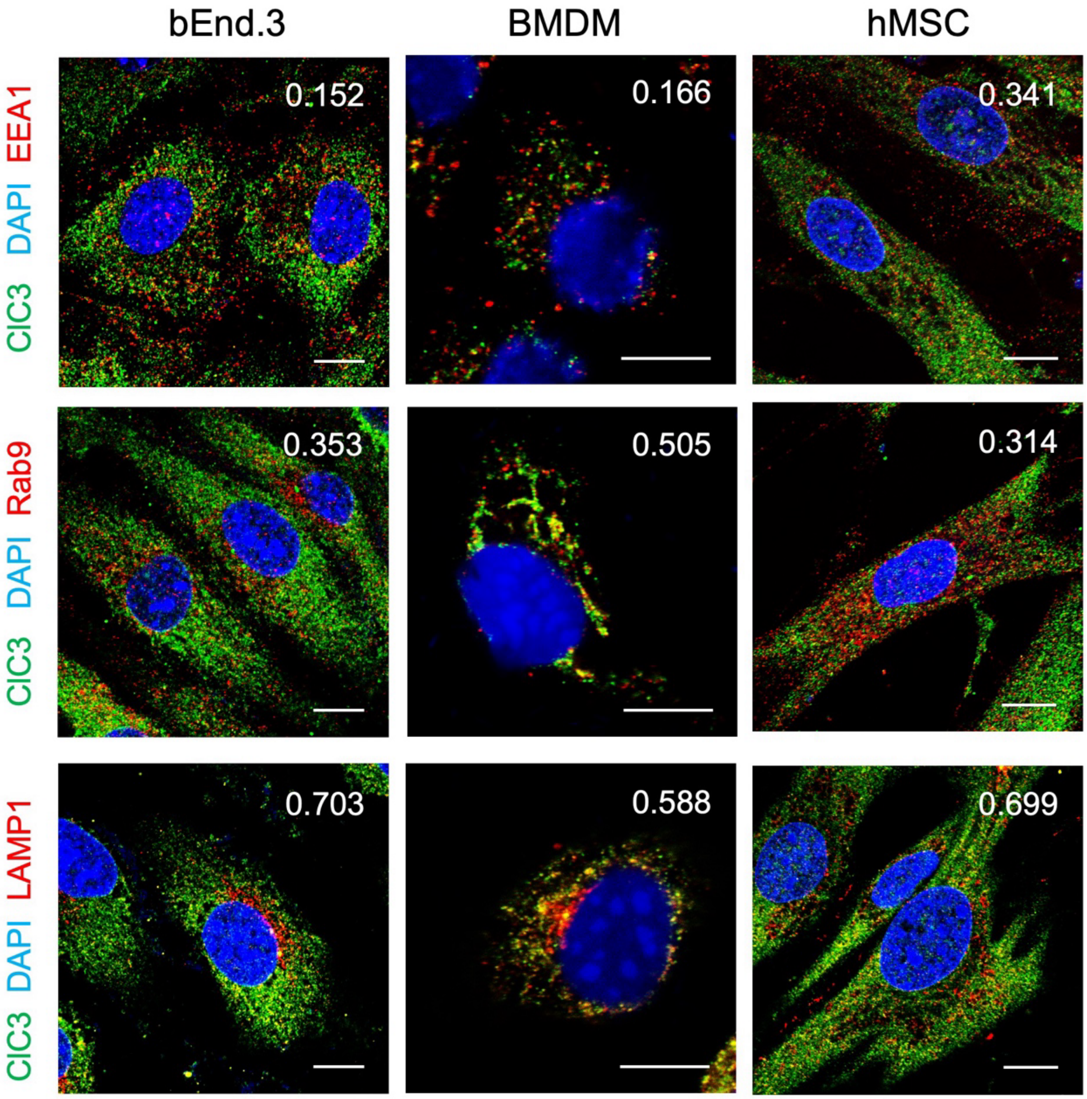

Figure S17. Subcellular location of ClC3 relative to intracellular vesicles in bEnd.3 cells, BMDMs, and hMSCs. ClC3 was strongly colocalized with lysosomes (LAMP1) and, to a lesser extent, late endosomes (Rab9). ClC3 has little colocalization with early endosomes (EEA1). White number indicates PCC between ClC3 (green) and intracellular vesicles (red). Blue = nuclei. Scale bar = 10 μm.

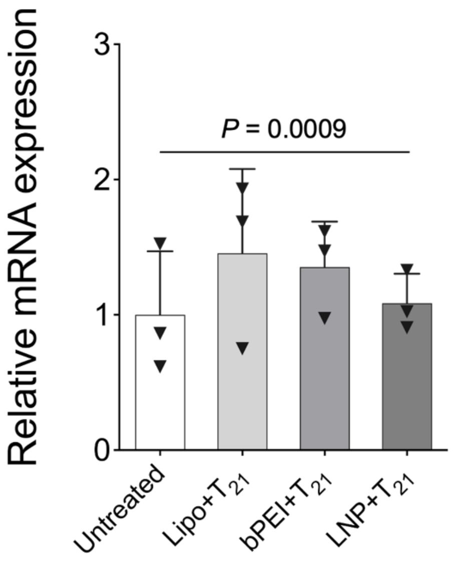

Figure S18. qRT-PCR measurement of ClC3 expression in A549 cells. A549 cells were incubated with conventional gene carriers for delivering T_{21} DNA to A549 cells for 24 h. qRT-PCR analysis of the treated cells showed no upregulation of ClC3. Data are presented as mean ± SEM. Statistical significance was calculated by Student's t-test. ns: not significant ($P > 0.05$). n = 3 biological replicates per group, across 1 experiment.

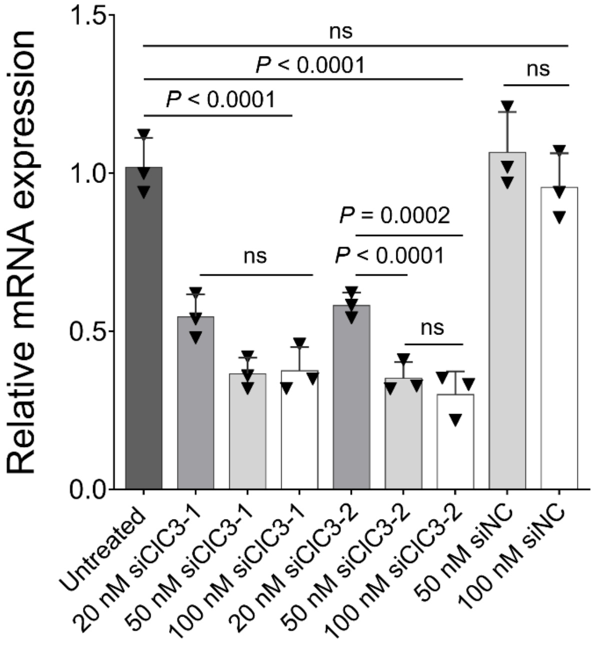

Figure S19. Validation of ClC3 knockdown in A549 cells. Cells were incubated with two different siClC3 sequences at three concentrations using Lipofectamine 3000. Relative mRNA expression of ClC3 was assessed 48 h post-incubation by qRT-PCR. A scrambled siRNA sequence (siNC) served as a negative control. Data are presented as mean ± SEM. Statistical significance was calculated by one-way ANOVA with Tukey's Test for post-hoc analysis. ns = not significant ($P > 0.05$). n = 3 biological replicates per group, across 1 experiment.

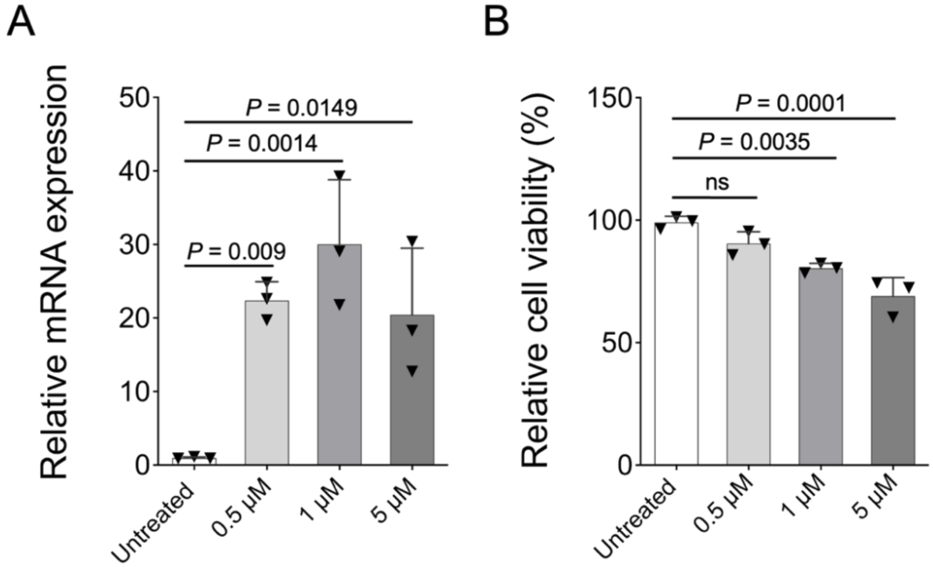

Figure S20. Validation of ClC3 activation by bufalin in A549 cells. (**A**) Relative mRNA expression of ClC3 and (**B**) cell viability after incubation with different concentrations of bufalin for 8 h. We chose 0.5 µM bufalin for our subsequent studies due to its effective upregulation of ClC3 and low cytotoxicity. Data are presented as mean ± SEM. Statistical significance was calculated by Student's t-test. ns = not significant (P > 0.05). n = 3 biological replicates per group, across 1 experiment.

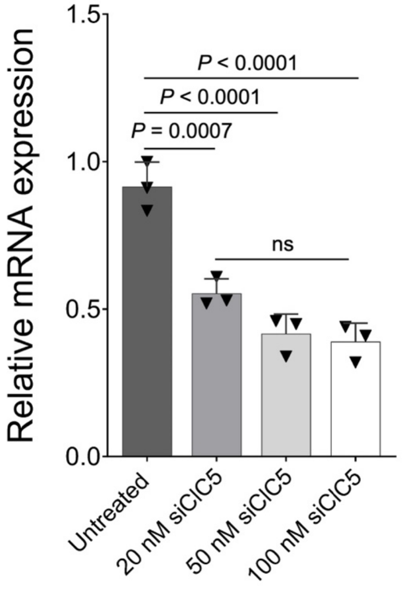

Figure S21. Validation of ClC5 knockdown in A549 cells. Cells were incubated with siClC5 sequences at three concentrations using Lipofectamine 3000. Relative mRNA expression of ClC3 was assessed 48 h post-incubation by qRT-PCR. Data are presented as mean ± SEM. Statistical significance was calculated by one-way ANOVA with Tukey's Test for post-hoc analysis. ns = not significant (P > 0.05). n = 3 biological replicates per group, across 1 experiment.

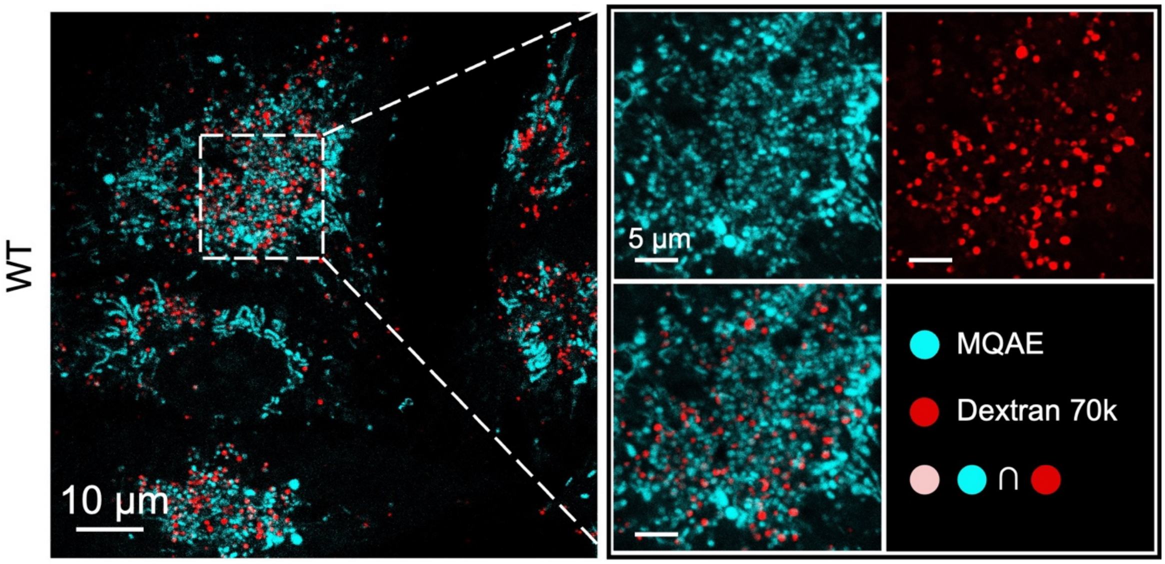

Figure S22. Cl^- concentration in intracellular vesicles labeled by dextran 70K (red) in wildtype A549 cells 8 h post-incubation. The NW-free vesicles in the wildtype cells did not severely quench MQAE fluorescence (cyan).

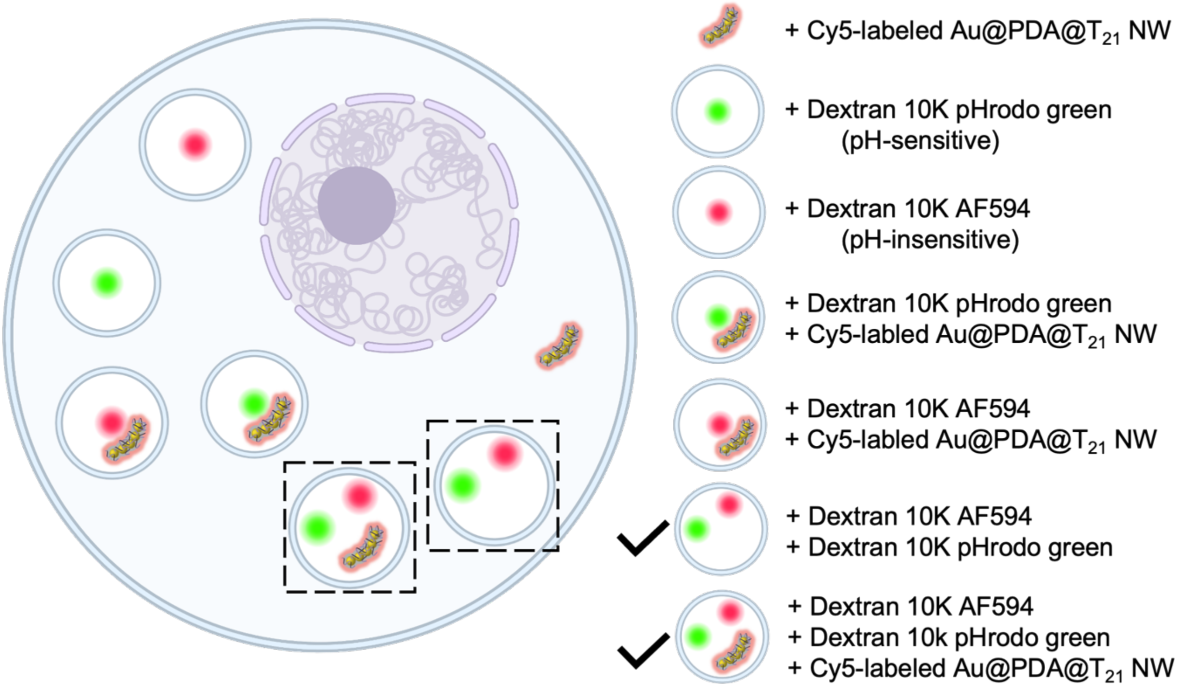

Figure S23. Experimental design of pH measurement of intracellular vesicles. A549 cells were incubated with a mixture of dextran 10K pHrodo green, dextran 10K Alexa Fluor (AF) 594, and Cy5-labeled Au@PDA@T_{21}NW for 8 h. Only intracellular vesicles containing both dextran 10K AF594 and dextran 10K pHrodo green dyes were subjected to pH measurements. Created in BioRender. Choi, J. (2025) https://BioRender.com/t4d0rf1.

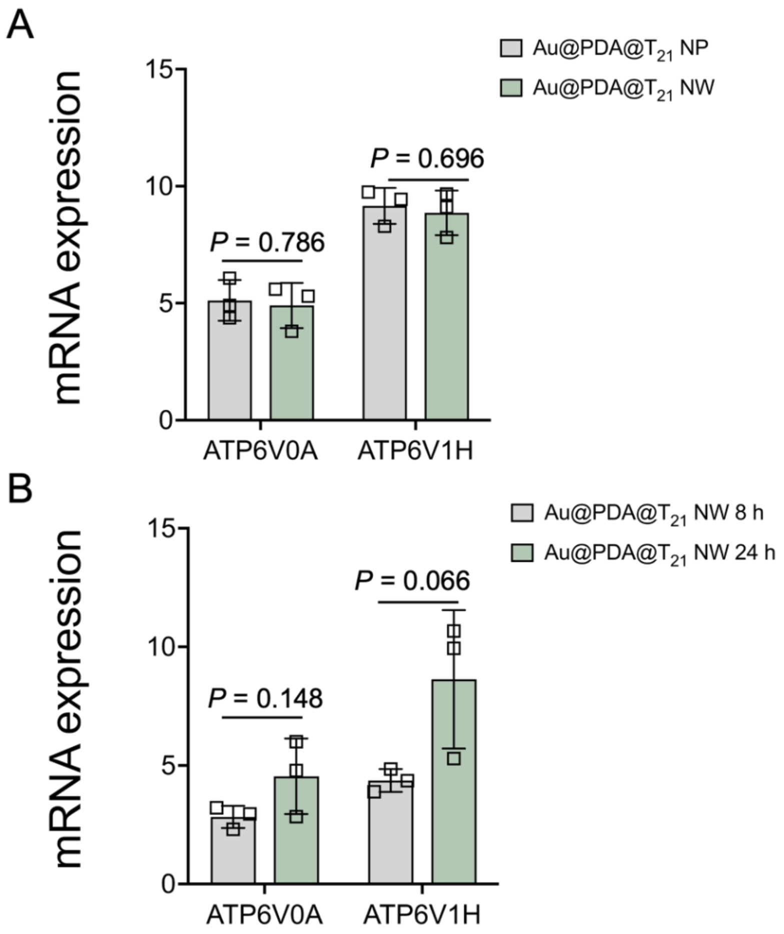

Figure S24. Expression of V-ATPase in A549 cells. A549 cells were incubated with (**A**) Au@PDA@T_{21} NW or Au@PDA@T_{21} NP for 24 h; and (**B**) Au@PDA@T_{21} NW for 8 or 24 h. mRNA expression of two subunits of V-ATPase (ATP6V0A and ATP6V1H) was obtained by RNA-seq. Data are presented as mean ± SEM. Statistical significance was calculated by Student's t-test. n = 3 biological replicates per group, across 1 experiment.

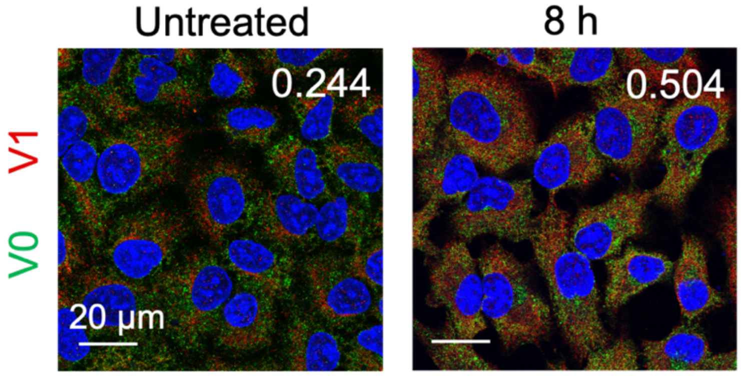

Figure S25. Enhanced activity of V-ATPase 8 h post-incubation with Au@PDA@T_{21} NW. White number indicates PCC between the two subunits of V-ATPase, V1 and V0. Blue = nuclei.

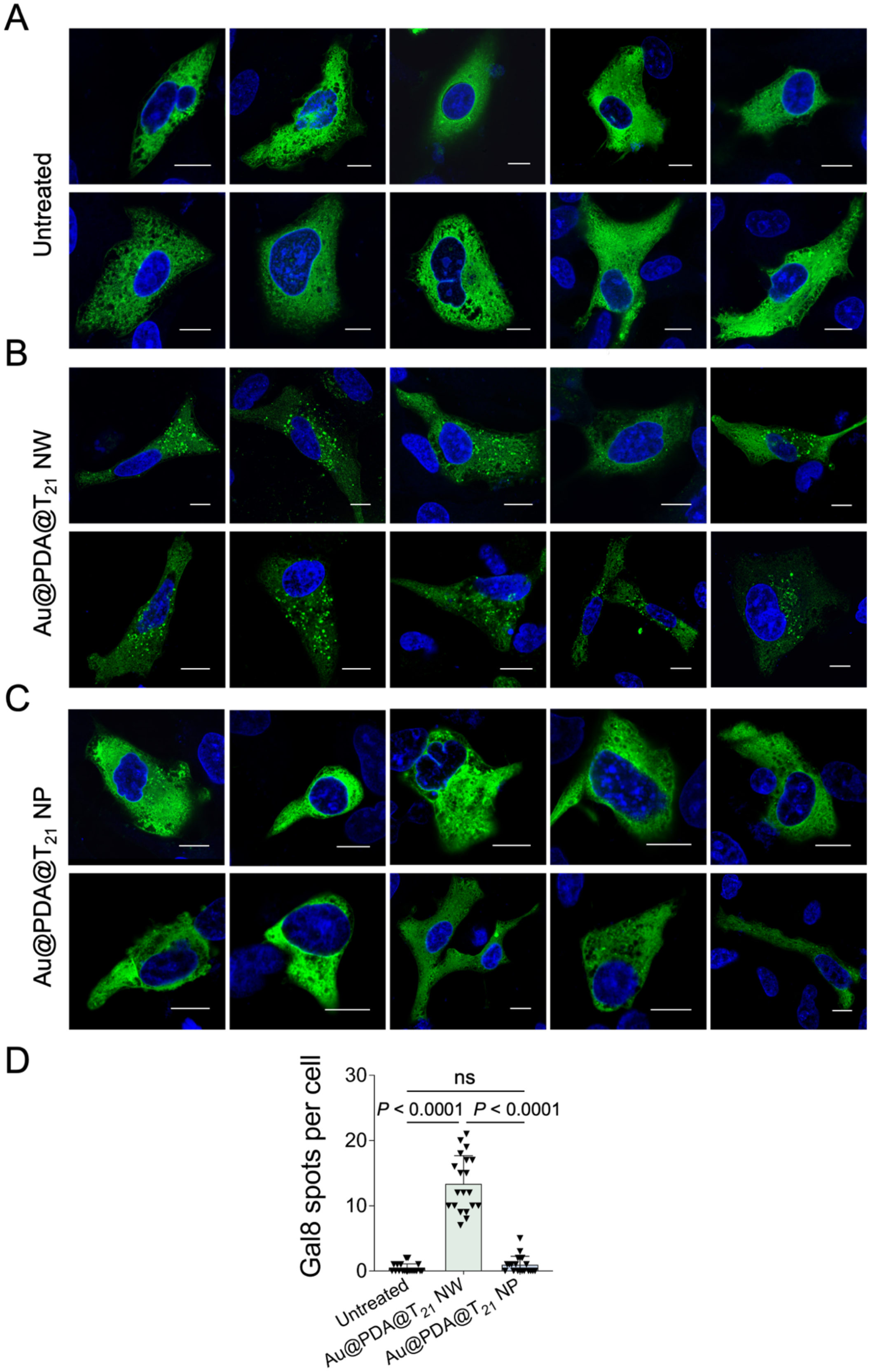
A
Untreated
B
Au@PDA@T21 NW
C
Au@PDA@T21 NP
D
Gal8 spots per cell
ns
P < 0.0001
P < 0.0001
Untreated
Au@PDA@T21 NW
Au@PDA@T21 NP

Figure S26. Endosomal membrane rupture upon Au@PDA@T_{21} NW incubation. Additional representative confocal images of Gal8-GFP transfected A549 cells that were (**A**) untreated, (**B**) treated with Au@PDA@T_{21} NWs for 24 h, and (**C**) treated with Au@PDA@T_{21} NPs for 24 h. Green clusters indicate the recruitment of Gal8-GFP to the ruptured endosomal membrane due to Au@PDA@T_{21} NW treatment. Blue = nuclei. Scale bar = 10 µm. (**D**) Quantification of Gal8-GFP clusters per cell. Data are presented as mean ± SEM. Statistical significance was calculated by one-way ANOVA with Tukey's test for post-hoc analysis. ns: not significant ($P > 0.05$). n = 20 cells per group, across 3 experiments.

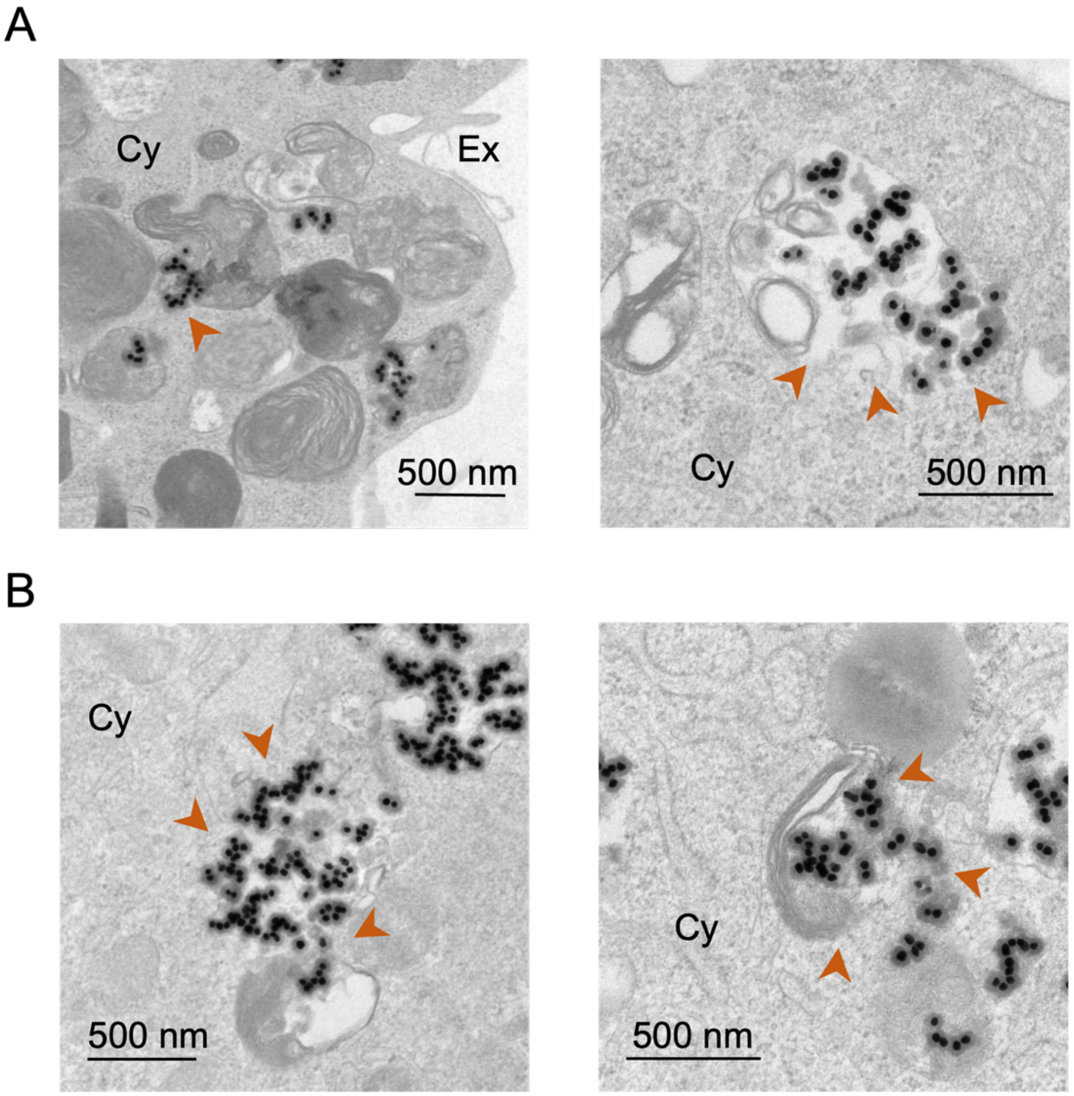

Figure S27. Membrane rupture of intracellular vesicles due to treatement with olignucleotide encased Au@PDA NW. Representative TEM images revealed vesicular membrane rupture in (**A**) A549 cells and (**B**) hMSCs 8 h post-incubation with Au@PDA@T_{21} NW and Au@PDA@siNog NW, respectively. Orange arrows denote the escape of NWs from the ruptured vesicular membrane. Ex = extracelular space. Cy = cytosol.

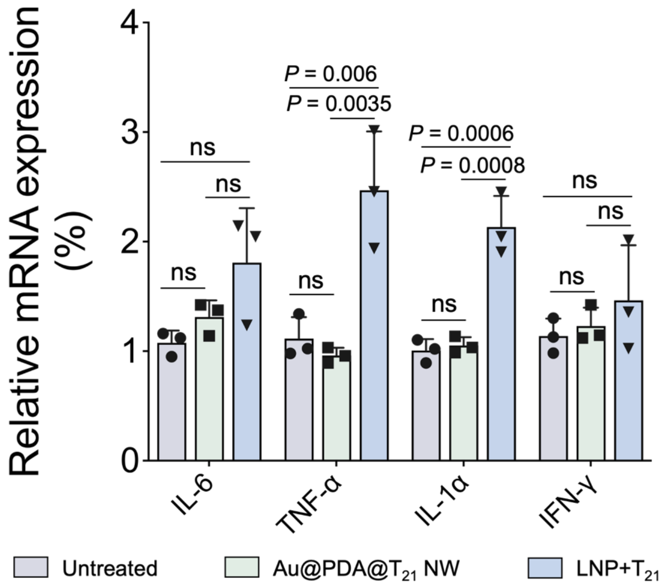

Figure S28. qRT-PCR analysis of inflammatory markers after incubating A549 cells with LNP (formulated with ionizable lipid ALC-0315)+T_{21} and Au@PDA@T_{21} NW for 24 h. Data are presented as mean ± SEM. Statistical significance was calculated by one-way ANOVA with Tukey's Test for post-hoc analysis. ns = not significant ($P > 0.05$). n = 3 biological replicates per group, across 1 experiment.

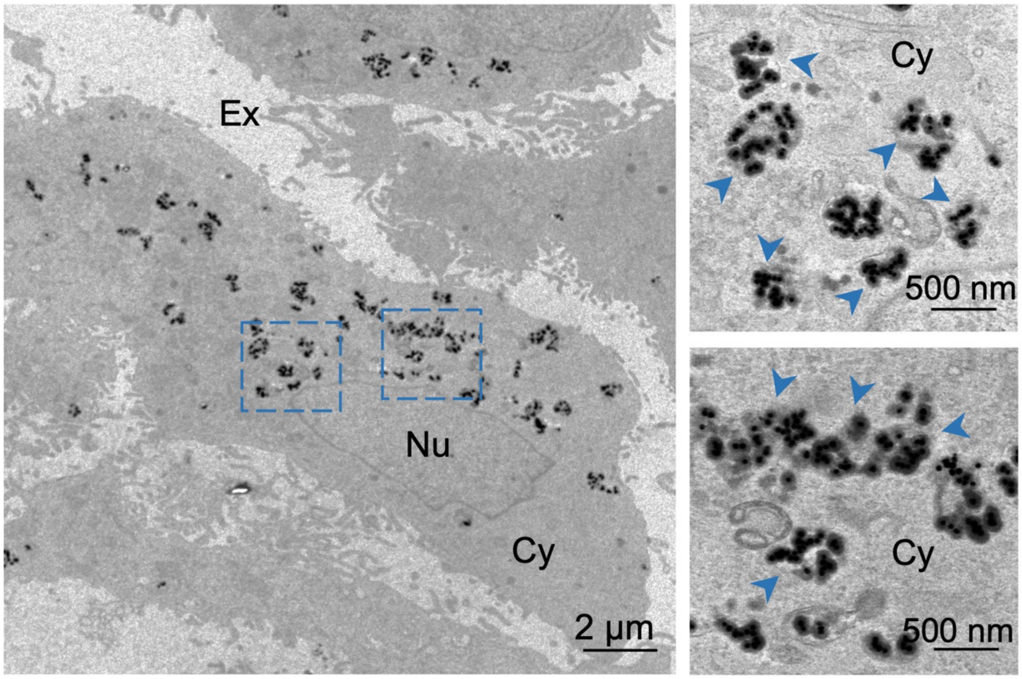

Figure S29. Representative TEM images of bEnd.3 cells show the cytosolic accumulation of Au@PDA@asEGFP NWs (blue arrows) 24 h post-incubation. Nu = nucleus. Cy = cytosol. Ex = extracellular space. The two smaller images on the right show the enlargement of the boxed areas (blue) of the larger image on the left.

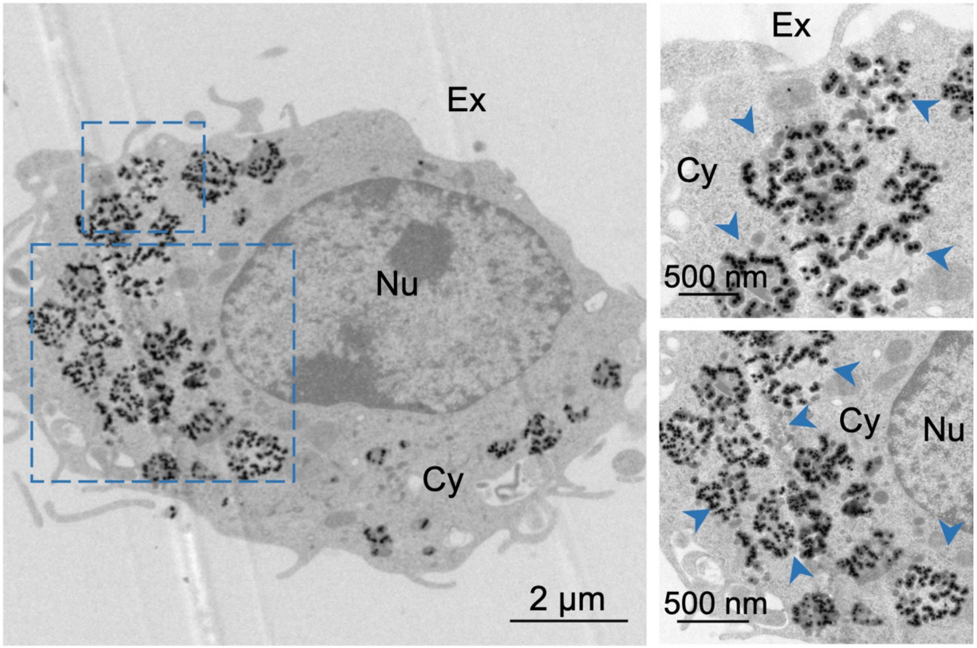

Figure S30. Representative TEM images of BMDMs show cytosolic accumulation of Au@PDA@miR-223 NWs (blue arrows) 24 h post-incubation. Nu = nucleus. Cy = cytosol. Ex = extracellular space. The two smaller images on the right show the enlargement of the boxed areas (blue) of the larger image on the left.

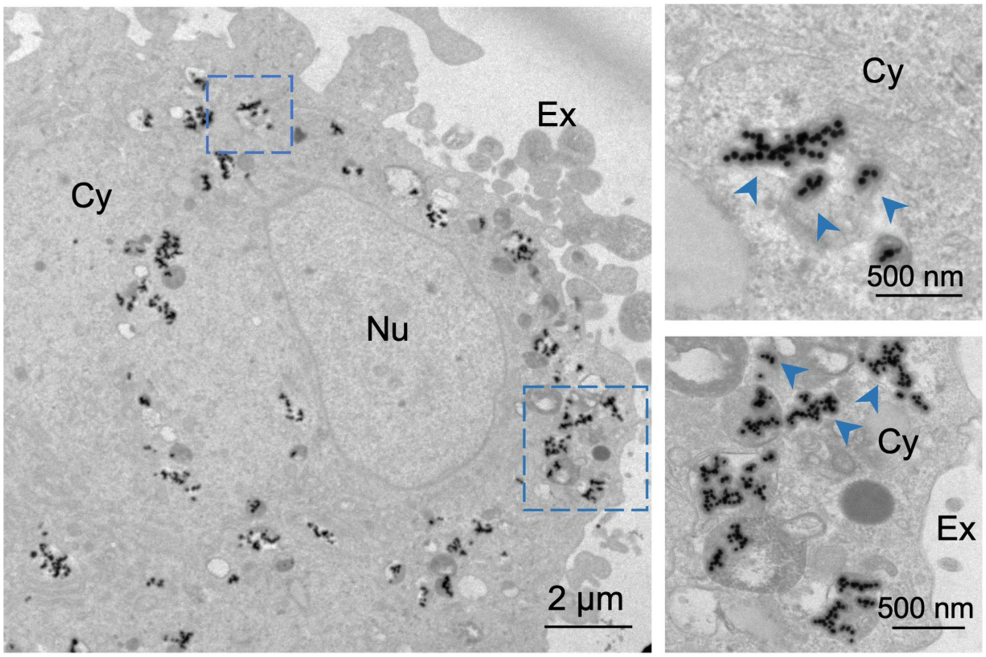

Figure S31. Representative TEM images of hMSCs show cytosolic accumulation of Au@PDA@siNog NWs (blue arrows) 24 h post-incubation. Nu = nucleus. Cy = cytosol. Ex = extracellular space. The two smaller images on the right show the enlargement of the boxed areas (blue) of the larger image on the left.

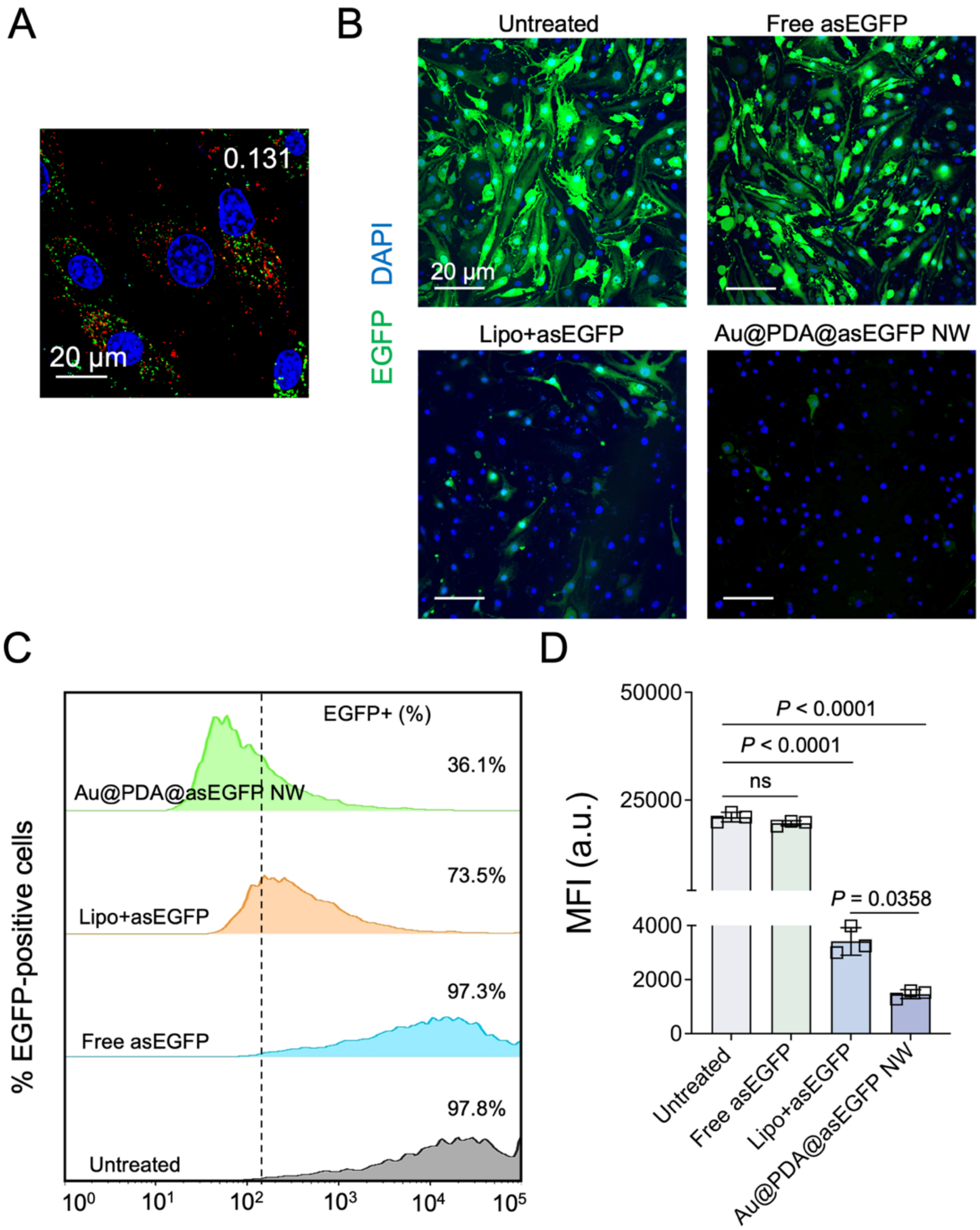

Figure S32. EGFP knockdown by Au@PDA@asEGFP NW. (**A**) Confocal images of EGFP-expressing bEnd.3 cells revealed limited colocalization of Cy5-labeled Au@PDA@asEGFP NW with acidic vesicles 24 h post-incubation. Blue = nuclei. White number indicates PCC between Cy5-labeled Au@PDA@asEGFP NW (red) and LysoTracker (green). (**B**) Confocal images of EGFP-expressing bEnd.3 cells upon various treatments. Green = EGFP; blue = nuclei. (**C**) Percentage of EGFP-positive cells and (**D**) mean fluorescence intensity (MFI) of EGFP measured by flow cytometry. Data are presented as mean ± SEM. Statistical significance was calculated by one-way ANOVA with Tukey's Test for post-hoc analysis. ns: not significant ($P > 0.05$). n = 3 biological replicates per group, across 1 experiment.

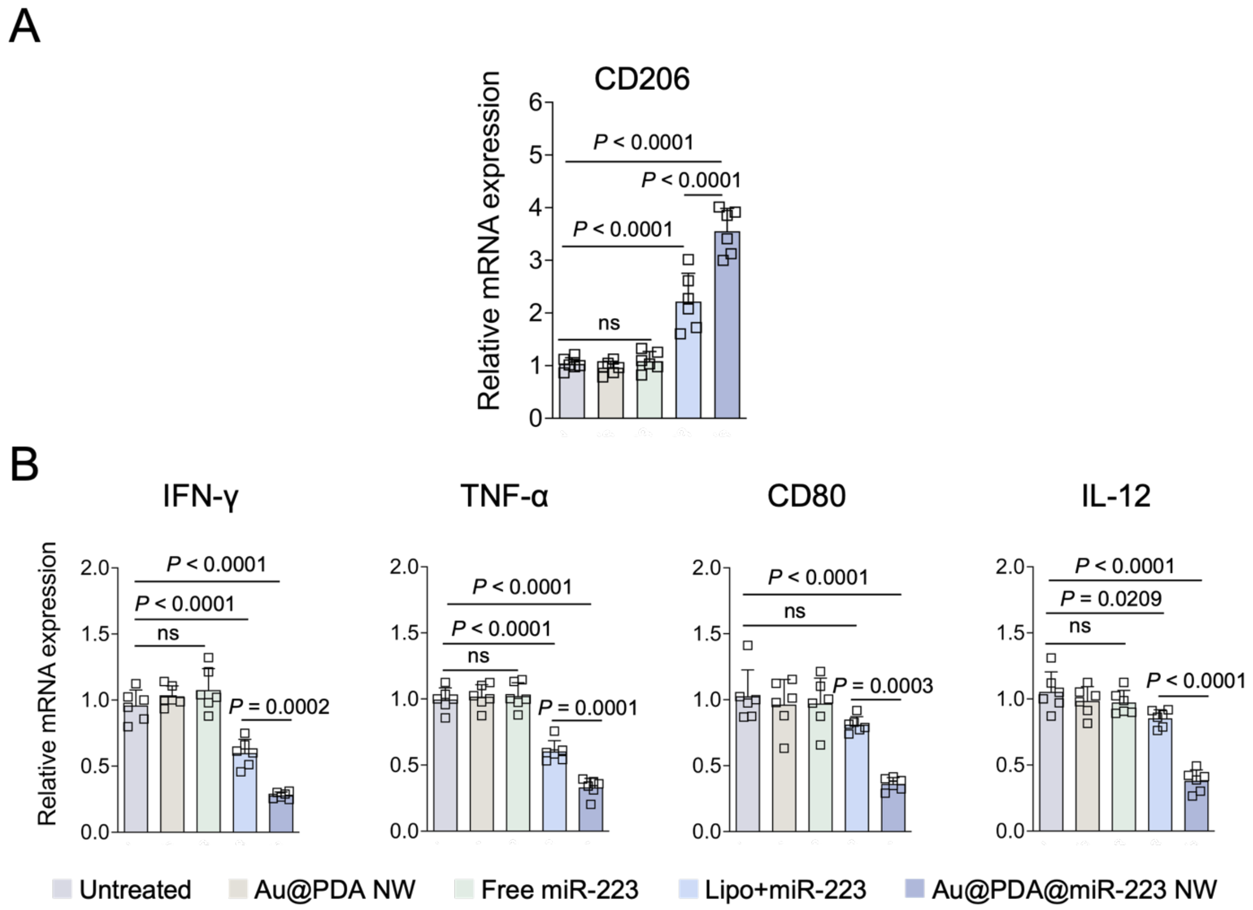

Figure S33. In vitro NW-enabled transfection of miR-223 for inducing repolarization of BMDMs from M1 to M2 phenotype. qRT-PCR measurements of (a) CD206 (M2 phenotype marker) and (b) M1 phenotype markers. Data are presented as mean ± SEM. Statistical significance was calculated by one-way ANOVA with Tukey's Test for post-hoc analysis. ns = not significant ($P > 0.05$). n = 6 per group, across 2 experiments.

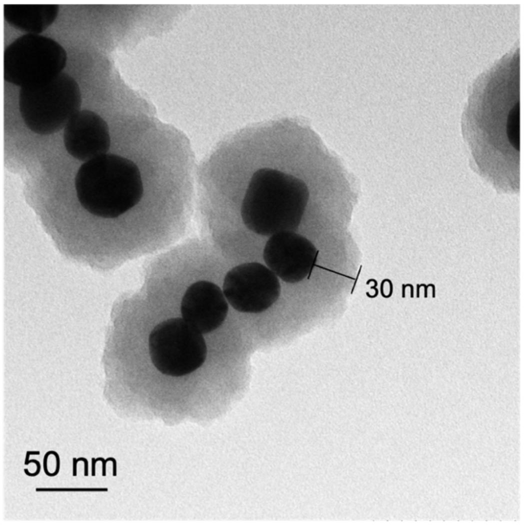

Figure S34. Representative TEM image of a lipid-coated NW (Au@PDA@lipid NW). The thickness of the outer shell increased from ~20 nm (for the PDA shell only of Au@PDA NW) to ~30 nm (for the combined lipid/PDA shell of Au@PDA@lipid NW).

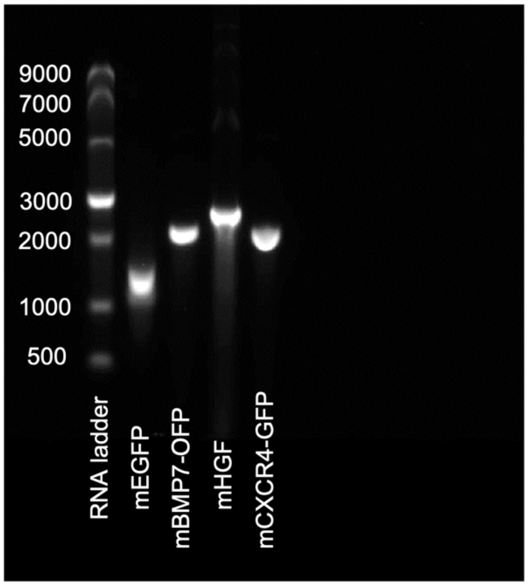

Figure S35. Native agarose gel electrophoresis of mRNA as synthesized by in vitro transcription. From left to right, the samples are RNA ladder (500–9000 nt), mEGFP, mBMP7-OFP, mHGF, and mCXCR4-GFP. The expected sizes of mEGFP, mBMP7-OFP, mHGF, and mCXCR4-GFP are ~1100 nt, 2200 nt, 2500 nt, and 2100 nt, respectively.

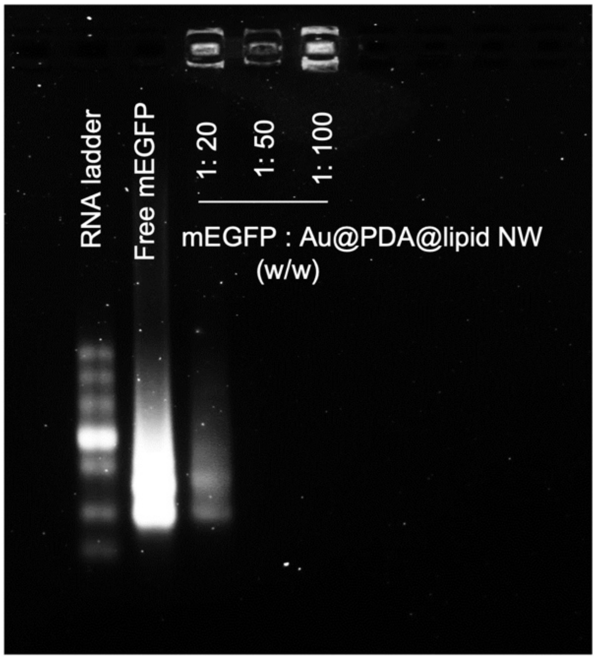

Figure S36. Gel retardation analysis of mRNA loading on Au@PDA@lipid NW. mRNA encoding EGFP (mEGFP) was adsorbed to the surface of Au@PDA@lipid NW at varying weight ratios (mEGFP: Au@PDA@lipid NW) from 1:20 to 1:100. From left to right, the samples are RNA ladder (500–9000 nt), free mEGFP, mEGFP: Au@PDA@lipid NW = 1:20 (w/w), 1:50 (w/w), and 1:100 (w/w). The mRNA loading was saturated at the weight ratio of 1:50, as evidenced by the disappearance of free mEGFP running down the lane.

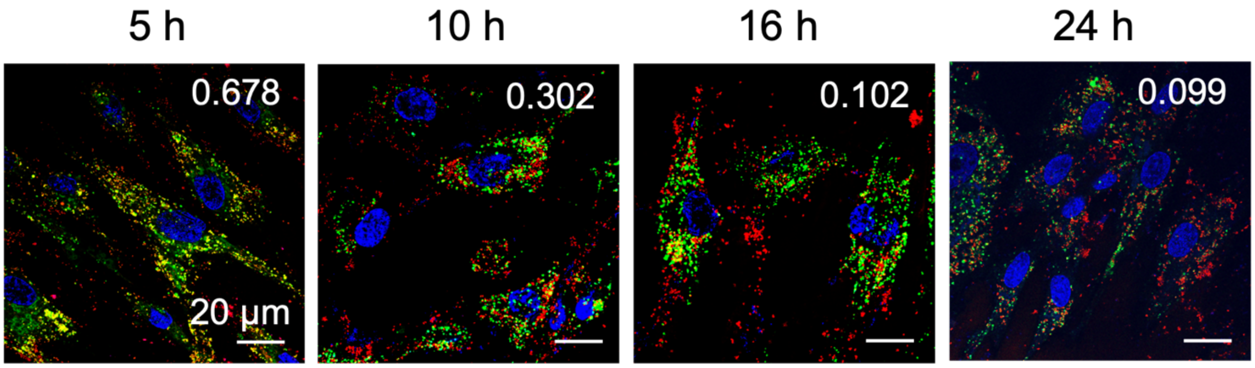

Figure S37. Intracellular trafficking of Au@PDA@mEGFP NW in hMCSs. Time-lapse confocal images showed that Cy5-labeled Au@PDA@mEGFP NWs were colocalized with acidic organelles (stained with LysoTracker) 5 h post-incubation and escaped from those vesicles 10 h post-incubation. Blue = Nuclei. White number indicates PCC between Cy5-labled Au@PDA@mEGFP NWs (red) and LysoTracker (green).

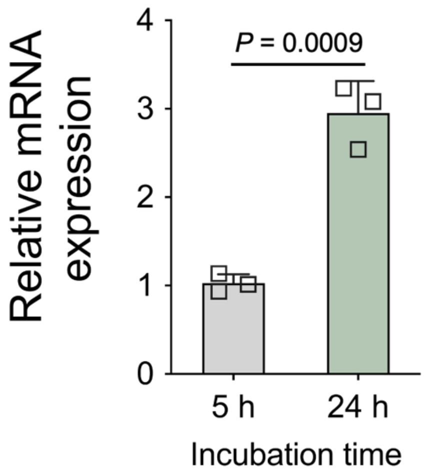

Figure S38. qPCR analysis of ClC3 expression in hMSCs after incubation of Au@PDA@mEGFP NW. hMSCs were incubated with Au@PDA@mEGFP NWs for 5 h (when the NWs were strongly colocalized with acidic vesicles) and 24 h (when most NWs escaped from acidic vesicles). ClC3 expression was higher at 24 h post-incubation than at 5 h post-incubation, consistent with the upregulation of ClC3 in A549 cells upon treatment with Au@PDA@T_{21} NWs. Data are presented as mean ± SEM. Statistical significance was calculated by Student's t-test. n = 3 biological replicates per group, across 1 experiment.

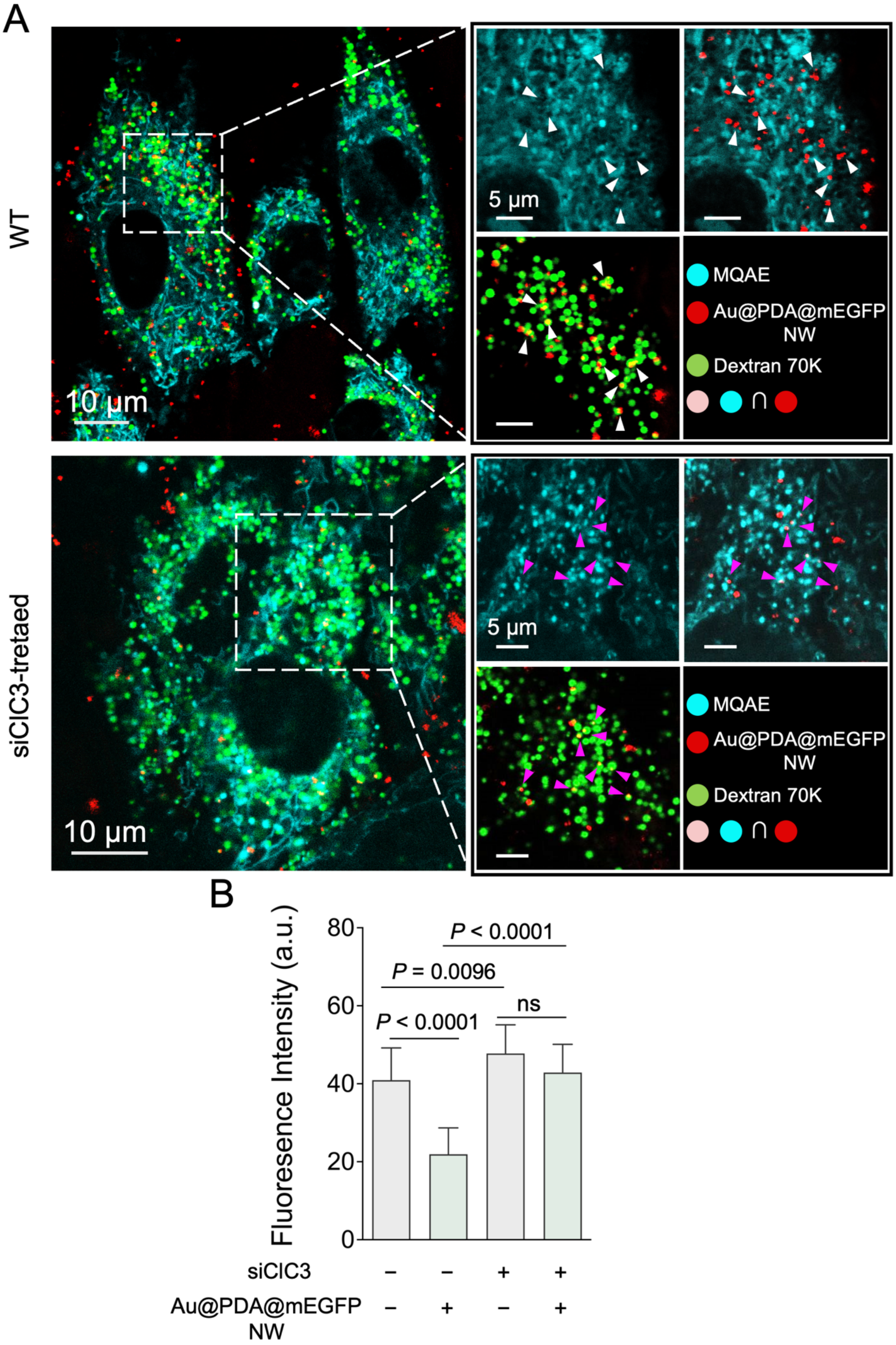
A
WT
siClC3-tretaed
10 μm
5 μm
MQAE
Au@PDA@mEGFP NW
Dextran 70K
∩
B
Fluoresence Intensity (a.u.)
80
60
40
20
0
P < 0.0001
P = 0.0096
P < 0.0001
ns
siClC3
Au@PDA@mEGFP NW
–
+

Figure S39. pH measurement of intracellular vesicles in hMSCs. (**A**) pH measurement of intracellular vesicles without (upper row) or with siClC3 (lower row) 5 h post co-incubation with Au@PDA@mEGFP NW (magenta), dextran 10K pHrodo (a pH-sensitive dye; green), and dextran 10K AF594 (a pH-insensitive dye; red). H^+ concentration in NW-containing vesicles (magenta arrows) is calculated by the red to green (R/G) intensity ratio; R/G ratio is inversely proportional to H^+ concentration. Scale bar = 10 μm. (**B**) Activity of V-ATPase of hMSCs upon incubation with Au@PDA@mEGFP NW: The activity of V-ATPase increased 5 h post-incubation of Au@PDA@mEGFP NWs. Blue = nuclei. White number indicates PCC between V0 (green) and V1 (red). (**C**) Quantification of the pH value of individual vesicles in wildtype and siClC3-treated hMSC by measuring R/G ratio. Data are presented as mean ± SEM. Statistical significance was calculated by one-way ANOVA with Tukey's Test for post-hoc analysis. ns = not significant ($P > 0.05$). n = 300 intracellular vesicles per group.

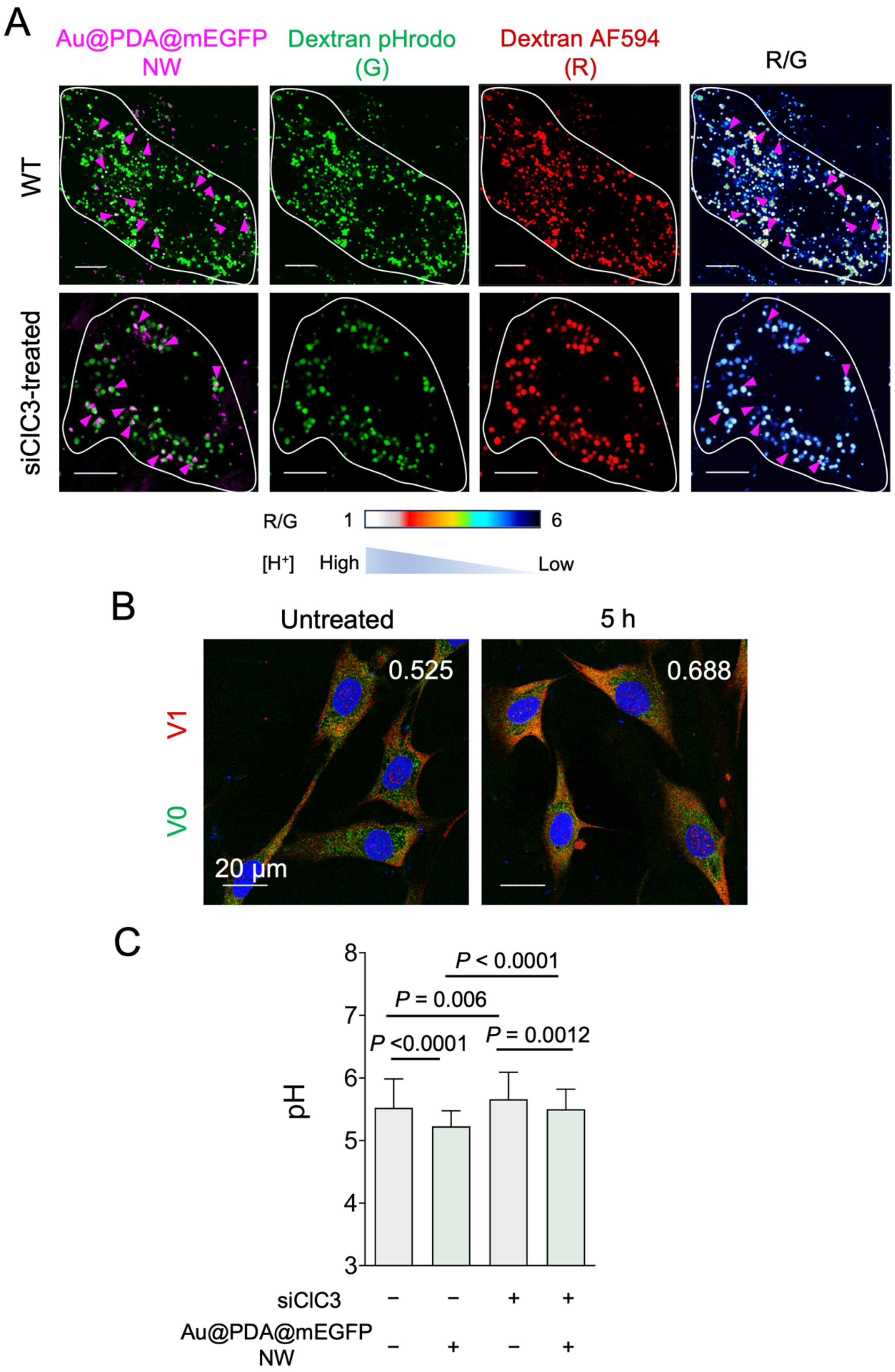
A
Au@PDA@mEGFP NW
Dextran pHrodo (G)
Dextran AF594 (R)
R/G
WT
siClC3-treated
R/G 1 6
[H+] High Low
B
Untreated
5 h
0.525
0.688
V1
V0
20 µm
C
P < 0.0001
P = 0.006
P <0.0001
P = 0.0012
pH
siClC3
Au@PDA@mEGFP NW

Figure S40. pH measurement of intracellular vesicles in hMSCs. (**A**) pH measurement of intracellular vesicles without (upper row) or with siClC3 (lower row) 5 h post co-incubation with Au@PDA@mEGFP NW (magenta), dextran 10K pHrodo (a pH-sensitive dye; green), and dextran 10K AF594 (a pH-insensitive dye; red). H^+ concentration in NW-containing vesicles (magenta arrows) is calculated by the red to green (R/G) intensity ratio; R/G ratio is inversely proportional to H^+ concentration. Scale bar = 10 µm. (**B**) Activity of V-ATPase of hMSCs upon incubation with Au@PDA@mEGFP NW as a function of time. The activity of V-ATPase increased 5 h post-incubation of Au@PDA@mEGFP NWs. Blue = nuclei. White number indicates PCC between V0 (green) and V1 (red). (**C**) Quantification of the pH value of individual vesicles in wildtype and siClC3-treated hMSC by measuring R/G ratio. Data are presented as means ± SEM. Statistical significance was calculated by one-way ANOVA with Tukey's Test for post-hoc analysis. ns = not significant ($P > 0.05$). n = 300 intracellular vesicles per group.

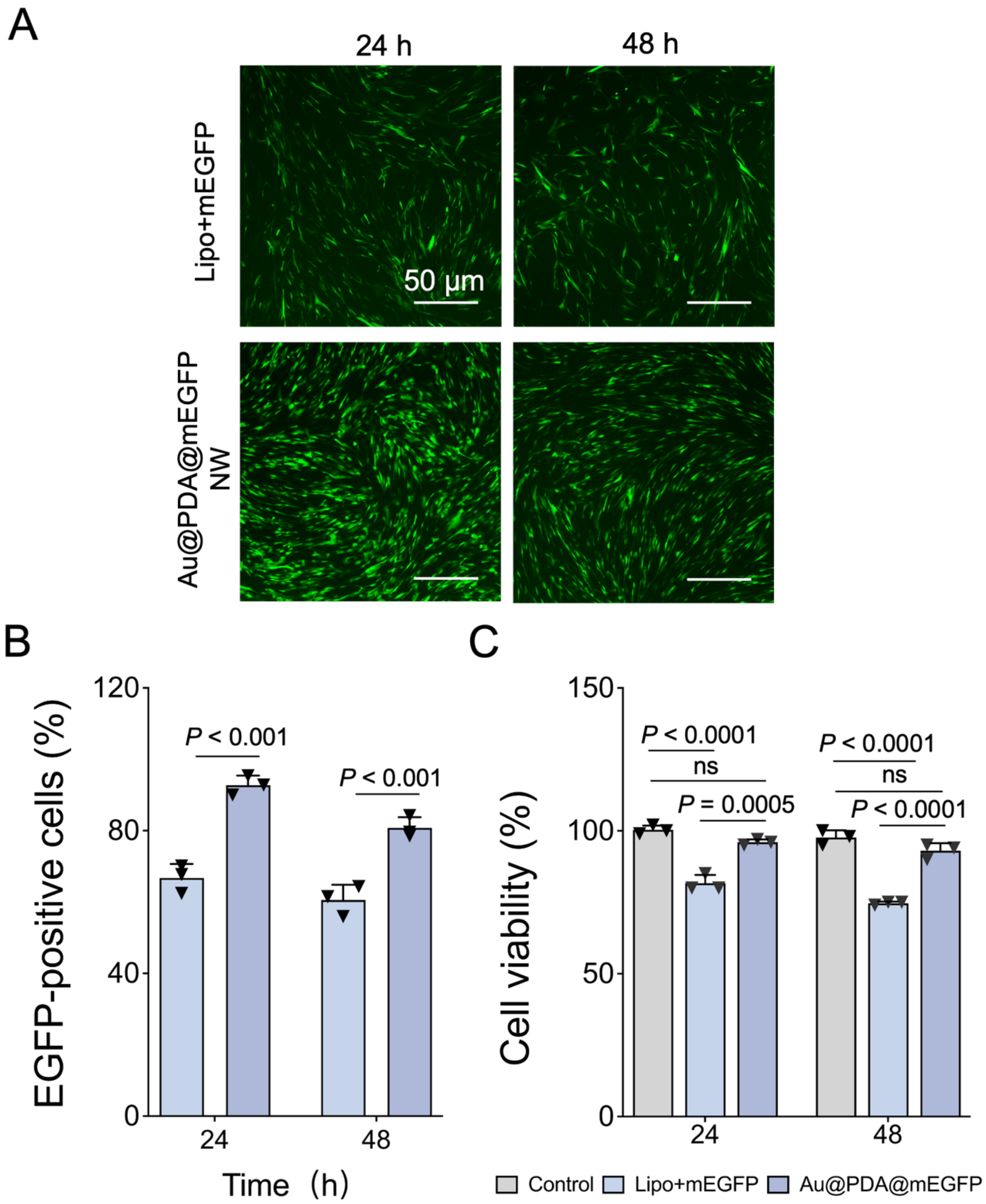

Figure S41. In vitro Au@PDA@lipid NW-enabled transfection of mEGFP in hMSCs. (**A**) Representative fluorescence images confirmed the expression of EGFP (green) due to transfection by Lipofectamine 3000 and Au@PDA@mEGFP NW 24 h and 48 h post-incubation. (**B**) Transfection efficiency of mEGFP (as measured by flow cytometry) and (**C**) cell viability 24 and 48 h post-incubation. Data are presented as mean ± SEM. Statistical significance was calculated by one-way ANOVA with Tukey's Test for post-hoc analysis. ns = not significant (P > 0.05). n = 3 biological replicates per group, across 1 experiment.

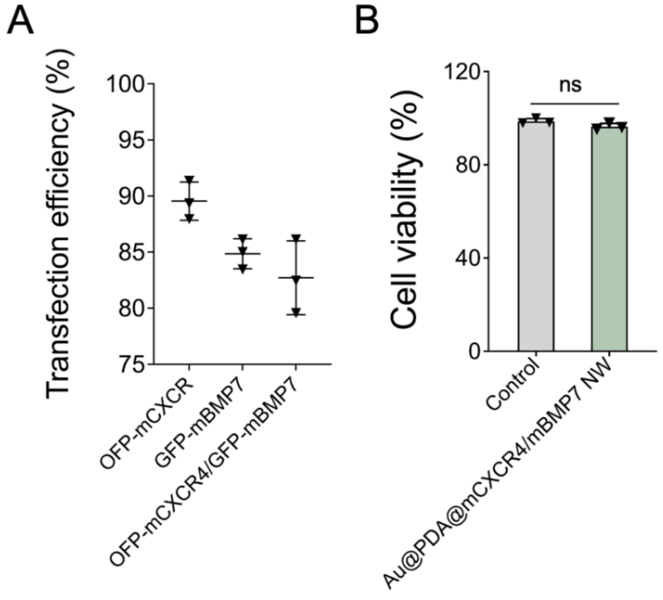

Figure S42. In vitro Au@PDA@lipid NW-enabled transfection of mCXCR4 and mBMP7 in hMSCs. (**A**) Transfection efficiency of mCXCR4-GFP and mBMP7-OFP of hMSCs was evaluated by flow cytometry 24 h post-transfection. The amounts of mCXCR4-GFP and mBMP7-OFP added to the hMSCs were kept the same. (**B**) Cell viability 24 h post-incubation of Au@PDA@mCXCR4/mBMP7 NW. Data are presented as mean ± SEM. Statistical significance was calculated by Student's t-test. ns = not significant ($P > 0.05$). n = 3 biological replicates per group, across 1 experiment.

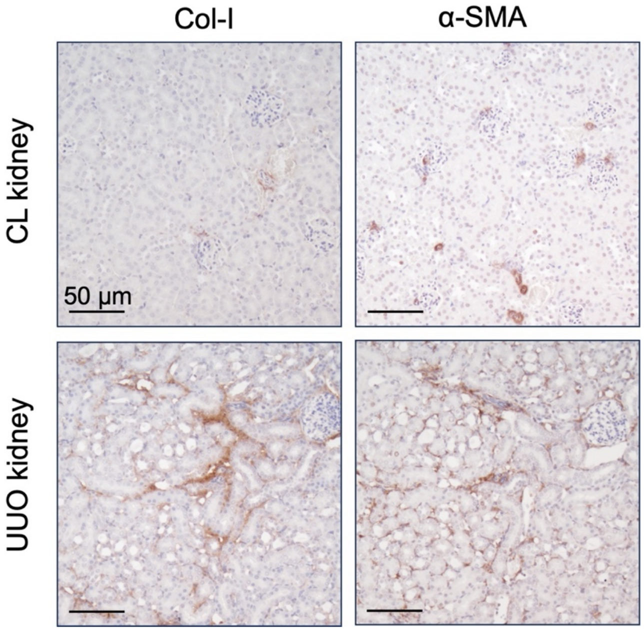

Figure S43. Establishment of renal fibrosis. Kidneys were harvested from UUO mice 3 days post-surgery and stained by IHC to confirm the enhanced expression of Col-I and α-SMA in the UUO kidney, not the contralateral kidney (CL kidney).

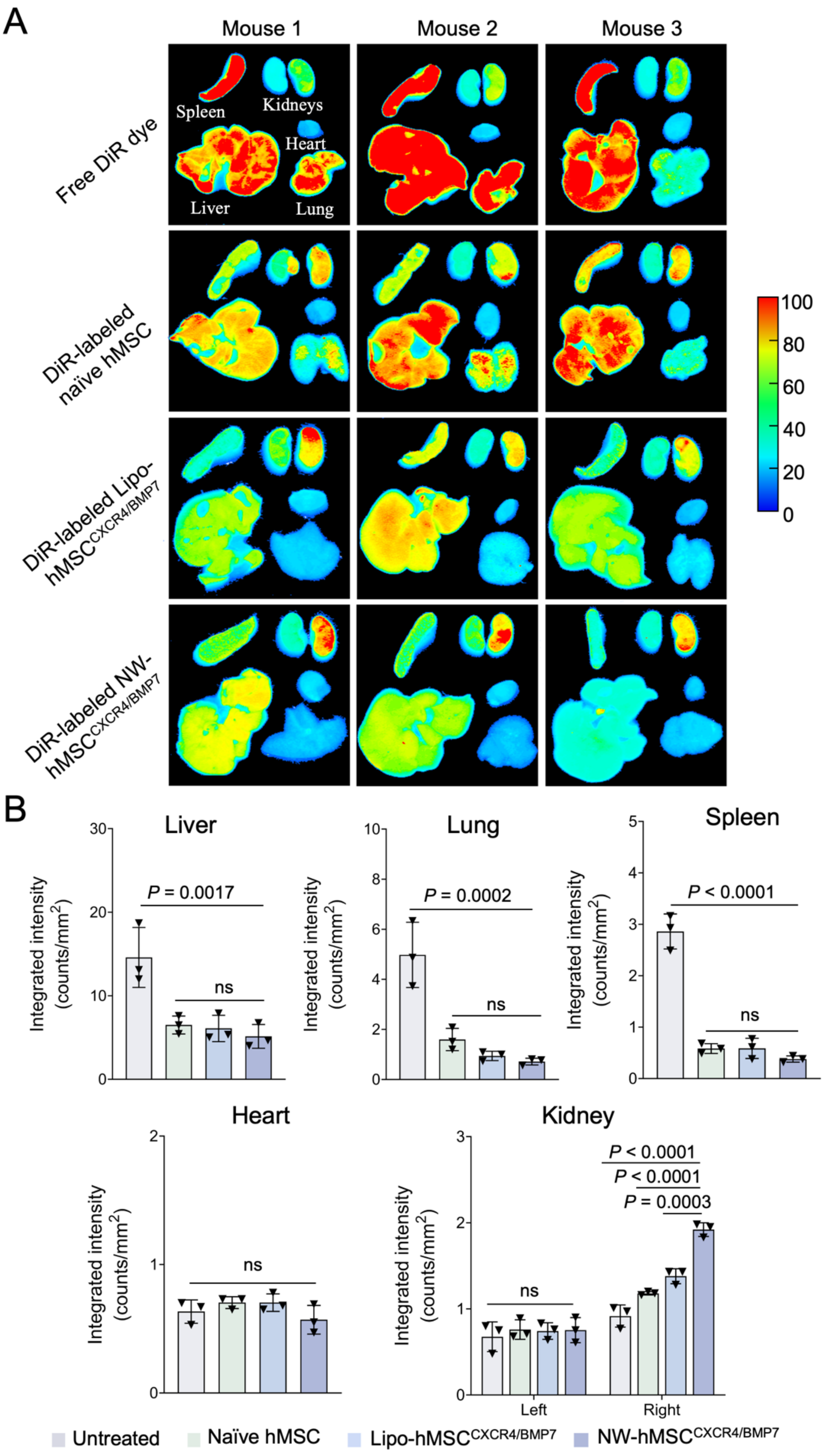

A
Mouse 1
Mouse 2
Mouse 3
Free DiR dye
Spleen
Kidneys
Heart
Liver
Lung
DiR-labeled naïve hMSC
DiR-labeled Lipo-hMSC$^{CXCR4/BMP7}$
DiR-labeled NW-hMSC$^{CXCR4/BMP7}$
100
80
60
40
20
0
B
Liver
Lung
Spleen
Heart
Kidney
Integrated intensity (counts/mm^2)
P = 0.0017
P = 0.0002
P < 0.0001
P < 0.0001
P < 0.0001
P = 0.0003
ns
Left
Right
Untreated
Naïve hMSC
Lipo-hMSC$^{CXCR4/BMP7}$
NW-hMSC$^{CXCR4/BMP7}$

Figure S44. Accumulation of injected hMSCs in major organs. (**A**) Representative ex vivo near-infrared fluorescence (NIRF) imaging 24 h post-single i.v. injection of DiR-labeled hMSCs in UUO mice. The stronger DiR fluorescence of UUO kidney from the Au@PDA@mCXCR4/mBMP7 NW-transfected hMSC (NW-hMSC$^{CXCR4/BMP7}$) than naïve hMSC and the Lipofectamine-transfected hMSC (Lipo-hMSC$^{CXCR4/BMP7}$) suggests enhanced homing of hMSC to the UUO kidney due to the robust transfection of mCXCR4 mediated by Au@PDA@mCXCR4/mBMP7 NW. (**B**) Quantification of NIRF fluorescence of different organs. Data are presented as mean ± SEM. Statistical significance was calculated by one-way ANOVA with Tukey's Test for post-hoc analysis. ns = not significant ($P > 0.05$). n = 3 biological replicates per group, across 1 experiment.

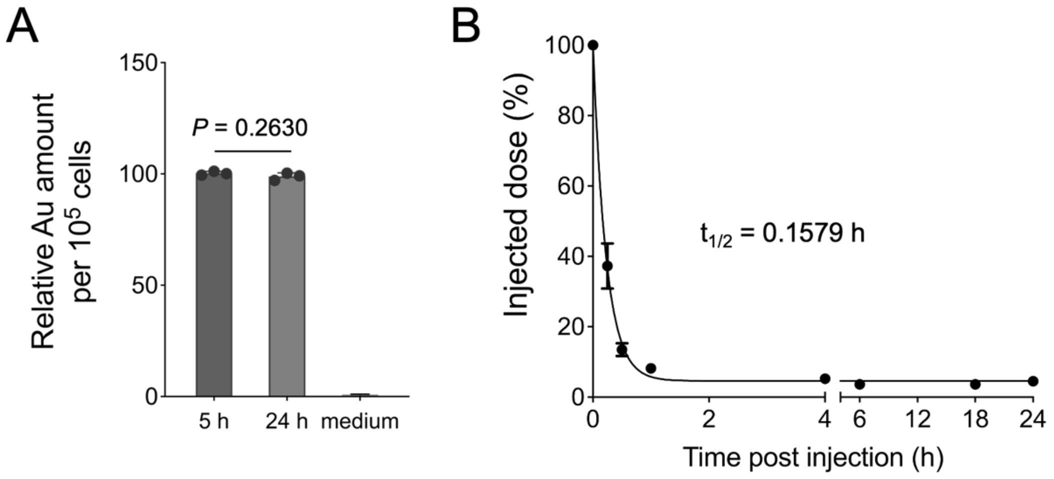

Figure S45. Pharmacokinetics of hMSCs in UUO mice. (**A**) hMSCs were incubated with Au@PDA@mCXCR4/mBMP7 NWs for 5 h, and the NW-containing medium was replaced with fresh medium. The Au content in hMSCs at 24 h post-incubation was similar to that at 5 h post-incubation, and there was little Au content detected in the culture medium by ICP-MS measurements. Therefore, Au@PDA@mCXCR4/mBMP7 NWs were not readily exocytosed by hMSCs. Data are presented as mean ± SEM. Statistical significance was calculated by one-way ANOVA with Tukey's Test for post-hoc analysis. n = 3 biological replicates per group, across 1 experiment. (**B**) Half-life time of hMSCs in blood. The hMSCs were incubated with Au@PDA@mCXCR4/mBMP7 NWs for 24 h before i.v. injection to UUO mice. The gold content in the blood drawn at different time points reflects the number of injected hMSCs circulating in the blood. n = 3 biological replicates per group, across 1 experiment.

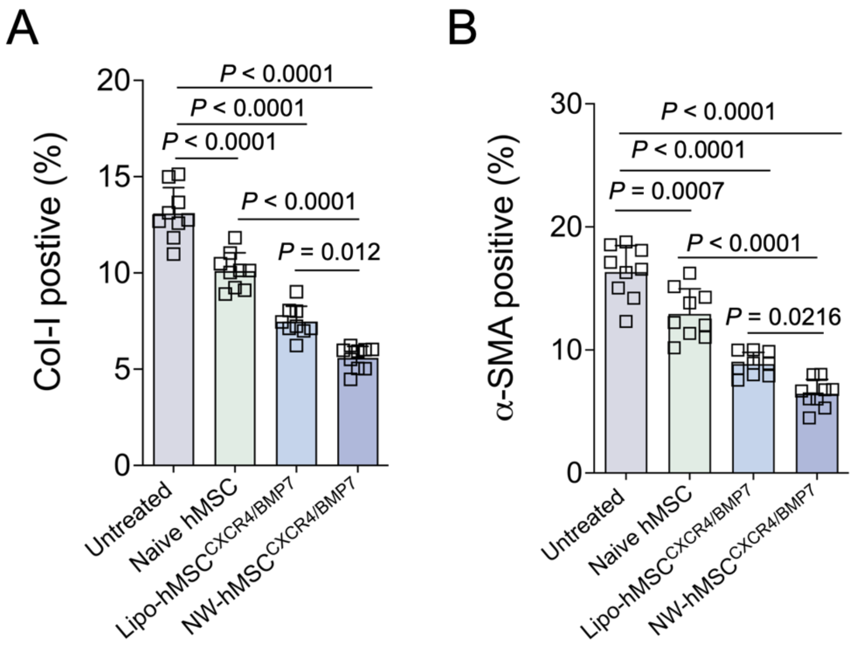

Figure S46. Quantification of (A) Col-I-positive and (B) α-SMA-positive area in the UUO kidney sections by IHC staining following hMSC-based therapy arisen from different treatment groups. Data are presented as mean ± SEM. Statistical significance was calculated by one-way ANOVA with Tukey's Test for post-hoc analysis. n = 8 mice per group (an average of 6 images per mouse), across 2 experiments.

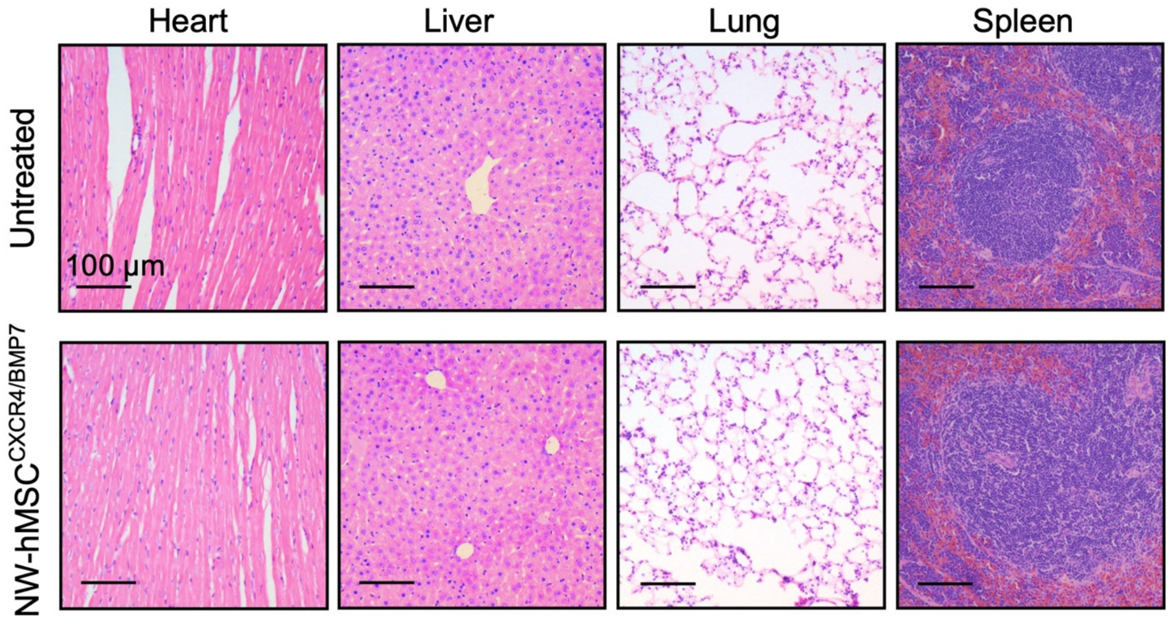

Figure S47. In vivo toxicity of NW-transfected hMSCs in UUO mice. Histological examination of key organs showed that Au@PDA@mCXCR4/mBMP7 NW-transfected hMSC (NW-hMSC$^{mCXCR4/BMP7}$) led to no significant change in tissue morphology 11 d post-injection (or 14 d post-UUO surgery). Representative images were from n = 3 mice per group, across 1 experiment.

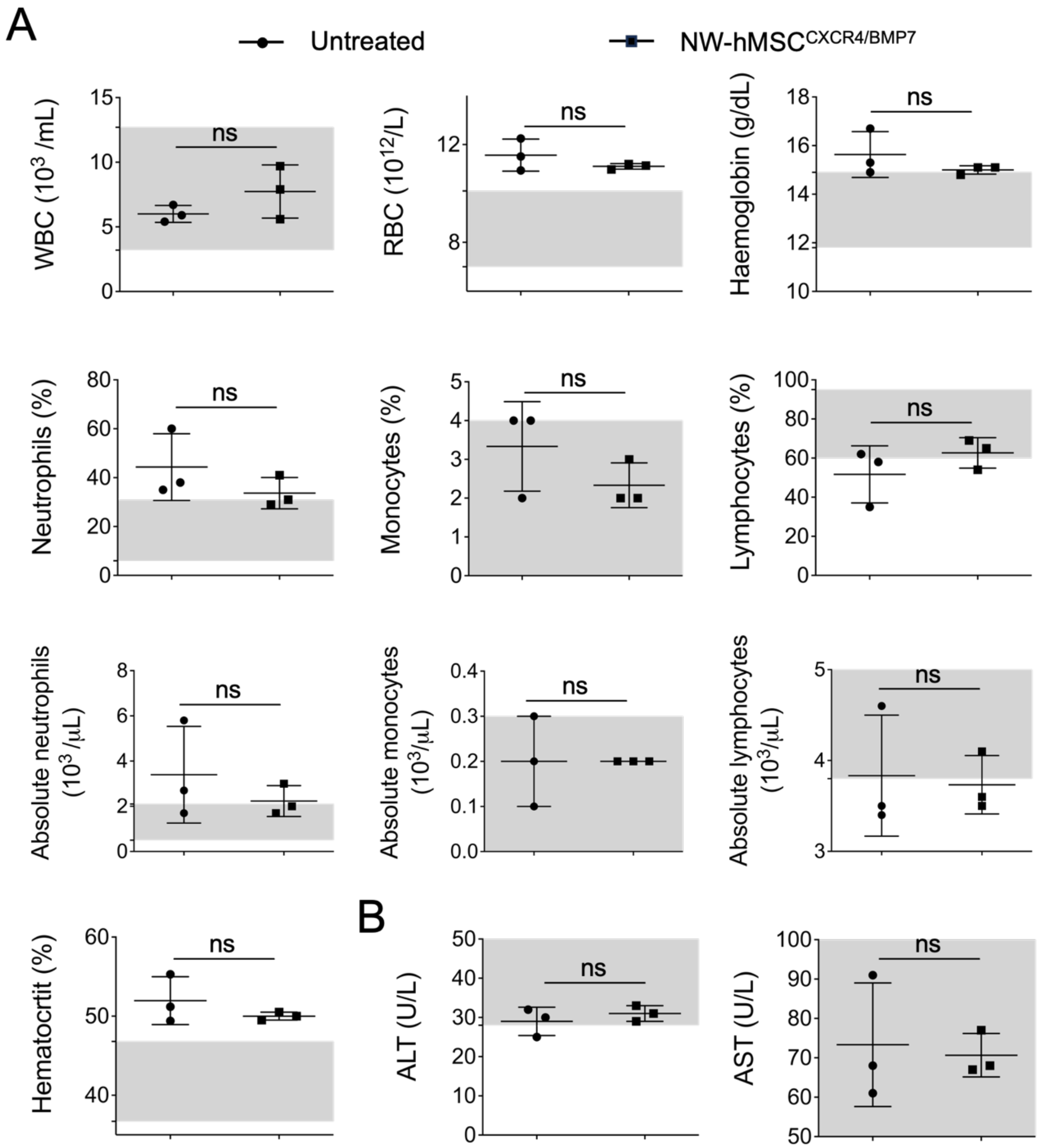

Figure S48. In vivo toxicity of NW-transfected hMSCs in UUO mice 11 d post-i.v. injection (or 14 d post-UUO surgery). Injection of Au@PDA@mCXCR4/mBMP7 NW-transfected hMSC (NW-hMSC$^{mCXCR4/BMP7}$) did not change (**A**) blood chemistry, cell count, and (**B**) liver function. Grey shaded area represents the normal range of markers or cell counts for mice. WBC: white blood cell. RBC: red blood cell. Absolute neutrophils, lymphocytes, or monocytes (10^3/μL): cell number per volume of blood. Neutrophils%, lymphocytes%, monocytes%: percentage of cells out of total WBC. RBC (10^6/μL): cell number per volume of blood. AST: aspartate aminotransferase. ALT: alanine transaminase. Data are presented as mean ± SEM. Statistical significance was calculated by Student's t-test. n = 3 mice per group, across 1 experiment.

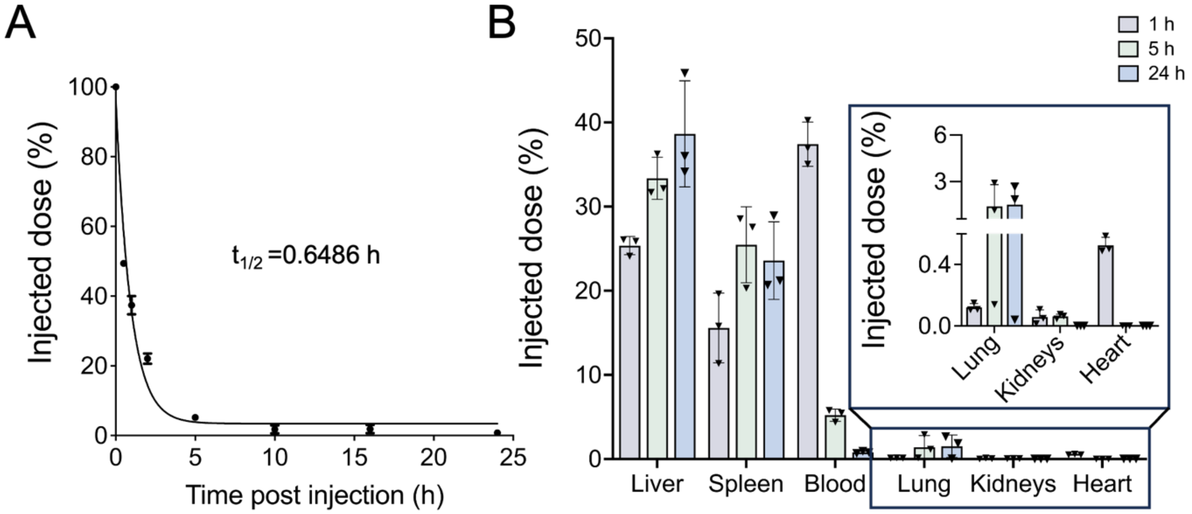

Figure S49. In vivo distribution of Au@PDA@mHGF NWs in ALI mice. (**A**) Blood pharmacokinetics of Au@PDA@mHGF NW. Gold contents were measured by ICP-MS. Data are presented as mean ± SEM. n = 3 per group, across 1 experiment. (**B**) Organ-level distribution data showed that Au@PDA@mHGF NWs accumulated in the liver and spleen 24 h post-injection. Data are presented as mean ± SEM. n = 3 mice per group, across 1 experiment.

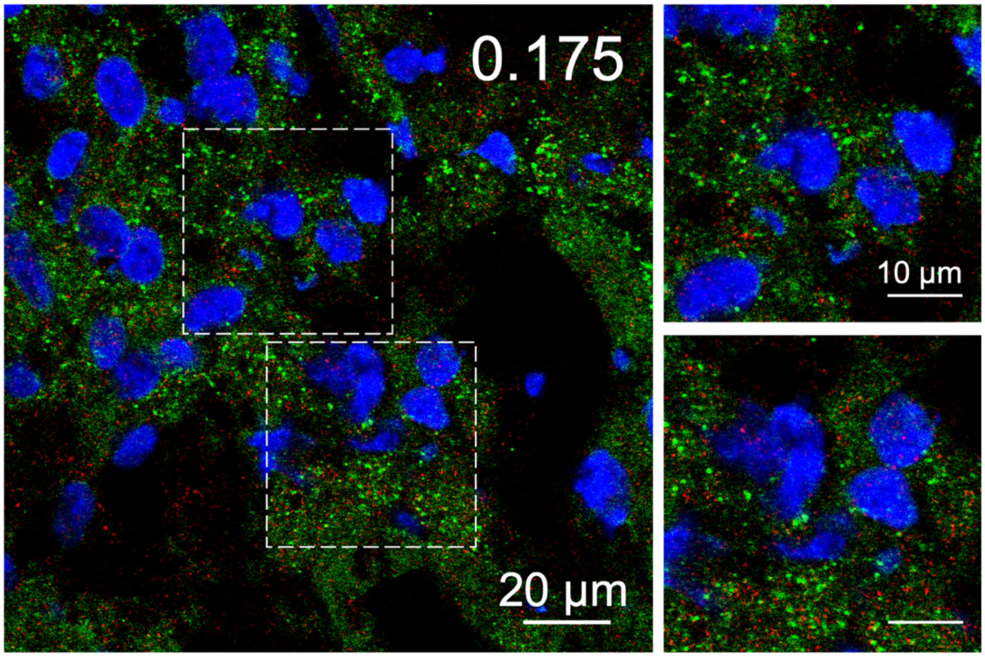

Figure S50. Additional confocal immunofluorescence images of the in vivo endosomal escape of Au@PDA@mHGF NW in the liver 24 h post-injection into ALI mice. These confocal images were taken from different liver sections than those shown in Fig. 6C of the main text. White number indicates PCC between Cy5-labeled mHGF (red) and lysosomes (labeled by the anti-LAMP1 antibody). Blue = nuclei.

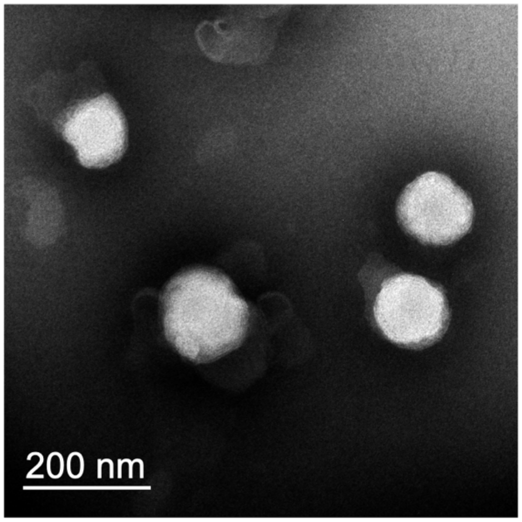

Figure S51. Representative TEM image of mHGF-encapsulated conventional liposomes. By manually counting ~500 liposomes (negatively stained by 1% phosphotungstic acid) across different TEM images, the average physical diameter of these liposomes is 141.3 ± 1.4 nm.

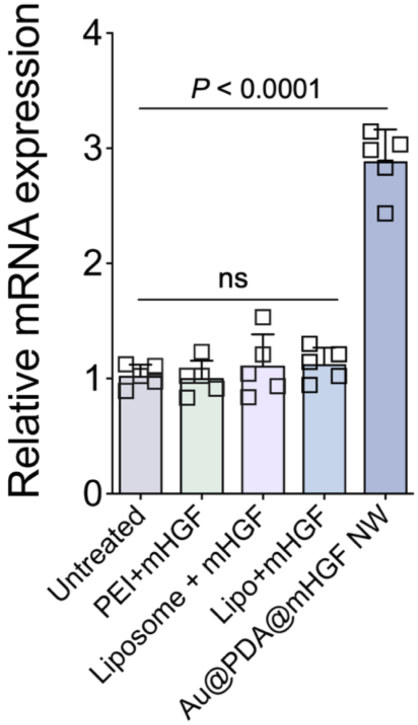

Figure S52. Au@PDA@mHGF NW upregulated ClC3 expression in the ALI liver. qRT-PCR validated the upregulation of ClC3 in the liver of ALI mice that were i.v. injected with Au@PDA@mHGF NW, not other delivery systems, 24 h post-injection. Data are presented as mean ± SEM. Statistical significance was calculated by one-way ANOVA with Tukey's Test for post-hoc analysis. n = 5 biological replicates per group, across 1 experiment.

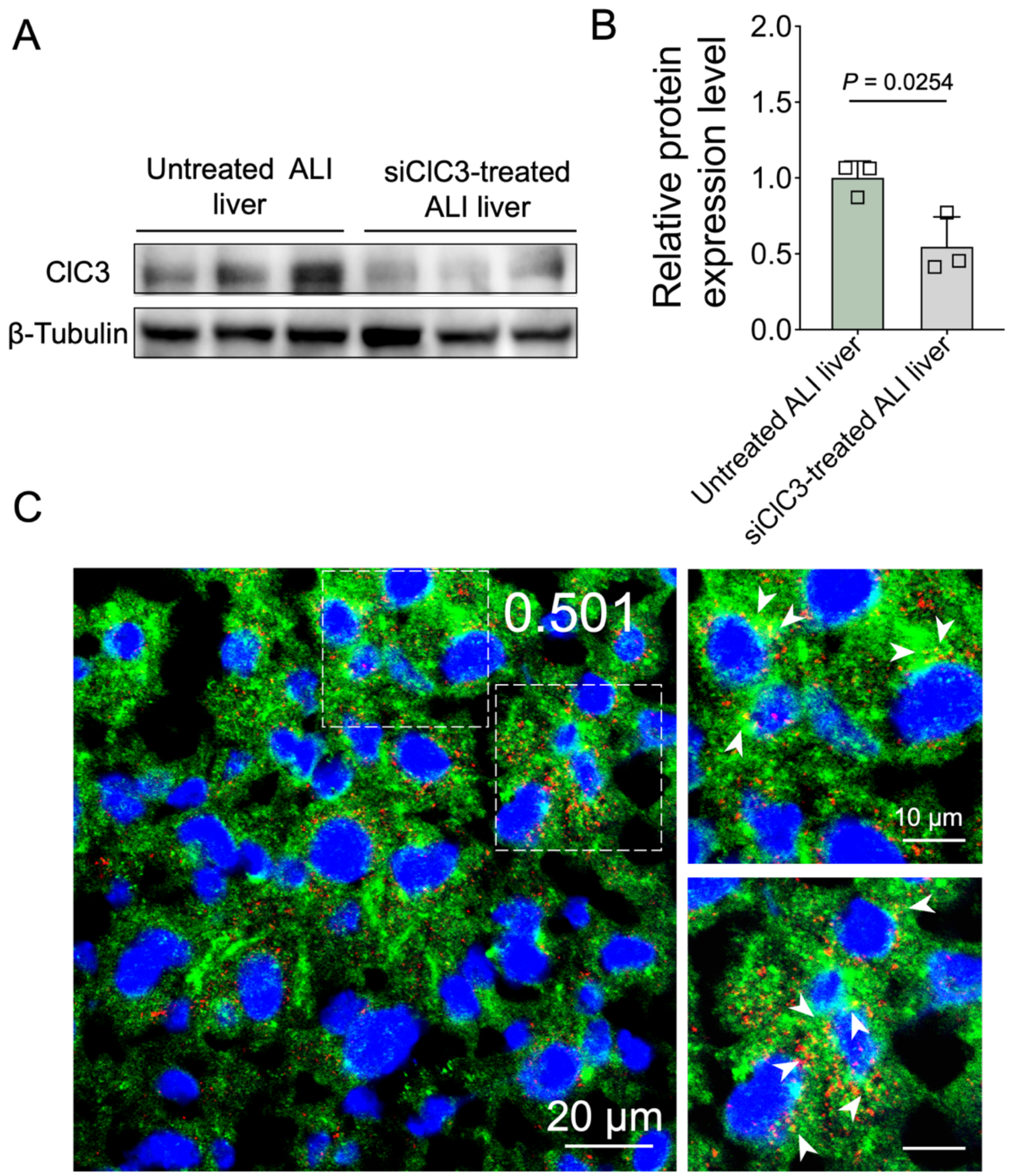

Figure S53. Endosomal escape efficiency of Au@PDA@mHGF NW in the liver of ALI mice injected with Lipo+siClC3. (**A**) Western blot confirmed the in vivo genetic knockdown of ClC3 in the ALI liver 48 h upon a single i.v. injection of Lipo+siClC3. (**B**) Quantification of the western blot data. Data are presented as mean ± SEM. Statistical significance was calculated by Student's t-test. n = 3 biological replicates per group, across 1 experiment. (**C**) Confocal immunofluorescence images of the siClC3-treated ALI liver portrayed modest endosomal escape of Au@PDA@mHGF NW. White number indicates PCC between Cy5-labeled mHGF (red) and lysosomes (green; labeled by anti-LAMP1 antibody). Blue = nuclei. The two smaller images on the right show the enlargement of the boxed areas (white) of the larger image on the left. White arrows in the two smaller images denote the colocalization of red and green signals, indicating unsuccessful endosomal escape.

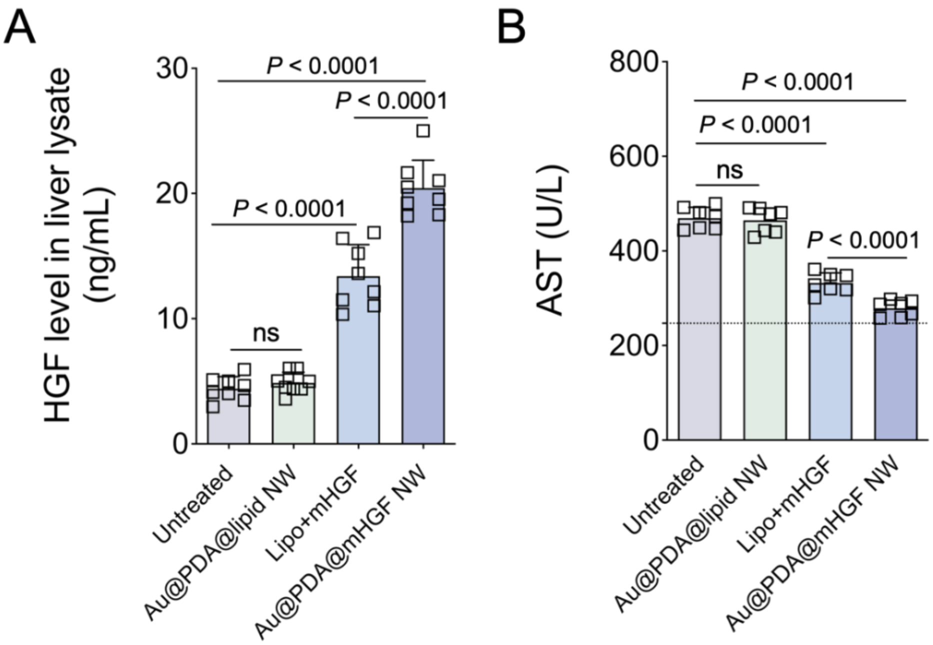

Figure S54. HGF expression and serum AST level 24 h post-treatment. (**A**) HGF expression in the liver 24 h post-injection measured by ELISA. (**B**) Au@PDA@mHGF most effectively decrease the level of AST by blood liver function test. Data are presented as mean ± SEM. Statistical significance was calculated by one-way ANOVA with Tukey's Test for post-hoc analysis. ns = not significant (P > 0.05). n = 7 biological replicates per group, across 2 experiments. Dotted line denotes the upper limit of the normal range.

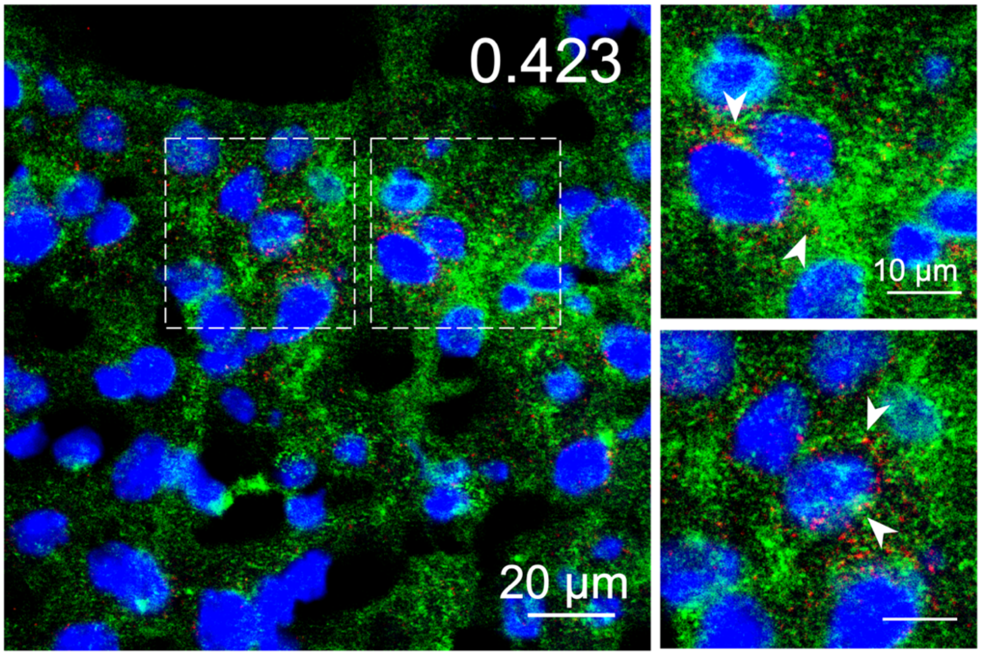

Figure S55. Endosomal escape efficiency of Lipo+mHGF in the liver 24 h post-i.v. injection into ALI mice. Confocal immunofluorescence images of the ALI liver portrayed modest

endosomal escape of Lipo+mHGF. White number indicates PCC between Cy5-labeled mHGF (red) and lysosomes (green; labeled by anti-LAMP1 antibody). Blue = nuclei. The two smaller images on the right show the enlargement of the boxed areas (white) of the larger image on the left. White arrows in the two smaller images denote the colocalization of red and green signals, indicating unsuccessful endosomal escape.

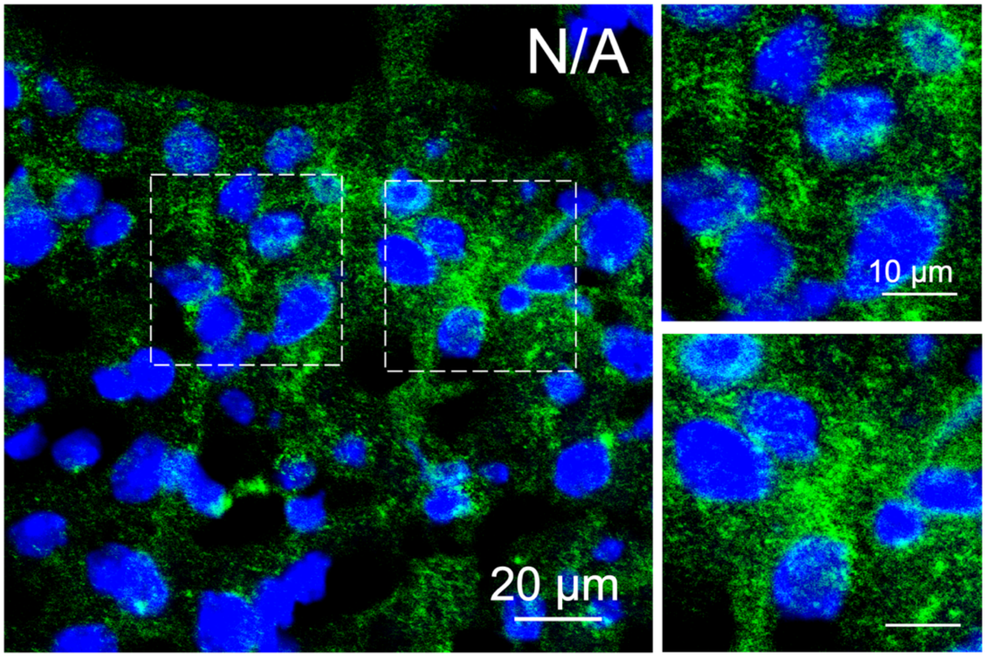

Figure S56. In vivo endosomal escape of PEI+mHGF in the liver 24 h post-injection into ALI mice. Confocal immunofluorescence images of the ALI liver portrayed limited accumulation of Lipo+mHGF (red). The PCC between PEI+mHGF and lysosomes (labeled by the anti-LAMP1 antibody) cannot be calculated due to the limited signals of mHGF in the liver (therefore listed as N/A, or not applicable). Blue = nuclei. The two smaller images on the right show the enlargement of the boxed areas (white) of the larger image on the left.

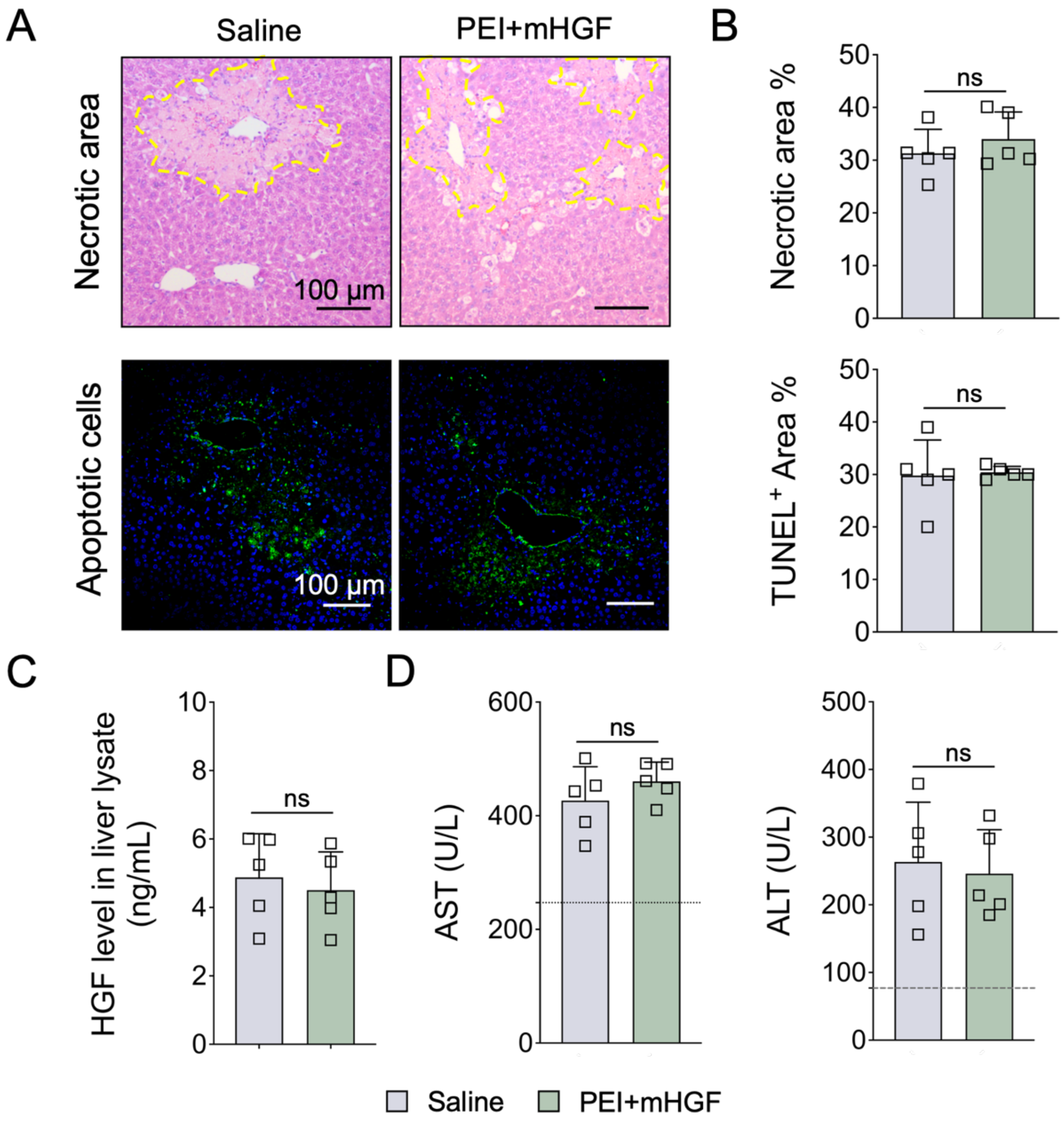

Figure S57. Efficacy of PEI+mHGF 24 h post-i.v. injection into ALI mice. (**A**) Necrotic areas (encircled by dotted yellow line) and apoptotic cells (TUNEL-positive; green) in the liver. (**B**) Quantification of necrotic and TUNEL-positive areas based on (**A**). **(C)** HGF expression in the liver measured by ELISA. **(D)** Serum levels of AST and ALT. Dotted line denotes the upper limit of the normal blood enzyme concentrations. Data are presented as mean ± SEM. All statistics are one-way ANOVA with Tukey's multiple comparisons test. ns = not significant ($P > 0.05$). n = 5 biological replicates per group, across 1 experiment.

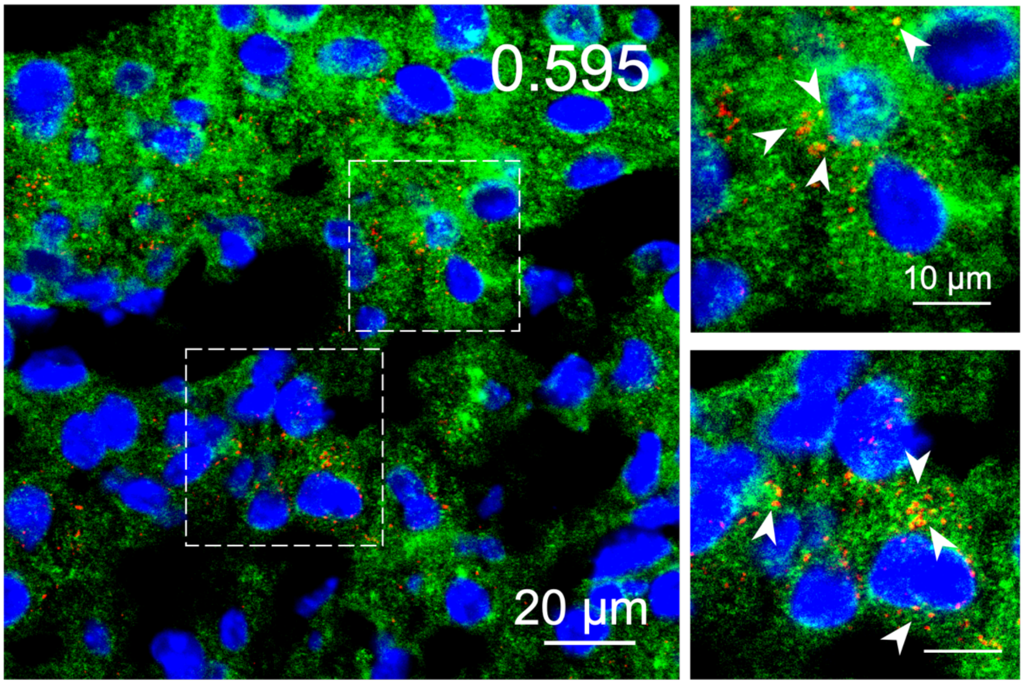

Figure S58. In vivo endosomal escape of conventional liposome+mHGF in the liver 24 h post-injection into ALI mice. White number indicates PCC between Cy5-labeled mHGF (red) and lysosomes (labeled by the anti-LAMP1 antibody). Blue = nuclei. The two smaller images on the right show the enlargement of the boxed areas (white) of the larger image on the left.

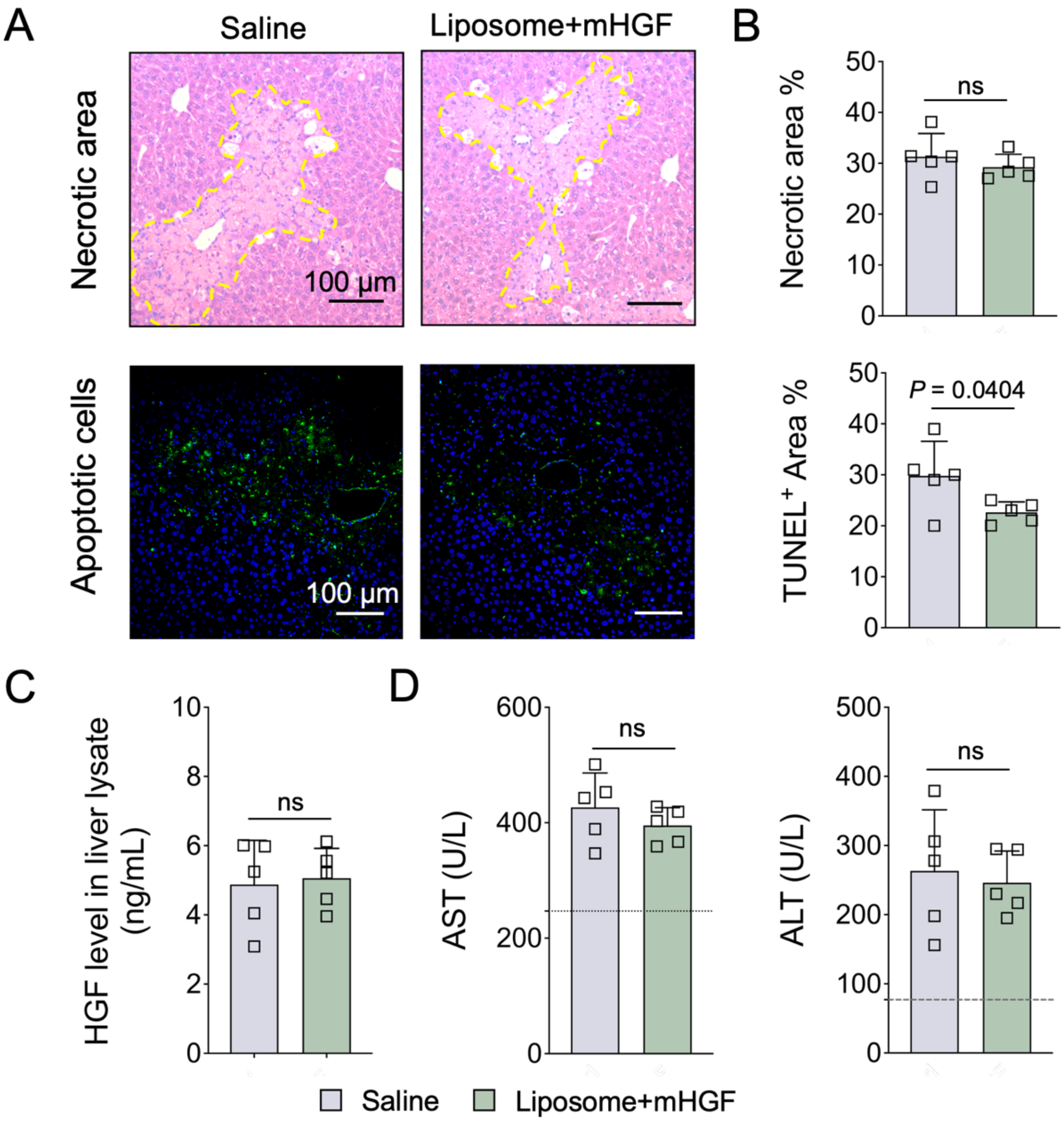

Figure S59. Efficacy of conventional liposome+mHGF 24 h post-i.v. injection into ALI mice. (**A**) Necrotic areas (encircled by dotted yellow line) and apoptotic cells (TUNEL-positive; green) in the liver. (**B**) Quantification of necrotic and TUNEL-positive areas based on (**A**). **(C)** HGF expression in the liver measured by ELISA. **(D)** Serum levels of AST and ALT. Dotted line denotes the upper limit of the normal blood enzyme concentrations. Data are presented as mean ± SEM. All statistics are one-way ANOVA with Tukey's multiple comparisons test. ns = not significant ($P > 0.05$). n = 5 biological replicates per group, across 1 experiment.

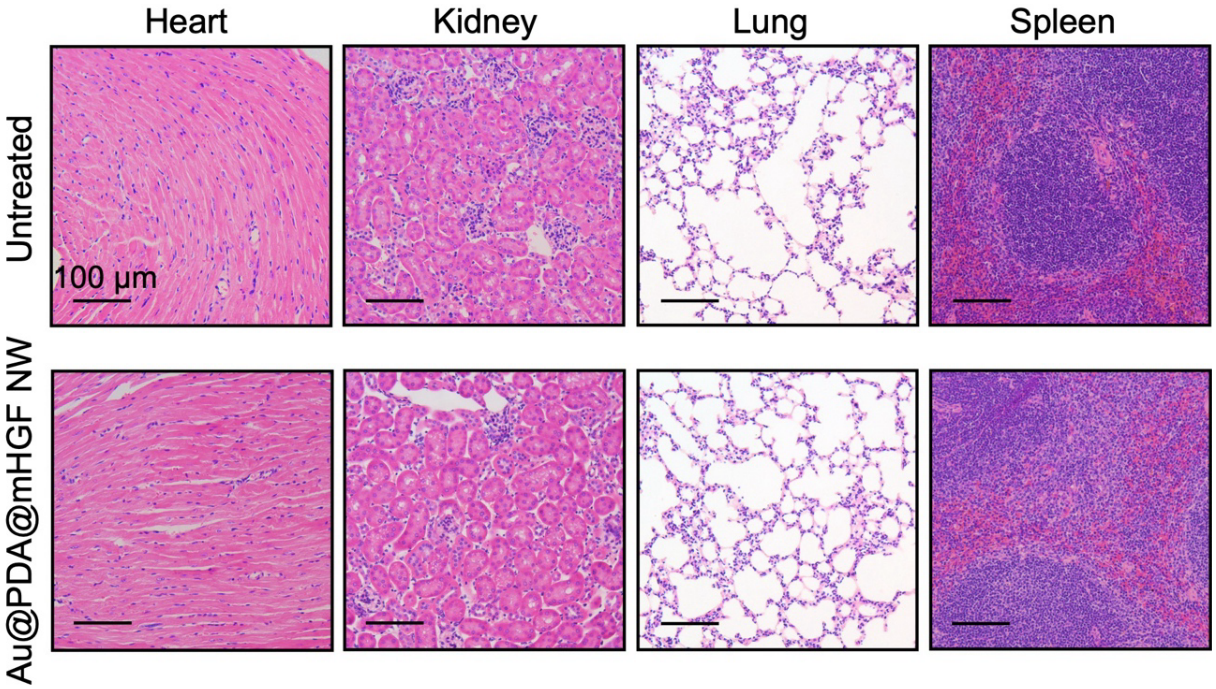

Figure S60. Short-term in vivo toxicity of Au@PDA@mHGF NWs in ALI mice [24 h post-single i.v. injection (or 48 h post-APAP disease induction)]. ALI mice received a single i.v injection of 0.5 mg of Au@PDA@mHGF NWs (containing 10 µg of mHGF) and were sacrificed 24 h post-injection. Histological examination of the major internal organs showed Au@PDA@mHGF NW led to no appreciable change in tissue morphology. Representative images were chosen from n = 3 mice per group, across 1 experiment.

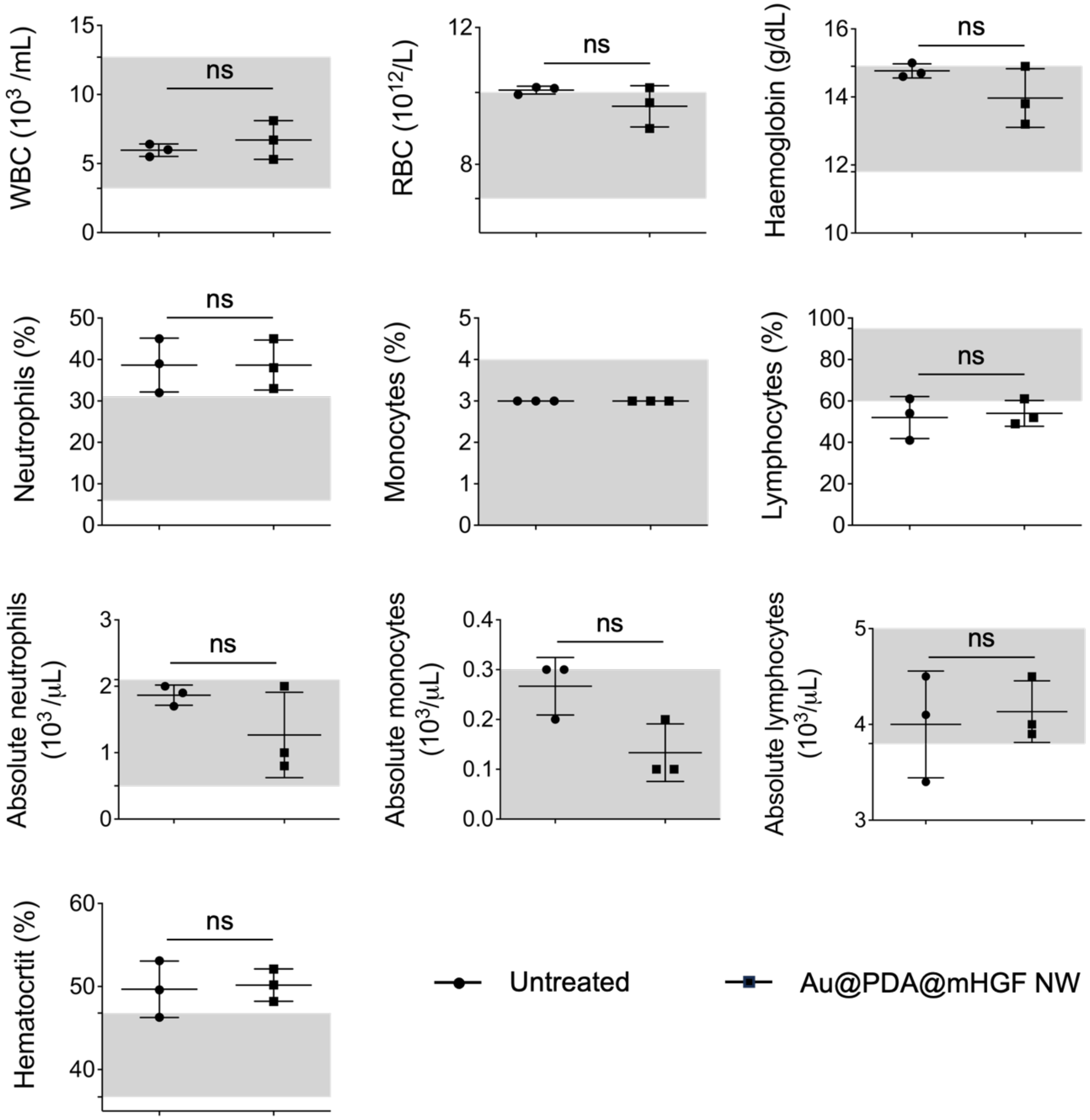

Figure S61. Short-term in vivo toxicity of Au@PDA@mHGF NWs in ALI mice [24 h post-single i.v. injection (or 48 h post-APAP disease induction)]. Au@PDA@mHGF NWs did not change blood chemistry or cell count. Grey shaded area represents the normal range of markers or cell counts for mice. WBC: white blood cell. RBC: red blood cell. Absolute neutrophils, lymphocytes, or monocytes (10^3/μL): cell number per volume of blood. Neutrophils%, lymphocytes%, monocytes%: percentage cells among total WBC. RBC (10^6/μL): cell number per volume of blood. Data are presented as mean ± SEM. Statistical significance was calculated by Student's t-test. ns: not significant ($P > 0.05$). $n = 3$ mice per group, across 1 experiment. The test on monocytes (%) showed identical values for both groups, so statistical analysis could not be performed; all values were within normal range.

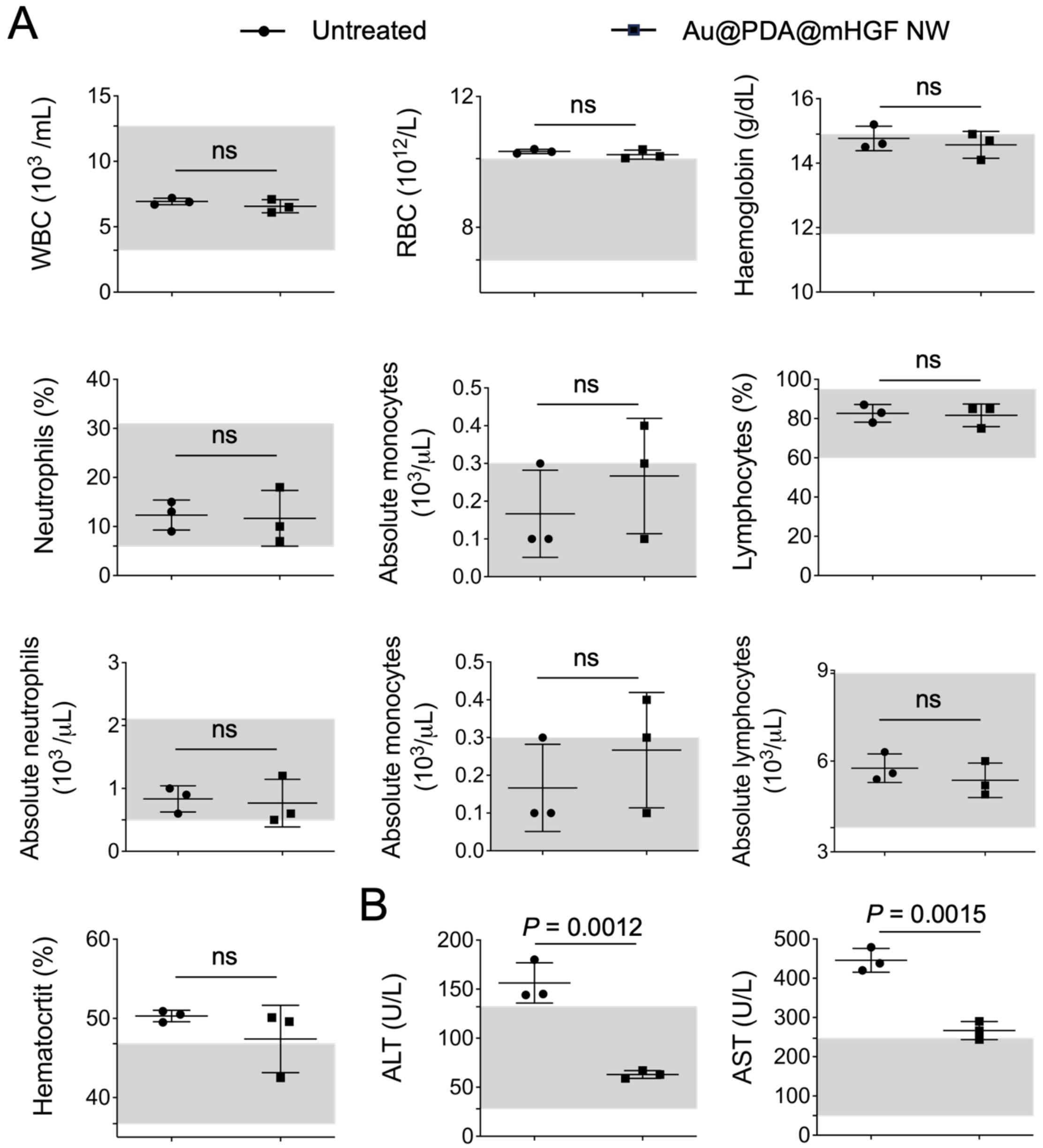

Figure S62. Long-term in vivo toxicity of Au@PDA@mHGF NW in ALI mice 8 months post-single i.v. injection. (**A**) Au@PDA@mHGF NW did not change blood chemistry or cell count. WBC: white blood cell. RBC: red blood cell. Absolute neutrophils, lymphocytes, or monocytes (10^3/µL): cell number per volume of blood. Neutrophils%, lymphocytes%, monocytes%: percentage cells among total WBC. RBC (10^6/µL): cell number per volume of blood. (**B**) Au@PDA@mHGF NW improved serum ALT and AST levels compared to untreated ALI mice. Grey shaded area represents the normal range of markers or cell counts for mice. Data are presented as mean ± SEM. Statistical significance was calculated by Student's t-test. n = 3 mice per group, across 1 experiment.

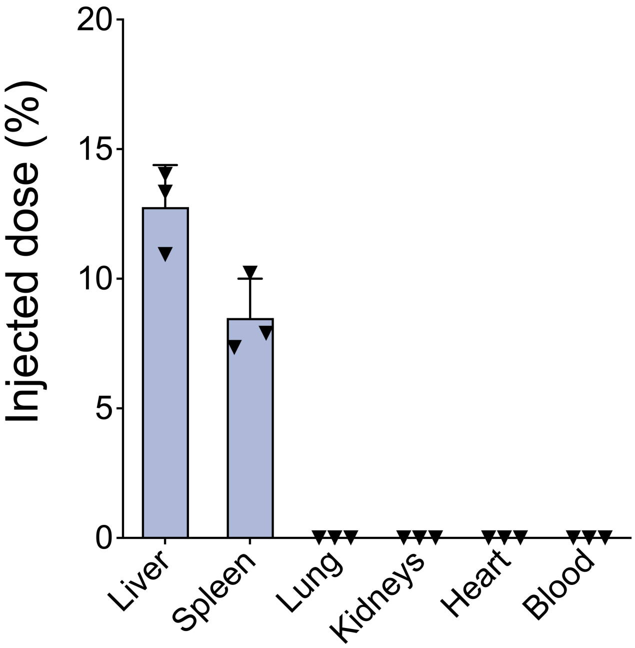

Figure S63. Long-term organ-level distribution of Au@PDA@mHGF NW in ALI mice 8 months post-single i.v. injection. ICP-MS analysis revealed that >60% of Au@PDA@mHGF NWs were cleared from the liver and spleen 8 months post-injection when compared to the distribution data obtained 24 h post-injection. Moreover, there was no detectable accumulation of Au@PDA@mHGF NW in other major organs and blood 8 months post-injection. Data are presented as mean ± SEM. n = 3 mice per group, across 1 experiment.

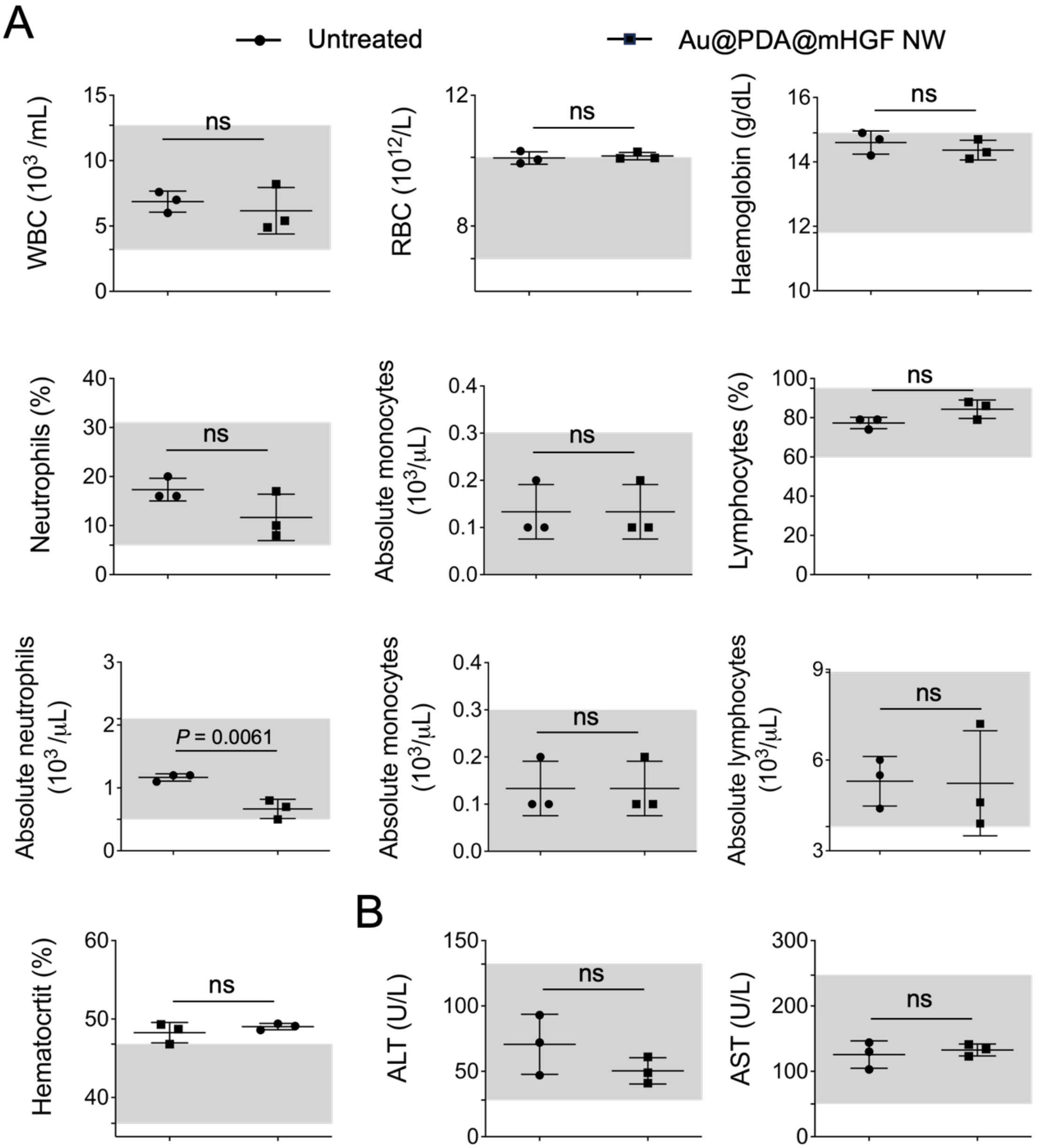

Figure S64. Long-term in vivo toxicity of Au@PDA@mHGF NW in healthy C57 mice 7 months post-single i.v. injection. Au@PDA@mHGF NW did not change (**A**) blood chemistry or cell count, and (**B**) serum ALT and AST levels. Grey shaded area represents the normal range of markers or cell counts for mice. WBC: white blood cell. RBC: red blood cell. Absolute neutrophils, lymphocytes, or monocytes (10^3/μL): cell number per volume of blood. Neutrophils%, lymphocytes%, monocytes%: percentage cells among total WBC. RBC (10^6/μL): cell number per volume of blood. Data are presented as mean ± SEM. Statistical significance was calculated by Student's t-test. ns: not significant ($P > 0.05$). n = 3 mice per group, across 1 experiment.

Supplementary Tables

Table S1. Endosomal escape of NPs for gene delivery with a colocalization coefficient <0.5 between the NP-gene complex and acidic vesicle (e.g., endosome and lysosome) from January 2019 to July 2025. Inclusion criteria are (i) NPs designed strictly for therapeutic gene delivery (not for imaging or diagnostics) and (ii) original research publications (not reviews) that explicitly mentioned "endosomal escape" in the manuscript and contained experimental data on the colocalization coefficients. This table lists various NPs in ascending order of colocalization coefficient (from smallest to largest), with correlation coefficients ≤ 0.2 (indicating little or no spatial correlation) highlighted in bold.

NP type	Gene cargo	Size (nm)<br>Zeta potential (ζ) (mV)	Colocalization coefficient	Mechanism of escape	Cell type
Gold-polydopamine core-shell NW (This work)	ASO<br>miRNA<br>siRNA<br>mRNA	D_H: ~220 (W/O lipid)<br>~280 (W/ lipid)<br>ζ: < –30	Oligonucleotide: **0.1–0.2** (PCC)<br>mRNA: **0.091** (PCC)	ClC3 activation	A549<br>bEnd.3<br>BMDM<br>hMSC
Apolipoprotein modified LNP [2025 (*72*)]	mRNA	D_H: 139.7<br>ζ: N/A	**–0.01 (PCC)**	N/A	SK-OV-3
Hydroquinine-based NP [2023 (*73*)]	pDNA	D_H: 50–100<br>ζ: N/A	**–0.10 to 0.04** (PCC)	Proton sponge effect	HEK293T
PEI-coated gold nanorod [2020 (*45*)]	pDNA	D_H: 93.1 × 11.1<br>ζ: ~ +35	**–0.2 to 0.1**	Proton sponge effect	HEK293T
Janus base nanotube [2021 (*74*)]	siRNA	TEM: 204.8 ×41.7<br>ζ: ~ +5	**0.09** (PCC)	Proton sponge effect	C28/I2
pH-responsive PEG-b-PAMA-b-P(C7A-r-DBA) micelle [2024 (*75*)]	siRNA	D_H: ~70<br>ζ: ~ +8	**< 0.1** (PCC)	Proton sponge effect	ARPE-19

Sialic acid-modified LNP [2023 (*76*)]	mRNA	D_H: 167.0 ± 0.8 ζ: 0.014 ± 0.1	**< 0.1**	Membrane destabilization	DC2.4
NIR-II activatable poly(β-amino esters)-based NP [2023 (*77*)]	pDNA	D_H: 82.8 ± 16.4 ζ: +3.6	**< 0.1** (PCC)	Proton sponge effect	B16F10
Fusogenic coiled-coil peptides modified LNP [2023 (*78*)]	mRNA	D_H: 94.1 ± 2.3 ζ: –4.37 ± 0.65	**0.06** (PCC)	Membrane fusion	HeLa
Light-triggerable polymeric NP library [2020 (*79*)]	siRNA miRNA	D_H: ~ 60 ζ: ~+20	**~ 0.1**	Light-triggered escape	HeLa Fibroblast Keratinocytes
Ultrasound-assisted fluorinated PEGylated LNP [2025 (*80*)]	mRNA	D_H: ~130 ζ: ~-5	**~0.1**	Membrane fusion	DC2.4
Hydrophobic-cored polymeric nanomicelle [2021 (*81*)]	siRNA	D_H: ~200 ζ: +19.5 to +26.0	**~ 0.14**	Proton sponge effect	HepG2-Luc
Amino acrylate-based NP [2024 (*82*)]	mRNA	D_H: 220–320 ζ: –2 to –4	**~ 0.15** (PCC)	Proton sponge effect	A549
Nucleic acid nanocapsule [2024 (*83*)]	DNA	TEM: 122 ± 1.7 ζ: -60	**0.18** (HeLa) **0.17** (A549) (PCC)	Membrane destabilization	HeLa A549
DNA nanodevice agonist [2024 (*84*)]	DNA	D_H: 40 ζ: N/A	**0.1–0.2** (MCC)	Endosomal releasing peptide	RAW264.7
Chitosan-modified polymetformin NP [2019 (*85*)]	pDNA	D_H: ~100 ζ: ~ +30	0.2	Proton sponge effect	HepG2

BAMPA-O16B lipid-incorporated LNP [2022 (*86*)]	siRNA	D_H: ~125<br>ζ: ~+5	0.2	Proton sponge effect	GL261
Red blood cell-derived extracellular vesicle [2023 (*87*)]	siRNA	D_H: ~200–226<br>ζ: ~ –20	0.08–0.22	N/A	HeLa<br>MOLM13
Oligoelectrolyte-conjugated LNP [2025 (*88*)]	mRNA	D_H: 67.9 ± 0.8<br>ζ: −2.0 to −4.0	0.2<br>(PCC)	N/A	HEK293
CaClOH-modified silica NP [2025 (*89*)]	mRNA	D_H: 236.9 ± 2.0<br>ζ: -7	0.25	Ca^{2+} release-mediated membrane rupture	HEK293
Amine-functionalized mesoporous silica NP [2025 (*90*)]	DNA	D_H: ~90<br>ζ: -0.033	0.2~0.6<br>(PCC)	Proton sponge effect	5637
Bioreducible PAMAM NP [2023 (*91*)]	mRNA	D_H: 52.3 ± 0.5<br>ζ: –3.4 ± 0.7	0.20 ± 0.09<br>(PCC)	Proton sponge effect	C28/I2
pH-responsive polycarboxybetaine-coated LNP [2022 (*92*)]	siRNA	D_H: 130 ± 3.8<br>ζ: +3.6 ± 2.9	~ 0.25–0.35	Proton sponge effect	SKOV3-luc<br>CT 26
Polymer-locking fusogenic liposome [2024 (*93*)]	siRNA	D_H: ~ 120<br>ζ: +6.1 ± 0.7	0.3<br>(PCC)	Membrane fusion	LN229R
PDMAEMA-POEGMA NP [2022 (*94*)]	siRNA	D_H: 14.4<br>ζ: +8.5	~ 0.3	Proton sponge effect	A549
Caffeic acid-coated magnetic calcium phosphate NP [2020 (*95*)]	siRNA	D_H: 134.7 ± 0.896<br>ζ: −3.06	~ 0.3	Magnetic field-triggered escape	HCC1954

Photosensitizer and targeting ligand-caped triblock copolymeric NP [2025 (*96*)]	siRNA	D_H: 145.6<br>ζ: +15	0.35	N/A	B16F10
Disulphide glutathione-responsive nanogel [2025 (*97*)]	mRNA	D_H: ~150<br>ζ: +20 to +25	0.32–0.38 (PCC)	Proton sponge effect	HeLa
Peptide-mRNA polyplexes [2024 (*98*)]	mRNA	D_H: 75~98<br>ζ: /	0.3~0.4 (PCC)	Endosomal membrane disruption	MC3T3
Nordihydroguaiaretic acid-cross-kinked phenylboronic acid-modified micelle [2024 (*99*)]	pDNA	D_H: ~100<br>ζ: +4.11–4.65	0.4	N/A	4T1
Lipid-PLGA core-shell NP [2024 (*100*)]	siRNA	D_H: ~ 300<br>ζ: ~ –8	0.35 (PCC)	Membrane fusion	A549
PEI-conjugated $NaGdF_4$:20 %Yb, 2 %Er@$NaGdF_4$ core–shell upconversion NP [2022 (*20*)]	DNAzy-me	D_H: ~100<br>ζ: –18	0.35 (PCC)	ROS induced rupture upon NIR irradiation	HeLa
ICG-loaded mesoporous silica NP [2020 (*101*)]	siRNA miRNA	TEM: ~160<br>ζ: –19	0.35	Light-triggered escape	MDA-MB-231
Metal-organic@metal-DNA core-shell NP [2023 (*102*)]	ASO	D_H: 100–150<br>ζ: ~ –12	0.36 (PCC)	Light-triggered production of ROS	MCF-7
Tannic acid LNP [2023 (*103*)]	mRNA	D_H: 221.5 ± 2.8<br>ζ: ~ +5 (pH 5.0)	0.37 ± 0.05 (PCC)	Membrane destabilization and proton sponge effect	MDCK

Oligonucleotide-phytoglycogen nanocomplex [2024 (*104*)]	DNA	D_H: 105 ± 35<br>ζ: +18 ± 5	~ 0.4 (PCC)	Proton sponge effect	PC3
pH-responsive AuNP-cored SNA [2024 (*105*)]	ASO	D_H: 15.1 ± 0.6<br>ζ: N/A	~ 0.4 (MOC)	Membrane destabilization	HeLa
pH activatable NIR-II dye conjugated LNP [2023 (*106*)]	mRNA	D_H: 133.6<br>ζ: −5.1 ± 0.7	~ 0.4	Light-triggered release	HEK293
Light-activable porphyrin LNP [2023 (*107*)]	siRNA	D_H: 59.72 ± 2.24<br>ζ: N/A	~ 0.4 (PCC)	Laser irradiation	PC3<br>4T1<br>A549<br>PANC-1<br>SKOV-3
Acid-degradable LNP [2024 (*108*)]	mRNA<br>DNA	TEM: ~100<br>ζ: –21	~ 0.4	Membrane destabilization	HeLa
Dendritic cell-mimicking NP [2023 (*109*)]	mRNA	D_H: 150.13 ± 11.68<br>ζ: −11.3 ± 2.4	0.38–0.42 (PCC)	N/A	HEK293T<br>DC2.4
Peptide functionalised magnetic NP [2024 (*110*)]	DNA	D_H: 221<br>ζ: −16	0.432 ± 0.085 (PCC)	Peptide mediated membrane translocation	Astrocyte<br>microglia<br>neuron
Ionizable MC3 lipid-incorporated LNP [2024 (*111*)]	mRNA	D_H: 190<br>ζ: ~ +30	0.46 ± 0.12 (PCC)	Mechanical oscillation	FNE
Ionizable SM-102 lipid-incorporated LNP [2024 (*112*)]	mRNA	D_H: 72 ± 6<br>ζ: N/A	0.47 (MOC)	N/A	HEK293
Polyphenolic NP library [2024 (*113*)]	mRNA	D_H: 100–120<br>ζ: ~ –2 to 0	0.4–0.5 (PCC)	Proton sponge effect	DC 2.4

Table S2. List of reports on endosomal escape of NPs for gene delivery that do not report a colocalization coefficient between the NP-gene complex and acidic vesicle (e.g., endosome and lysosome) from January 2019 to July 2025. Inclusion criteria are (i) NPs designed strictly for therapeutic gene delivery (not for imaging or diagnostics) and (ii) original research publications (not reviews) that explicitly mentioned "endosomal escape" in the manuscript but devoid of experimental or quantitative data on the colocalization coefficients. This table lists the NPs in descending order of publication year.

NP type	Gene cargo	Size (nm)<br>Zeta potential<br>(ζ) (mV)	Mechanism of endosomal escape	Cell type
Gold-polydopamine core-shell NW<br>(This work)	ASO<br>miRNA<br>siRNA<br>mRNA	D_H: ~220 nm<br>(W/O lipid)<br>~280 nm<br>(W/ lipid)<br>ζ: < −30 mV	ClC3 activation	A549<br>bEnd.3<br>BMDM<br>hMSC
Polypeptide-based nanoconjugate<br>[2025 (*114*)]	pDNA	D_H: 51 ± 9<br>ζ: 0.93 ± 1.8	N/A	MDA-MB-231
Cetuximab-modified cationic liposome<br>[2025 (*115)*]	siRNA	D_H: 159.2 ± 0.8<br>ζ: 4.9 ± 0.5	Membrane destabilization	A549
Polymeric nanocomplex<br>[2025 (*116*)]	siRNA	D_H: 114.4 ± 1.6<br>ζ: 17.0 ± 0.5	pH responsive cationic polymer	4T1
Neutrophil membrane-camouflaged nanocomplex<br>[2025 (*117*)]	siRNA	D_H: ~230 nm<br>ζ: − 6.47 ± 2.46	Hemagglutinin-mediated escape	mouse neutrophil
GSH-responsive PDPA micelle [2025 (*118*)]	siRNA	D_H:<br>172.2 ± 1.834<br>ζ: −40	Proton sponge effect	MG63
Gold-cored SNA<br>[2025 (*119*)]	Oligonucl-eotide	D_H: 15<br>ζ: /	N/A	HeLa
Mesoporous copper poly-tannic acid NP<br>[2025 (*120*)]	miRNA	D_H: 328.24<br>ζ: −26	Membrane destabilization	Human gingival fibroblast

Disulfide cross-linked polyethylenimine polyplex [2025 (*121*)]	Oligonucl-eotide	D_H: 97.4 ± 15.1<br>ζ: 21.08 ± 8.16	N/A	HeLa
Amino acryloyl-modified lactoferrin acrylamide-alendronate core-shell nanocapsule [2025 (*122*)]	siRNA	D_H: ~70<br>ζ: −7.79 ± 0.13	Proton sponge	RAW 264.7
ROS-responsive cinnamaldehyde-based cationic polymeric NP [2025 (*123*)]	DNA	D_H: ~125<br>ζ: ~10	ROS-induced membrane disruption	HeLa
Branched endosomal disruptor LNP [2025 (*124*)]	mRNA	D_H: 100 ± 44<br>ζ: −11.3	Membrane destabilization	Primary human T cells
Fluorinated polymeric NP library [2025 (*125*)]	mRNA	D_H: 223 ± 4<br>ζ: +44.9 ± 0.8	N/A	HeLa
Fusogenic lipid nanovesicle [2024 (*126*)]	mRNA	D_H: ~90<br>ζ: /	Membrane fusion	BMDC
Cholesterol-enriched exosome [2024 (*127*)]	siRNA	D_H: ~140<br>ζ: −12	Membrane fusion	HCT116
Ionizable SM-102 lipid-incorporated LNP [2024 (*128*)]	mRNA	D_H: 113.9 ± 1.8<br>ζ: −2.6 ± 0.3	N/A	C2C12 B16
Histidine oligomer-incorporated LNP [2024 (*129*)]	siRNA	D_H: 65.3 ± 9.1<br>ζ: ~ −7	Membrane fusion and proton sponge effect	A549
Surfactin-based LNP [2024 (*130*)]	mRNA	D_H: 84.91 ± 6.24<br>ζ: +1.3 ± 0.7	Proton sponge effect and membrane fusion	HeLa HepG2
Caveolin-incorporated nanovesicle [2024 (*131*)]	pDNA	TEM: ~50<br>ζ: N/A	N/A	A431

Covalent polymer-RNA nanoconjugate [2024 (*132*)]	RNA	D_H: ~35<br>ζ: ~ 0	Membrane destabilization	Gal8-MDA-MB-231
Fluorinated LNP [2024 (*133*)]	mRNA	D_H: 137.2 ± 0.1<br>ζ: –5 ± 2	N/A	B16F10
Ionizable LNP formed with piperazine-derived lipid [2024 (*134*)]	mRNA	D_H: ~80<br>ζ: ~ +20 (pH 4.0) near neutral (pH 7.4)	Membrane fusion	CT26
Zeolitic imidazolate framework-8 NP [2024 (*135*)]	miRNA	D_H: ~200<br>ζ: ~ –30	Proton sponge effect	Neonatal rat cardiomyocytes
Amidine-incorporated degradable LNP [2024 (*136*)]	mRNA	D_H: ~73.4<br>ζ: +1.63	Benzene ring induced membrane destabilization	HepG2
Polypept(o)ide-based polyion complex micelle [2024 (*137*)]	siRNA	D_H: ~75<br>ζ: ~ +8	Desloratadine-mediated escape	H1299-GFP
Bis (2,4,6-trichlorophenyl) oxalate and hemin functionalized ionizable lipopeptide LNP [2024 (*138*)]	siRNA	D_H: ~300<br>ζ: ~ –7	ROS generation	HeLa
Ionizable MC3 lipid-incorporated LNP [2024 (*139*)]	siRNA<br>mRNA	Confocal microscopy: ~200<br>ζ: N/A	N/A	HeLa
Vinpocetine-derived ionizable-lipidoid NP [2024 (*140*)]	siRNA	D_H: 93.7 ± 4 .4<br>ζ: +1.7 ± 0.5	Proton sponge effect	bEnd.3
pH-responsive lipoamino xenopeptide LNP [2024 (*141*)]	siRNA	D_H: ~150<br>ζ: ~ –2	N/A	HeLa

pH-responsive lipopeptide-functionalized NP [2024 (*142*)]	siRNA	TEM: 40–60<br>ζ: ~ +25<br>(pH 5)	Proton sponge effect	HEK293T
Polyhistidine-incorporated LNP [2023 (*143*)]	siRNA	D_H: ~100<br>ζ: −13.3 ± 4.2	Membrane destabilization	A549
Iron oxide-silica core-shell NP [2024 (*144*)]	siRNA	TEM: ~150<br>ζ: ~ +12	N/A	MDA-MB-231
PEG/PLL-based polymersome [2023 (*145*)]	mRNA	D_H: 105.03<br>ζ: +18.1	Proton sponge effect	RAW 264.7
Fluorinated peptide-conjugated dendritic poly-lysine NP [2023 (*146*)]	siRNA	D_H: 186.1<br>ζ: ~ +40	N/A	HepG2
Lipofectamine 2000-siRNA lipoplex [2023 (*147*)]	siRNA	Confocal imaging: 1000–3000<br>ζ: N/A	Membrane fusion	HeLa
Cationic lipid and mannose-conjugated PLGA hybrid NP [2023 (*148*)]	mRNA	D_H: 122.1 ± 1.1<br>ζ: +2.33 ± 0.12	N/A	BMDM
Ionizable polyester NP [2022 (*149*)]	pDNA<br>mRNA	D_H: ~200<br>ζ: > +40	Proton-sponge effect	RAW 264.7
Ionizable LNP library [2023 (*150*)]	siRNA	D_H: ~160<br>ζ: ~ +10 to +15	Membrane destabilization	RAW 264.7
DSPE-PEG-coated PLGA-cored NP [2022 (*151*)]	mRNA	TEM: ~130<br>ζ: N/A	Proton sponge effect	Kdm6a-null KU19-19
Polymeric NP with varying bisacrylamides and amines [2023 (*152*)]	mRNA and gRNA	D_H: 200–600<br>ζ: ~ +5 to +25	Proton sponge effect	Human dermal fibroblast
ROS-biodegradable LNP library [2022 (*153*)]	mRNA	TEM: ~140<br>ζ: +12 to +18	Membrane destabilization and proton sponge effect	HeLa
PAA-PGA core-shell NP [2022 (*154*)]	pDNA	D_H: 170.7 ± 6.6<br>ζ: +14.9 ± 0.3	Proton sponge effect	HEK293T

Virus-mimicking zeolitic imidazolate framework-90 NP [2022 (*155*)]	DNAzyme	D_H: 160<br>ζ: –22	Membrane fusion	4T1
Poly(beta-amino ester)s-based NP [2022 (*36*)]	mRNA	D_H: ~100<br>ζ: > +40	Membrane interaction	B16-F10
GSH-responsive silica NP [2021 (*156*)]	DNA	D_H: 45<br>ζ: + 6.4	Proton sponge effect	HEK293
NIR-activatable PDA NP [2021 (*157*)]	pDNA	D_H: ~240<br>ζ: +20 to +30	Laser-triggered escape	HepG2
PEI-modified silk fibroin NP [2021 (*158*)]	siRNA	D_H: 203.8 ± 0.5<br>ζ: +11 ± 1.4	Proton sponge effect	4T1
Virus-mimicking membrane coated NP [2022 (*159*)]	mRNA	D_H: 185<br>ζ: −20	Membrane fusion	B16-WT
PEI-PAMAM assembled NP [2020 (*160*)]	pDNA	D_H: 120<br>ζ: +1.6	Proton sponge effect	A549
Silica-metal-organic framework hybrid NP [2020 (*161*)]	sgRNA	D_H: 110<br>ζ: +5.6 ± 1	Proton sponge effect	HEK293
Self-degradable ionizable LNP [2020 (*162*)]	mRNA	D_H: 79.4 ± 8.0<br>ζ: −4.0 ± 1.3	Membrane destabilization	HeLa
PLGA-cored hybrid LNP [2020 (*163*)]	siRNA	D_H: 116.2 ± 2.5<br>ζ: −2.66 ± 1.01	N/A	HeLa
Platelet membrane-coated metal-organic framework NP [2020 (*164*)]	siRNA	D_H: ~175<br>ζ: ~ –30	N/A	SK-BR-3
Vitamin LNP [2020 (*165*)]	mRNA	D_H: ~ 140<br>ζ: ~ +21	N/A	RAW264.7
Lipid-like NP comprising a phenyl core and six alkyl lipid chains [2020 (*166*)]	mRNA	D_H: ~100<br>ζ: ~ –11	N/A	Hep3B
PEI-coated PDA NP [2020 (*167*)]	pDNA	D_H: 200–300<br>ζ: +21.1	Laser-triggered escape	HepG2

Porous silicon NP [2020 (*168*)]	miRNA	D_H: ~270–300<br>ζ: Near neutral	Proton sponge effect	Gal8-MDA-MB231
Peptide assembled NP [2019 (*169*)]	siRNA	D_H: 175.63 ± 0.99<br>ζ: ~ +12	Proton sponge effect	4T1
Branched DNA self-assembled NP [2019 (*19*)]	sgRNA<br>Cas9<br>ASO	D_H: 92.9 ± 14.8<br>ζ: N/A	Peptide-mediated escape	MCF7

Abbreviation for Table S1 and Table S2

D_H: Hydrodynamic size; TEM: transmission electron microscope; PCC: Pearson correlation coefficient; MOC: Mander's overlap coefficient; N/A: not available; NW: nanoworm; NP: nanoparticle; ASO: antisense oligonucleotide; LNP: lipid nanoparticle; SNA: spherical nucleic acid; pDNA: plasmid DNA; PDPA: poly(2-(diisopropylamino) ethyl methacrylate); PEG: polyethylene glycol; PEI: polyethylenimine; PAMA: polyalkylmethacrylates; C7A: 2 (hexamethylenediamine) ethanol; DBA: dibenzylideneacetone; BAMPA-O16B: a disulfide bond-containing ionizable cationic lipid; PDMAEMA: poly[2-(dimethylamino) ethyl methacrylate]; POEGMA: poly [oligo (ethylene glycol) methyl ether methacrylate]; PLGA: poly lactic-co-glycolic acid; ICG: indocyanine green; NIR: near-infrared; DSPE: 1,2-distearoyl-snglycero-3-phosphoethanolamine; PLL: polylysine; ROS: reactive oxygen species; PAA: peroxyacetic acid; PGA: phosphoglyceric acid; GSH: glutathione; PAMAM: poly(amidoamine).

The hydrodynamic size of Au@PDA NW measured by dynamic light scattering (DLS) based on the Stokes–Einstein equation for spherical particles may not be accurate (*170*). Rodríguez-Fernandez *et al.* derived an expression for the translational diffusion coefficient of short NRs as a function of aspect ratio (*171*) by fitting empirical DLS data of cetyltrimethylammonium bromide-capped Au NRs with an aspect ratio between 4.2 and 5.5 to an established equation for the translational diffusion coefficient of rodlike particles (*172*). This equation is suitable for our analysis because Au@PDA NWs mostly bear 4–5 cores per worm. By extracting the diffusion coefficient of Au@PDA NWs from our DLS data and applying it to the equation by Rodríguez-Fernandez *et al.*, we obtained an estimated length of ~379 nm for Au@PDA NW, ~170 nm longer than our TEM data. Such a discrepancy may stem from the limitations that Au@PDA NW is not perfectly straight and contains a PDA shell not taken into account by the derived equation (*21*).

Table S3. Physicochemical characterization of various oligonucleotide-encased Au@PDA NWs, T_{21}-encased Au@PDA NPs, and T_{21}-encased NRs by DLS. PDI = polydispersity index.

Samples	Hydrodynamic diameter (nm)				ζ-potential in 1 mM KCl at 25 °C (mV)
	In water (25°C)	PDI	DMEM+10 % FBS (37 °C, 24 h)	PDI	
Cit-Au NP	41.5 ± 1.2	0.106	/	/	–30.2 ± 2.1
Au@PDA NP	81.7 ± 2.1	0.121	90.3 ± 3.2	0.209	–25.7 ± 2.5
Au@PDA@T_{21} NP	86.2 ± 0.9	0.101	91.2 ± 2.9	0.211	–30.6 ± 1.7
Cy5-labeled Au@PDA@T_{21} NP	86.5 ± 2.7	0.194	93.4 ± 1.8	0.192	–32.3 ± 2.6
Au@PDA NW	205.1 ± 3.4	0.125	218.2 ± 3.9	0.231	–25.7 ± 2.5
Au@PDA@T_{21} NW	220.2 ± 1.2	0.138	232.4 ± 4.2	0.198	–37.8 ± 1.3
Cy5-labeled Au@PDA@T_{21} NW	219.4 ± 3.1	0.200	226.9 ± 3.6	0.174	–34.7 ± 3.1
Cy5-labeled Au@PDA@A_{21} NW	219.5 ± 3.5	0.164	240.5 ± 1.3	0.231	–33.0 ± 1.4
Au@PDA@asEGFP NW	218.1 ± 2.9	0.115	232.1 ± 1.9	0.143	–34.9 ±2.1
Cy5-labeled Au@PDA@asEGFP NW	220.1 ± 1.2	0.152	230.6 ± 3.1	0.191	–33.2 ± 2.1

Samples	Hydrodynamic diameter (nm)				ζ-potential in 1 mM KCl at 25 °C (mV)
	In water (25°C)	PDI	DMEM+10 % FBS (37 °C, 24 h)	PDI	
Au@PDA@miR-223 NW	220.1 ± 3.8	0.143	235.1 ± 9.4	0.203	–39.4 ± 4.4
Cy5-labeled Au@PDA@miR-223 NW	218.5 ± 2.9	0.080	231.0 ± 2.3	0.221	–30.4 ± 6.1
Au@PDA@siNog NW	221.2 ± 6.2	0.125	230.7 ± 5.3	0.263	–33.4 ± 1.5
Cy5-labeled Au@PDA@siNog NW	220.4 ± 5.4	0.200	230.3 ± 5.3	0.198	–33.6 ± 4.2
Au@PDA NR	255.1 ± 3.6	0.186	280.7 ± 5.4	0.196	–28.5 ± 2.1
Au@PDA@T_{21} NR	260.1 ± 2.1	0.201	282.7 ± 2.5	0.232	–32.9 ± 0.9
Cy5-labeled Au@PDA@T_{21} NR	258.9 ± 3.4	0.192	278.1 ± 1.4	0.212	–31.0 ± 1.1

Table S4. Oligonucleotide loading on Au@PDA NW as determined by a fluorescence assay.

**Samples**	**No. of oligonucleotide strands per Au@PDA NW**
Cy5-labeled Au@PDA@T_{21} NW	1024 ± 12
Cy5-labeled Au@PDA@A_{21} NW	951 ± 7
Cy5-labeled Au@PDA@asEGFP NW	1040 ± 8
Cy5-labeled Au@PDA@miR-223 NW	998 ± 20
Cy5-labeled Au@PDA@siNog NW	910 ± 11

Table S5. Top enriched GO terms identified from DETs in A549 cells based on the pairwise comparison of "Au@PDA@T_{21} NW 24 h" group to "Au@PDA@T_{21} NP 24 h" group. Data are from n = 3, across 1 experiment. Q < 0.05. Bolded GO terms are relevant to intracellular vesicles and ion homeostasis.

**GO_Cellular component**	**Q value**
**Cytoplasm**	1.01E-11
Nucleus	3.84E-11
**Membrane**	1.37E-07
**Cytoskeleton**	5.97E-06
Microtubule bundle	2.09E-04
Nucleoplasm	8.30E-04
**Cytosol**	8.73E-04
Cortical microtubule	0.005535
Cytoplasmic microtubule bundle	0.005535
Chromosome	0.006253
Mitotic spindle pole	0.006455
**Late endosome**	0.044289
Spindle microtubule	0.008343
Perinuclear region of cytoplasm	0.011060
**Lysosomal lumen**	0.021961
Lateral cell cortex	0.011230
Microtubule organizing center	0.011495
**Vesicle**	0.032870
Microtubule cytoskeleton	0.014614

Table S6. Top enriched GO terms identified from DETs in A549 cells based on the pairwise comparison of "Au@PDA@T_{21} NW 24 h" group to "Au@PDA@T_{21} NW 8 h" groups. Data are from n = 3, across 1 experiment. Q < 0.05. Bolded GO terms are relevant to intracellular vesicles and ion homeostasis.

**GO_Cellular component**	**Q value**
**Membrane**	3.4E-125
Nucleus	1.7E-104
**Cytoplasm**	6.38E-77
Nucleoplasm	7.26E-75
**Cytosol**	2.60E-48
Cytoskeleton	2.38E-22
Spliceosomal complex	3.65E-22
Endoplasmic reticulum	3.41E-21
**Cytoplasmic vesicle**	5.37E-20
Chromosome	1.94E-19
Mitochondrion	4.67E-18
Extracellular exosome	6.61E-18
**Endosome**	1.07E-17
**Late endosome**	0.001159
Nuclear speck	3.12E-15
Nucleolus	6.68E-14
**Early endosome**	3.09E-04
**Lysosome**	8.36E-08
**Intracellular membrane-bounded organelle**	3.63E-06
**Endosome membrane**	6.54E-04
**Lumenal side of lysosomal membrane**	0.002188
**Transport vesicle**	0.002341
**Lysosomal membrane**	0.002870

Table S7. Physicochemical characterization of different types of mRNA-adsorbed NWs by DLS.

Sample	Hydrodynamic size (nm)				ζ-potential in 1 mM KCl at 25 ℃ (mV)
	In water (25 ℃)	PDI	α-MEM+20% FBS (37 ℃, 24h)	PDI	
Au@PDA@lipid NW	274.5 ± 4.3	0.198	290.1 ± 9.1	0.231	+30.5 ± 4.2
Au@PDA@mEGFP NW	280.3 ± 6.7	0.183	296.3 ± 7.3	0.210	–35.2 ± 3.1
Au@PDA@mHGF NW	283.5 ± 6.8	0.132	290.2 ± 8.4	0.199	–31.6 ± 2.9
Au@PDA@mCXCR4/mBMP7 NW	288.1 ± 4.2	0.200	298.4 ± 6.8	0.193	–33.7 ± 3.7

Table S8. Physicochemical characterization of benchmark gene carriers by DLS.

Sample	Hydrodynamic size		ζ-potential in 1 mM KCl at 25 ℃ (mV)
	In water (25 ℃)	PDI	
PEI+T_{21}	221.3 ± 3.8	0.293	+33.5 ± 1.1
PEI+mHGF	228.8 ± 4.1	0.219	+30.4 ± 4.5
LNP+T_{21}	99.2 ± 6.9	0.126	+9.1 ± 0.5
Lipo+T_{21}	330.1 ± 6.2	0.294	+31.5 ± 2.1
Lipo+mHGF	325.9 ± 3.1	0.315	+30.4 ± 1.7
Lipo+siNog	301.2 ± 3.0	0.194	+31.3 ± 3.1
Lipo+asEGFP	315.6 ± 2.8	0.293	+30.9 ± 1.2
Lipo+miR-223	307.4 ± 4.1	0.203	+30.7 ± 1.8
Lipo+mCXCR4/mBMP7	314.5 ± 3.1	0.193	+31.2 ± 1.7
Conventional liposome+mHGF	149.1 ± 2.5	0.182	–7.2 ± 3.0

Table S9. Loading of mRNA on Au@PDA@lipid NWs.

**Samples**	**No. of mRNA strands per Au@PDA@lipid NW**
Au@PDA@mEGFP NW	68 ± 5
Au@PDA@mHGF NW	25 ± 2
Au@PDA@mCXCR4/mBMP7 NW	29 ± 4

Table S10. Encapsulation efficiency of mHGF in lipid-based NPs.

**Samples**	**Encapsulation efficiency**
Conventional liposome+mHGF	13.5%
Lipofectamine+mHGF	86.1%

Data S1. Differentially expressed mRNA transcripts of shape-dependent effect by comparing "Au@PDA@T_{21} NW; 24 h" vs "Au@PDA@T_{21} NP; 24 h" groups.

Data S2. Differentially expressed mRNA transcripts of time-dependent effect by comparing "Au@PDA@T_{21} NW; 24 h" vs "Au@PDA@T_{21} NW; 8 h" groups.

REFERENCES

1. B. B. Mendes, J. Conniot, A. Avital, D. Yao, X. Jiang, X. Zhou, N. Sharf-Pauker, Y. Xiao, O. Adir, H. Liang, J. Shi, A. Schroeder, J. Conde, Nanodelivery of nucleic acids. *Nat. Rev. Methods Primers* **2**, 24 (2022).

2. J. Gilleron, W. Querbes, A. Zeigerer, A. Borodovsky, G. Marsico, U. Schubert, K. Manygoats, S. Seifert, C. Andree, M. Stöter, H. Epstein-Barash, L. Zhang, V. Koteliansky, K. Fitzgerald, E. Fava, M. Bickle, Y. Kalaidzidis, A. Akinc, M. Maier, M. Zerial, Image-based analysis of lipid nanoparticle-mediated siRNA delivery, intracellular trafficking and endosomal escape. *Nat. Biotechnol.* **31**, 638–646 (2013).

3. P. L. Felgner, T. R. Gadek, M. Holm, R. Roman, H. W. Chan, M. Wenz, J. P. Northrop, G. M. Ringold, M. Danielsen, Lipofection: A highly efficient, lipid-mediated DNA-transfection procedure. *Proc. Natl. Acad. Sci. U.S.A.* **84**, 7413–7417 (1987).

4. O. Boussif, F. Lezoualc'h, M. A. Zanta, M. D. Mergny, D. Scherman, B. Demeneix, J. P. Behr, A versatile vector for gene and oligonucleotide transfer into cells in culture and in vivo: Polyethylenimine. *Proc. Natl. Acad. Sci. U.S.A.* **92**, 7297–7301 (1995).

5. J. P. Behr, The proton sponge: A trick to enter cells the viruses did not exploit. *Chimia* **51**, 34–36 (1997).

6. X. Wei, B. Shao, Z. He, T. Ye, M. Luo, Y. Sang, X. Liang, W. Wang, S. Luo, S. Yang, S. Zhang, C. Gong, M. Gou, H. Deng, Y. Zhao, H. Yang, S. Deng, C. Zhao, L. Yang, Z. Qian, J. Li, X. Sun, J. Han, C. Jiang, M. Wu, Z. Zhang, Cationic nanocarriers induce cell necrosis through impairment of Na^+/K^+-ATPase and cause subsequent inflammatory response. *Cell Res.* **25**, 237–253 (2015).

7. H. Lv, S. Zhang, B. Wang, S. Cui, J. Yan, Toxicity of cationic lipids and cationic polymers in gene delivery. *J. Control. Release* **114**, 100–109 (2006).

8. X. Han, H. Zhang, K. Butowska, K. L. Swingle, M. G. Alameh, D. Weissman, M. J. Mitchell, An ionizable lipid toolbox for RNA delivery. *Nat. Commun.* **12**, 7233 (2021).

9. S. Chatterjee, E. Kon, P. Sharma, D. Peer, Endosomal escape: A bottleneck for LNP-mediated therapeutics. *Proc. Natl. Acad. Sci. U.S.A.* **121**, e2307800120 (2024).

10. S. Omo-Lamai, Y. Wang, M. N. Patel, E.-O. Essien, M. Shen, A. Majumdar, C. Espy, J. Wu, B. Channer, M. Tobin, S. Murali, T. E. Papp, R. Maheshwari, L. Wang, L. S. Chase, M. E. Zamora, M. L. Arral, O. A. Marcos-Contreras, J. W. Myerson, C. A. Hunter, A. Tsourkas, V. Muzykantov, I. Brodsky, S. Shin, K. A. Whitehead, P. Gaskill, D. Discher, H. Parhiz, J. S. Brenner, Lipid nanoparticle-associated inflammation is triggered by sensing of endosomal damage: Engineering endosomal escape without side effects. bioRxiv 589801 [Preprint] (2024); https://doi.org/10.1101/2024.04.16.589801.

11. S. A. Jensen, E. S. Day, C. H. Ko, L. A. Hurley, J. P. Luciano, F. M. Kouri, T. J. Merkel, A. C. Luthi, P. C. Patel, J. I. Cutler, W. L. Daniel, A. W. Scott, M. W. Rotz, T. J. Meade, D. A. Giljohann, C. A. Mirkin, A. H. Stegh, Spherical nucleic acid nanoparticle conjugates as an RNAi-based therapy for glioblastoma. *Sci. Transl. Med.* **5**, 209ra152 (2013).

12. Z. Wang, L. Song, Q. Liu, R. Tian, Y. Shang, F. Liu, S. Liu, S. Zhao, Z. Han, J. Sun, Q. Jiang, B. Ding, A tubular DNA nanodevice as a siRNA/chemo-drug co-delivery vehicle for combined cancer therapy. *Angew. Chem. Int. Ed.* **60**, 2594–2598 (2021).

13. T. Zhang, T. Tian, R. Zhou, S. Li, W. Ma, Y. Zhang, N. Liu, S. Shi, Q. Li, X. Xie, Y. Ge, M. Liu, Q. Zhang, S. Lin, X. Cai, Y. Lin, Design, fabrication and applications of tetrahedral DNA nanostructure-based multifunctional complexes in drug delivery and biomedical treatment. *Nat. Protoc.* **15**, 2728–2757 (2020).

14. J. Li, C. Zheng, S. Cansiz, C. Wu, J. Xu, C. Cui, Y. Liu, W. Hou, Y. Wang, L. Zhang, I. T. Teng, H. H. Yang, W. Tan, Self-assembly of DNA nanohydrogels with controllable size and stimuli-responsive property for targeted gene regulation therapy. *J. Am. Chem. Soc.* **137**, 1412–1415 (2015).

15. P. Kumthekar, C. H. Ko, T. Paunesku, K. Dixit, A. M. Sonabend, O. Bloch, M. Tate, M. Schwartz, L. Zuckerman, R. Lezon, R. V. Lukas, B. Jovanovic, K. McCortney, H. Colman, S. Chen, B. Lai, O. Antipova, J. Deng, L. Li, S. Tommasini-Ghelfi, L. A. Hurley, D. Unruh, N. V.

Sharma, M. Kandpal, F. M. Kouri, R. V. Davuluri, D. J. Brat, M. Muzzio, M. Glass, V. Vijayakumar, J. Heidel, F. J. Giles, A. K. Adams, C. D. James, G. E. Woloschak, C. Horbinski, A. H. Stegh, A first-in-human phase 0 clinical study of RNA interference–based spherical nucleic acids in patients with recurrent glioblastoma. *Sci. Transl. Med.* **13**, eabb3945 (2021).

16. Q. Bai, Y. Xiao, H. Hong, X. Cao, L. Zhang, R. Han, L. K. C. Lee, E. Y. Xue, X. Y. Tian, C. H. J. Choi, Scavenger receptor-targeted plaque delivery of microRNA-coated nanoparticles for alleviating atherosclerosis. *Proc. Natl. Acad. Sci. U.S.A.* **119**, e2201443119 (2022).

17. Y. Xiao, Z. Liang, M. Shyngys, A. Baekova, S. Cheung, M. B. Muljadi, Q. Bai, L. Zeng, C. H. J. Choi, In vivo interactions of nucleic acid nanostructures with cells. *Adv. Mater.* **37**, e2314232 (2024).

18. W. Sun, W. Ji, J. M. Hall, Q. Hu, C. Wang, C. L. Beisel, Z. Gu, Self-assembled DNA nanoclews for the efficient delivery of CRISPR–Cas9 for genome editing. *Angew. Chem. Int. Ed.* **54**, 12029–12033 (2015).

19. J. Liu, T. Wu, X. Lu, X. Wu, S. Liu, S. Zhao, X. Xu, B. Ding, A self-assembled platform based on branched DNA for sgRNA/Cas9/antisense delivery. *J. Am. Chem. Soc.* **141**, 19032–19037 (2019).

20. Y. Chen, R. Zhao, L. Li, Y. Zhao, Upconversion luminescence-boosted escape of DNAzyme from endosomes for enhanced gene-silencing efficacy. *Angew. Chem. Int. Ed.* **61**, e202206485 (2022).

21. C. K. K. Choi, Y. T. E. Chiu, X. Zhuo, Y. Liu, C. Y. Pak, X. Liu, Y. L. S. Tse, J. Wang, C. H. J. Choi, Dopamine-mediated assembly of citrate-capped plasmonic nanoparticles into stable core–shell nanoworms for intracellular applications. *ACS Nano* **13**, 5864–5884 (2019).

22. M. Zandieh, B. M. Hagar, J. Liu, M. Zandieh, B. M. Hagar, J. Liu, Interfacing DNA and polydopamine nanoparticles and its applications. *Part. Part. Syst. Charact.* **37**, 2000208 (2020).

23. C. K. K. Choi, J. Li, K. Wei, Y. J. Xu, L. W. C. Ho, M. Zhu, K. K. W. To, C. H. J. Choi, L. Bian, A gold@polydopamine core–shell nanoprobe for long-term intracellular detection of microRNAs in differentiating stem cells. *J. Am. Chem. Soc.* **137**, 7337–7346 (2015).

24. S. P. Narayan, C. J. Hang Choi, L. Hao, C. M. Calabrese, E. Auyeung, C. Zhang, O. J. G. M. Goor, C. A. Mirkin, S. P. Narayan, C. A. Mirkin, C. H. J. Choi, C. M. Calabrese, C. Zhang, L. Hao, E. Auyeung, C. Zhang, O. J. G. M. Goor, C. A. Mirkin, The sequence-specific cellular uptake of spherical nucleic acid nanoparticle conjugates. *Small* **11**, 4173–4182 (2015).

25. Y. Liu, C. K. K. Choi, H. Hong, Y. Xiao, M. L. Kwok, H. Liu, X. Y. Tian, C. H. J. Choi, Dopamine receptor-mediated binding and cellular uptake of polydopamine-coated nanoparticles. *ACS Nano* **15**, 13871–13890 (2021).

26. H. Yang, Z. Chen, L. Zhang, W. Y. Yung, K. C. F. Leung, H. Y. E. Chan, C. H. J. Choi, Mechanism for the cellular uptake of targeted gold nanorods of defined aspect ratios. *Small* **12**, 5178–5189 (2016).

27. T. J. Jentsch, CLC chloride channels and transporters: From genes to protein structure, pathology and physiology. *Crit. Rev. Biochem. Mol. Biol.* **43**, 3–36 (2008).

28. J. Liu, D. Zhang, Y. Li, W. Chen, Z. Ruan, L. Deng, L. Wang, H. Tian, A. Yiu, C. Fan, H. Luo, S. Liu, Y. Wang, G. Xiao, L. Chen, W. Ye, Discovery of bufadienolides as a novel class of ClC-3 chloride channel activators with antitumor activities. *J. Med. Chem.* **56**, 5734–5743 (2013).

29. S. Saha, V. Prakash, S. Halder, K. Chakraborty, Y. Krishnan, A pH-independent DNA nanodevice for quantifying chloride transport in organelles of living cells. *Nat. Nanotechnol.* **10**, 645–651 (2015).

30. G. Dai, C. K. K. Choi, Y. Zhou, Q. Bai, Y. Xiao, C. Yang, C. H. J. Choi, D. K. P. Ng, Immobilising hairpin DNA-conjugated distyryl boron dipyrromethene on gold@polydopamine core–shell nanorods for microRNA detection and microRNA-mediated photodynamic therapy. *Nanoscale* **13**, 6499–6512 (2021).

31. L. Li, T. Wan, M. Wan, B. Liu, R. Cheng, R. Zhang, The effect of the size of fluorescent dextran on its endocytic pathway. *Cell Biol. Int.* **39**, 531–539 (2015).

32. P. Paramasivam, C. Franke, M. Stöter, A. Höijer, S. Bartesaghi, A. Sabirsh, L. Lindfors, M. Yanez Arteta, A. Dahlén, A. Bak, S. Andersson, Y. Kalaidzidis, M. Bickle, M. Zerial, Endosomal escape of delivered mRNA from endosomal recycling tubules visualized at the nanoscale. *J. Cell Biol.* **221**, e202110137 (2022).

33. X. Li, T. Wang, Z. Zhao, S. A. Weinman, The ClC-3 chloride channel promotes acidification of lysosomes in CHO-K1 and Huh-7 cells. *Am. J. Physiol. Cell Physiol.* **282**, C1483–C1491 (2002).

34. M. Hara-Chikuma, B. Yang, N. D. Sonawane, S. Sasaki, S. Uchida, A. S. Verkman, ClC-3 chloride channels facilitate endosomal acidification and chloride accumulation. *J. Biol. Chem.* **280**, 1241–1247 (2005).

35. F. Lozupone, M. Borghi, F. Marzoli, T. Azzarito, P. Matarrese, E. Iessi, G. Venturi, S. Meschini, A. Canitano, R. Bona, A. Cara, S. Fais, TM9SF4 is a novel V-ATPase-interacting protein that modulates tumor pH alterations associated with drug resistance and invasiveness of colon cancer cells. *Oncogene* **34**, 5163–5174 (2015).

36. Y. Rui, D. R. Wilson, S. Y. Tzeng, H. M. Yamagata, D. Sudhakar, M. Conge, C. A. Berlinicke, D. J. Zack, A. Tuesca, J. J. Green, High-throughput and high-content bioassay enables tuning of polyester nanoparticles for cellular uptake, endosomal escape, and systemic in vivo delivery of mRNA. *Sci. Adv.* **8**, eabk2855 (2022).

37. K. V. Kilchrist, S. C. Dimobi, M. A. Jackson, B. C. Evans, T. A. Werfel, E. A. Dailing, S. K. Bedingfield, I. B. Kelly, C. L. Duvall, Gal8 visualization of endosome disruption predicts carrier-mediated biologic drug intracellular bioavailability. *ACS Nano* **13**, 1136–1152 (2019).

38. C. P. Dang, A. Leelahavanichkul, Over-expression of miR-223 induces M2 macrophage through glycolysis alteration and attenuates LPS-induced sepsis mouse model, the cell-based therapy in sepsis. *PLOS ONE* **15**, e0236038 (2020).

39. D. C. Wan, J. H. Pomerantz, L. J. Brunet, J. B. Kim, Y. F. Chou, B. M. Wu, R. Harland, H. M. Blau, M. T. Longaker, Noggin suppression enhances in vitro osteogenesis and accelerates in vivo bone formation. *J. Biol. Chem.* **282**, 26450–26459 (2007).

40. M. Zeisberg, J. I. Hanai, H. Sugimoto, T. Mammoto, D. Charytan, F. Strutz, R. Kalluri, BMP-7 counteracts TGF-β1–induced epithelial-to-mesenchymal transition and reverses chronic renal injury. *Nat. Med.* **9**, 964–968 (2003).

41. F. Tögel, J. Isaac, Z. Hu, K. Weiss, C. Westenfelder, Renal SDF-1 signals mobilization and homing of CXCR4-positive cells to the kidney after ischemic injury. *Kidney Int.* **67**, 1772–1784 (2005).

42. L. W. C. Ho, C. K. W. Chan, R. Han, Y. F. Y. Lau, H. Li, Y. P. Ho, X. Zhuang, C. H. J. Choi, Mammalian cells exocytose alkylated gold nanoparticles via extracellular vesicles. *ACS Nano* **16**, 2032–2045 (2022).

43. F. Rizvi, E. Everton, A. R. Smith, H. Liu, E. Osota, M. Beattie, Y. Tam, N. Pardi, D. Weissman, V. Gouon-Evans, Murine liver repair via transient activation of regenerative pathways in hepatocytes using lipid nanoparticle-complexed nucleoside-modified mRNA. *Nat. Commun.* **12**, 613 (2021).

44. H. D. Lawson, H. H. Nguyen, K.-J. Lee, N. Wongsuwan, A. Tupe, M. Lu, M. L. Arral, A. Behre, Z. Ling, K. A. Whitehead, A. W. Feinberg, X. Ren, S.-Y. Zheng, Synthetic strategy for mRNA encapsulation and gene delivery with nanoscale metal-organic frameworks. *Adv. Funct. Mater.* **35**, 2504465, (2025).

45. Y. Chen, X. Chen, D. Wu, H. Xin, D. Chen, D. Li, H. Pan, C. Zhou, Y. Ping, Delivery of CRISPR/Cas9 plasmids by cationic gold nanorods: Impact of the aspect ratio on genome editing and treatment of hepatic fibrosis. *Chem. Mater.* **33**, 81–91 (2021).

46. X. Jiang, S. Xu, Y. Miao, K. Huang, B. Wang, B. Ding, Z. Zhang, Z. Zhao, X. Zhang, X. Shi, M. Yu, F. Tian, Y. Gan, Curvature-mediated rapid extravasation and penetration of nanoparticles

against interstitial fluid pressure for improved drug delivery. *Proc. Natl. Acad. Sci. U.S.A.* **121**, e2319880121 (2024).

47. J. S. King, R. R. Kay, The origins and evolution of macropinocytosis. *Philos. Trans. R. Soc. Lond. B Biol. Sci.* **374**, 20180158 (2019).

48. A. Balfourier, N. Luciani, G. Wang, G. Lelong, O. Ersen, A. Khelfa, D. Alloyeau, F. Gazeau, F. Carn, Unexpected intracellular biodegradation and recrystallization of gold nanoparticles. *Proc. Natl. Acad. Sci. U.S.A.* **117**, 103–113 (2020).

49. X. Liu, J. Cao, H. Li, J. Li, Q. Jin, K. Ren, J. Ji, Mussel-inspired polydopamine: A biocompatible and ultrastable coating for nanoparticles in vivo. *ACS Nano* **7**, 9384–9395 (2013).

50. J. H. Park, G. Von Maltzahn, L. Zhang, M. P. Schwartz, E. Ruoslahti, S. N. Bhatia, M. J. Sailor, Magnetic iron oxide nanoworms for tumor targeting and imaging. *Adv. Mater.* **20**, 1630–1635 (2008).

51. J. Zhou, C. Wang, P. Wang, P. B. Messersmith, H. Duan, Multifunctional magnetic nanochains: Exploiting self-polymerization and versatile reactivity of mussel-inspired polydopamine. *Chem. Mater.* **27**, 3071–3076 (2015).

52. J. Osei-Owusu, J. Yang, K. H. Leung, Z. Ruan, W. Lü, Y. Krishnan, Z. Qiu, Proton-activated chloride channel PAC regulates endosomal acidification and transferrin receptor-mediated endocytosis. *Cell Rep.* **34**, 108683 (2021).

53. G. Novarino, S. Weinert, G. Rickheit, T. J. Jentsch, Endosomal chloride-proton exchange rather than chloride conductance is crucial for renal endocytosis. *Science* **328**, 1398–1401 (2010).

54. T. J. Jentsch, Discovery of CLC transport proteins: Cloning, structure, function and pathophysiology. *J. Physiol.* **593**, 4091–4109 (2015).

55. N. D. Sonawane, F. C. Szoka, A. S. Verkman, Chloride accumulation and swelling in endosomes enhances DNA transfer by polyamine-DNA polyplexes. *J. Biol. Chem.* **278**, 44826–44831 (2003).

56. N. G. Bastús, J. Comenge, V. Puntes, Kinetically controlled seeded growth synthesis of citrate-stabilized gold nanoparticles of up to 200 nm: Size focusing versus ostwald ripening. *Langmuir* **27**, 11098–11105 (2011).

57. G. Toda, T. Yamauchi, T. Kadowaki, K. Ueki, Preparation and culture of bone marrow-derived macrophages from mice for functional analysis. *STAR Protoc.* **2**, 100246 (2021).

58. L. W. C. Ho, W. Y. Yung, K. H. S. Sy, H. Y. Li, C. K. K. Choi, K. C. F. Leung, T. W. Y. Lee, C. H. J. Choi, Effect of alkylation on the cellular uptake of polyethylene glycol-coated gold nanoparticles. *ACS Nano* **11**, 6085–6101 (2017).

59. A. Olofsson, L. N. Skalman, I. Obi, R. Lundmark, A. Arnqvist, Uptake of helicobacter pylori vesicles is facilitated by clathrin- dependent and clathrin-independent endocytic pathways. *MBio* **5**, e00979-14 (2014).

60. S. Mahapatra, T. Takahashi, Physiological roles of endocytosis and presynaptic scaffold in vesicle replenishment at fast and slow central synapses. *eLife* **12**, RP90497 (2024).

61. H. Shen, Y. Bao, C. Feng, H. Fu, J. Mao, Overexpression of Myo1e promotes albumin endocytosis by mouse glomerular podocytes mediated by Dynamin. *PeerJ.* **8**, e8599 (2020).

62. Y. Zuo, J. Hu, X. Xu, X. Gao, Y. Wang, S. Zhu. Sodium azide induces mitochondria-mediated apoptosis in PC12 cells through Pgc-1α-associated signaling pathway. *Mol. Med. Rep.* **19**, 2211–2219 (2019).

63. M. Koivusalo, C. Welch, H. Hayashi, C. C. Scott, M. Kim, T. Alexander, N. Touret, K. M. Hahn, S. Grinstein, Amiloride inhibits macropinocytosis by lowering submembranous pH and preventing Rac1 and Cdc42 signaling. *J. Cell Biol.* **188**, 547–563 (2010).

64. M. Zhang, B. Xiao, H. Wang, M. K. Han, Z. Zhang, E. Viennois, C. Xu, D. Merlin, Edible ginger-derived nano-lipids loaded with doxorubicin as a novel drug-delivery approach for colon cancer therapy. *Mol. Ther.* **24**, 1783–1796 (2016).

65. E. S. Reynolds, The use of lead citrate at high pH as an electron-opaque stain in electron microscopy. *J. Cell Biol.* **17**, 208–212 (1963).

66. Y. Ma, R. VanKeulen-Miller, O. S. Fenton, mRNA lipid nanoparticle formulation, characterization and evaluation. *Nat. Protoc.* **20**, 2618–2651 (2025).

67. K. M. Saad, É. L. Salles, S. E. Naeini, B. Baban, M. E. Abdelmageed, R. R. Abdelaziz, G. M. Suddek, A. A. Elmarakby, Reno-protective effect of protocatechuic acid is independent of sex-related differences in murine model of UUO-induced kidney injury. *Pharmacol. Rep.* **76**, 98–111 (2024).

68. H. Wang, C. N. Alarcón, B. Liu, F. Watson, S. Searles, C. K. Lee, J. Keys, W. Pi, D. Allen, J. Lammerding, J. D. Bui, R. L. Klemke, Genetically engineered and enucleated human mesenchymal stromal cells for the targeted delivery of therapeutics to diseased tissue. *Nat. Biomed. Eng.* **6**, 882–897 (2022).

69. C. W. Dunnett, A multiple comparison procedure for comparing several treatments with a control. *J. Am. Stat. Assoc.* **50**, 1096–1121 (1955).

70. K. Du, C. D. Williams, M. R. McGill, H. Jaeschke, Lower susceptibility of female mice to acetaminophen hepatotoxicity: Role of mitochondrial glutathione, oxidant stress and c-jun N-terminal kinase. *Toxicol. Appl. Pharmacol.* **281**, 58 (2014).

71. G. Chen, Y. Wang, L. H. Tan, M. Yang, L. S. Tan, Y. Chen, H. Chen, High-purity separation of gold nanoparticle dimers and trimers. *J. Am. Chem. Soc.* **131**, 4218–4219 (2009).

72. W. Park, J. Choi, J. Hwang, S. Kim, Y. Kim, M. K. Shim, W. Park, S. Yu, S. Jung, Y. Yang, D. H. Kweon, Apolipoprotein fusion enables spontaneous functionalization of mRNA lipid nanoparticles with antibody for targeted cancer therapy. *ACS Nano* **19**, 6412–6425 (2025).

73. P. Roy, N. W. Kreofsky, M. E. Brown, C. Van Bruggen, T. M. Reineke, Enhancing pDNA delivery with hydroquinine polymers by modulating structure and composition. *JACS Au* **3**, 1876–1889 (2023).

74. J. Lee, I. Sands, W. Zhang, L. Zhou, Y. Chen, DNA-inspired nanomaterials for enhanced endosomal escape. *Proc. Natl. Acad. Sci. U.S.A.* **118**, e2104511118 (2021).

75. S. Guo, C. Li, C. Wang, X. Cao, X. Liu, X. J. Liang, Y. Huang, Y. Weng, pH-responsive polymer boosts cytosolic siRNA release for retinal neovascularization therapy. *Acta Pharm. Sin. B* **14**, 781–794 (2024).

76. X. Tang, J. Zhang, D. Sui, Q. Yang, T. Wang, Z. Xu, X. Li, X. Gao, X. Yan, X. Liu, Y. Song, Y. Deng, Simultaneous dendritic cells targeting and effective endosomal escape enhance sialic acid-modified mRNA vaccine efficacy and reduce side effects. *J. Control. Release* **364**, 529–545 (2023).

77. X. Chen, S. Wang, Y. Chen, H. Xin, S. Zhang, D. Wu, Y. Xue, M. Zha, H. Li, K. Li, Z. Gu, W. Wei, Y. Ping, Non-invasive activation of intratumoural gene editing for improved adoptive T-cell therapy in solid tumours. *Nat. Nanotechnol.* **18**, 933–944 (2023).

78. Y. Zeng, M. Shen, R. Pattipeiluhu, X. Zhou, Y. Zhang, T. Bakkum, T. H. Sharp, A. L. Boyle, A. Kros, Efficient mRNA delivery using lipid nanoparticles modified with fusogenic coiled-coil peptides. *Nanoscale* **15**, 15206–15218 (2023).

79. J. Blersch, V. Francisco, C. Rebelo, A. Jiménez-Balsa, H. Antunes, C. Gonzato, S. Pinto, S. Simões, K. Liedl, K. Haupt, L. Ferreira, A light-triggerable nanoparticle library for the controlled release of non-coding RNAs. *Angew. Chem. Int. Ed. Engl.* **132**, 2001–2007 (2020).

80. M. Chen, J. Cen, Q. Shi, B. Shao, J. Tan, X. Ye, Z. He, Y. Liu, G. Zhang, J. Hu, J. Bao, S. Liu, Ultrasound-enhanced spleen-targeted mRNA delivery via fluorinated pegylated lipid nanoparticles for immunotherapy. *Angew. Chem. Int. Ed. Engl.* **64**, e202500878 (2025).

81. C. Li, J. Zhou, Y. Wu, Y. Dong, L. Du, T. Yang, Y. Wang, S. Guo, M. Zhang, A. Hussain, H. Xiao, Y. Weng, Y. Huang, X. Wang, Z. Liang, H. Cao, Y. Zhao, X. J. Liang, A. Dong, Y. Huang, Core role of hydrophobic core of polymeric nanomicelle in endosomal escape of siRNA. *Nano Lett.* **21**, 3680–3689 (2021).

82. P. B. Tiwade, Y. Ma, R. VanKeulen-Miller, O. S. Fenton, A lung-expressing mRNA delivery platform with tunable activity in hypoxic environments. *J. Am. Chem. Soc.* **146**, 17365–17376 (2024).

83. I. F. de la Fuente, S. S. Sawant, K. W. Kho, N. K. Sarangi, R. C. Canete, S. Pal, L. H. Liang, T. E. Keyes, J. L. Rouge, Determining the role of surfactant on the cytosolic delivery of DNA cross-linked micelles. *ACS Appl. Mater. Interfaces* **16**, 43400–43415 (2024).

84. Y. Chen, R. Li, Q. Duan, L. Wu, X. Li, A. Luo, Y. Zhang, N. Zhao, K. Cui, W. Wu, T. Liu, J. B. Wan, L. Deng, G. Li, L. Hou, W. Tan, Z. Xiao, A DNA-modularized STING agonist with macrophage-selectivity and programmability for enhanced anti-tumor immunotherapy. *Adv. Sci.* **11**, 2400149 (2024).

85. W. Zai, W. Chen, Z. Wu, X. Jin, J. Fan, X. Zhang, J. Luan, S. Tang, X. Mei, Q. Hao, H. Liu, D. Ju, Targeted interleukin-22 gene delivery in the liver by polymetformin and penetratin-based hybrid nanoparticles to treat nonalcoholic fatty liver disease. *ACS Appl. Mater. Interfaces* **11**, 4842–4857 (2019).

86. S. Liu, J. Liu, H. Li, K. Mao, H. Wang, X. Meng, J. Wang, C. Wu, H. Chen, X. Wang, X. Cong, Y. Hou, Y. Wang, M. Wang, Y. G. Yang, T. Sun, An optimized ionizable cationic lipid for brain tumor-targeted siRNA delivery and glioblastoma immunotherapy. *Biomaterials* **287**, 121645 (2022).

87. T. T. Pham, H. Chen, P. H. D. Nguyen, M. K. Jayasinghe, A. H. Le, M. T. Le, Endosomal escape of nucleic acids from extracellular vesicles mediates functional therapeutic delivery. *Pharmacol. Res.* **188,** 106665 (2023).

88. W. Wee, M. Soh, E. Finol, S. J. W. Chan, J.-Y. Zhu, S. Sean, J. Kang Liau, A. Bier, E. Ooi, G. C. Bazan, W. W. M. Soh, S. J. W. Chan, J.-Y. Zhu, G. C. Bazan, S. S. J. K. Liau, A. Bier, E. Finol, E. E. Ooi, Tailoring lipid nanoparticle with ex situ incorporated conjugated oligoelectrolyte for enhanced mRNA delivery efficiency. *Adv. Healthc. Mater.* **14**, e2405048 (2025).

89. H. Xian, Y. Song, J. Qu, Y. Shi, Y. Zhang, W. Wu, M. Kim, Y. Wang, C. Yu, CaClOH-modified silica nanoparticles for mRNA delivery. *Nano Lett.* **25**, 6365–6373 (2025).

90. J. Xu, J. Xu, C. Sun, X. He, Y. Shu, Q. Huangfu, L. Meng, Z. Liang, J. Wei, M. Cai, J. Wen, B. Wang, Effective delivery of CRISPR/dCas9-SAM for multiplex gene activation based on mesoporous silica nanoparticles for bladder cancer therapy. *Acta Biomater.* **197**, 460–475 (2025).

91. A. P. Pontes, S. van der Wal, S. R. Ranamalla, K. Roelofs, I. Tomuta, L. B. Creemers, J. Rip, Cell uptake and intracellular trafficking of bioreducible poly(amidoamine) nanoparticles for efficient mRNA translation in chondrocytes. *Front. Bioeng. Biotechnol.* **11**, 1290871 (2023).

92. Y. J. Sung, H. Guo, A. Ghasemizadeh, X. Shen, W. Chintrakulchai, M. Kobayashi, M. Toyoda, K. Ogi, J. Michinishi, T. Ohtake, M. Matsui, Y. Honda, T. Nomoto, H. Takemoto, Y. Miura, N. Nishiyama, Cancerous pH-responsive polycarboxybetaine-coated lipid nanoparticle for smart delivery of siRNA against subcutaneous tumor model in mice. *Cancer Sci.* **113**, 4339–4349 (2022).

93. Y. Zhao, J. Qin, D. Yu, Y. Liu, D. Song, K. Tian, H. Chen, Q. Ye, X. Wang, T. Xu, H. Xuan, N. Sun, W. Ma, J. Zhong, P. Sun, Y. Song, J. Hu, Y. Zhao, X. Hou, X. Meng, C. Jiang, J. Cai, Polymer-locking fusogenic liposomes for glioblastoma-targeted siRNA delivery and CRISPR–Cas gene editing. *Nat. Nanotechnol.* **19**, 1869–1879 (2024).

94. Z. Ma, S. W. Wong, H. Forgham, L. Esser, M. Lai, M. N. Leiske, K. Kempe, G. Sharbeen, J. Youkhana, F. Mansfeld, J. F. Quinn, P. A. Phillips, T. P. Davis, M. Kavallaris, J. A. McCarroll, Aerosol delivery of star polymer-siRNA nanoparticles as a therapeutic strategy to inhibit lung tumor growth. *Biomaterials* **285**, 121539 (2022).

95. T. Cristofolini, M. Dalmina, J. A. Sierra, A. H. Silva, A. A. Pasa, F. Pittella, T. B. Creczynski-Pasa, Multifunctional hybrid nanoparticles as magnetic delivery systems for siRNA targeting the HER2 gene in breast cancer cells. *Mater. Sci. Eng. C* **109**, 110555 (2020).

96. J. Zhao, A. Tong, J. Liu, M. Xu, P. Mi, Tumor-targeting nanocarriers amplified immunotherapy of cold tumors by STING activation and inhibiting immune evasion. *Sci. Adv.* **11**, eadr1728 (2025).

97. R. Dabas, N. Navaratnam, H. Iino, S. Saidjalolov, S. Matile, D. Carling, D. S. Rueda, N. Kamaly, Precise intracellular uptake and endosomal release of diverse functional mRNA payloads via glutathione-responsive nanogels. *Mater. Today Bio.* **30**, 101425 (2025).

98. R. Oude Egberink, D. M. van Schie, B. Joosten, L. T. A. de Muynck, W. Jacobs, J. van Oostrum, R. Brock, Unraveling mRNA delivery bottlenecks of ineffective delivery vectors by co-transfection with effective carriers. *Eur. J. Pharm. Biopharm.* **202**, 114414 (2024).

99. T. Hao, B. Zhang, W. Li, X. Yang, S. Wu, Y. Yuan, H. Cui, Q. Chen, Z. Li, Nordihydroguaiaretic acid-cross-linked phenylboronic acid-functionalized polyplex micelles for anti-angiogenic gene therapy of orthotopic and metastatic tumors. *ACS Appl. Mater. Interfaces* **16**, 34620–34631 (2024).

100. H. Wang, Y. Yuan, L. Qin, M. Yue, J. Xue, Z. Cui, X. Zhan, J. Gai, X. Zhang, J. Guan, S. Mao, Tunable rigidity of PLGA shell-lipid core nanoparticles for enhanced pulmonary siRNA delivery in 2D and 3D lung cancer cell models. *J. Control. Release* **366**, 746–760 (2024).

101. Y. Wang, Y. Xie, K. V Kilchrist, J. Li, C. L. Duvall, D. Oupický, Endosomolytic and tumor-penetrating mesoporous silica nanoparticles for siRNA/miRNA combination cancer therapy. *ACS Appl. Mater. Interfaces* **12**, 4308–4322 (2020).

102. X. Feng, B. Liu, Z. Zhou, W. Li, J. Zhao, L. Li, Y. Zhao, Engineering hierarchical metal-organic@metal-DNA heterostructures for combinational tumor treatment. *Nano Res* **16**, 12633–12640 (2023).

103. Y. Ma, O. S. Fenton, Tannic acid lipid nanoparticles can deliver messenger RNA payloads and improve their endosomal escape. *Adv. Ther.* **6**, 2200305 (2023).

104. S. K. Bhangu, L. Mummolo, S. Fernandes, A. Amodio, A. Radziwon, B. Dyett, M. Savioli, N. Mantri, C. Cortez-Jugo, F. Caruso, F. Cavalieri, Tracking the endosomal escape of nanoparticles in live cells using a triplex-forming oligonucleotide. *Adv. Funct. Mater.* **34**, 2311240 (2024)

105. S. Narum, B. Deal, H. Ogasawara, J. N. Mancuso, J. Zhang, K. Salaita, An endosomal escape trojan horse platform to improve cytosolic delivery of nucleic acids. *ACS Nano* **18**, 6186–6201 (2024).

106. B. Li, M. Zhao, W. Lai, X. Zhang, B. Yang, X. Chen, Q. Ni, Activatable NIR-II photothermal lipid nanoparticles for improved messenger RNA delivery. *Angew. Chem. Int. Ed. Engl.* **62**, e202302676 (2023).

107. Y. Mo, M. H. Y. Cheng, A. D'Elia, K. Doran, L. Ding, J. Chen, P. R. Cullis, G. Zheng, Light-activated siRNA endosomal release (LASER) by porphyrin lipid nanoparticles. *ACS Nano* **17**, 4688–4703 (2023).

108. S. Zhao, K. Gao, H. Han, M. Stenzel, B. Yin, H. Song, A. Lawanprasert, J. E. Nielsen, R. Sharma, O. H. Arogundade, S. Pimcharoen, Y. J. Chen, A. Paul, J. Tuma, M. G. Collins, Y. Wyle, M. G. Cranick, B. W. Burgstone, B. S. Perez, A. E. Barron, A. M. Smith, H. Y. Lee, A. Wang, N. Murthy, Acid-degradable lipid nanoparticles enhance the delivery of mRNA. *Nat. Nanotechnol.* **19**, 1702–1711 (2024).

109. Y. Cao, J. Long, H. Sun, Y. Miao, Y. Sang, H. Lu, C. Yu, Z. Zhang, L. Wang, J. Yang, S. Wang, Dendritic cell-mimicking nanoparticles promote mRNA delivery to lymphoid organs. *Adv. Sci.* **10**, 2302423 (2023).

110. P. Guzmán-Sastoque, S. Sotelo, N. P. Esmeral, S. L. Albarracín, J. J. Sutachan, L. H. Reyes, C. Muñoz-Camargo, J. C. Cruz, N. I. Bloch, Assessment of CRISPRa-mediated GDNF overexpression in an in vitro Parkinson's disease model. *Front. Bioeng. Biotechnol.* **12**, 1420183 (2024).

111. J. Chen, A. Patel, M. Mir, M. R. Hudock, M. R. Pinezich, B. Guenthart, M. Bacchetta, G. Vunjak-Novakovic, J. Kim, Enhancing cytoplasmic expression of exogenous mRNA through dynamic mechanical stimulation. *Adv. Healthc. Mater.* **14**, 2401918 (2024).

112. H. Liu, M. Z. Chen, T. Payne, C. J. H. Porter, C. W. Pouton, A. P. R. Johnston, Beyond the endosomal bottleneck: Understanding the efficiency of mRNA/LNP delivery. *Adv. Funct. Mater.* **34**, 2404510 (2024).

113. Y. Ma, P. B. Tiwade, R. VanKeulen-Miller, E. A. Narasipura, O. S. Fenton, Polyphenolic nanoparticle platforms (PARCELs) for in vitro and in vivo mRNA delivery. *Nano Lett.* **24**, 6092–6101 (2024).

114. C. Pegoraro, E. Masiá Sanchis, S. Đorđević, I. Dolz-Pérez, C. Huck-Iriart, L. Herrera, S. Esteban-Pérez, I. Conejos-Sanchez, M. J. Vicent, Multifunctional polypeptide-based nanoconjugates for targeted mitochondrial delivery and nonviral gene therapy. *Chem. Mater.* **37**, 1457–1467 (2025).

115. Y. Shi, H. Zhang, H. Chen, J. Guo, R. Yuan, Y. Tian, Q. Xin, Z. Mu, Y. Tao, Y. Chu, A. Wang, Z. Zhang, J. Tian, H. Wang, Cetuximab-immunoliposomes loaded with TGF-β1 siRNA for the targeting therapy of NSCLC: Design, and in vitro and in vivo evaluation. *Int. J. Mol. Sci.* **26**, 1196 (2025).

116. L. Yin, Z. Fu, M. Wang, B. Liu, X. Sun, K. Liu, X. Feng, Z. He, Y. Wang, J. Hou, X. Shao, N. Yang, T. Zhang, Y. Liu, Z. Huang, Q. Yin, Y. Xie, Y. Li, T. Lang, A prodrug nanodevice co-delivering docetaxel and ROR1 siRNA for enhanced triple negative breast cancer therapy. *Acta Biomater.* **193**, 498–513 (2025).

117. Y. Jiang, R. Jiang, Z. Xia, M. Guo, Y. Fu, X. Wang, J. Xie, Engineered neutrophil membrane-camouflaged nanocomplexes for targeted siRNA delivery against myocardial ischemia reperfusion injury. *J. Nanobiotechnology* **23**, 134 (2025).

118. Y. Wang, M. Wang, J. Yang, M. Liang, H. Liu, L. Wang, A. Peng, Y. Xiang, R. Huang, Y. Dong, D. Gong, H. Xie, J. Zhao, J. Liu, C. Yi, Q. Liu, E. Bian, D. Tian, Cation-free siRNA

micelle knockdown of EPHA2 improves sensitivity of imatinib for osteosarcoma treatment. *J. Chem. Eng.* **513**, 162705 (2025).

119. S. Narum, J. Zhang, B. L. N. Vo, J. N. Mancuso, K. Salaita, Exploring the subcellular localization and degradation of spherical nucleic acids using fluorescence lifetime imaging microscopy. *ACS Nano* **19**, 21983–21996 (2025).

120. D. Li, Q. Wu, C. Long, P. Yi, S. Wang, Q. Wang, W. Teng, Hybrid-designed metal-phenolic nanoparticles for synergistic nano-gene periodontal therapy. *Biomaterials* **322**, 123417 (2025).

121. S. Weiss, S. Decker, C. Kugler, L. B. Gómez, H. Fasching, D. Benisch, F. Alioglu, L. Ferencz, T. Birkfeld, F. Ilievski, V. Baumann, A. Duran, E. Dusinovic, N. Follrich, S. Milenkovic, D. Mihalicokova, D. Paunov, K. Singeorzan, N. Zehetmayer, D. Zivanonvic, U. Lächelt, A. Boersma, T. Rülicke, H. Sami, M. Ogris, Accelerated endosomal escape of splice-switching oligonucleotides enables efficient hepatic splice correction. *ACS Appl. Mater. Interfaces* **17**, 9000–9018 (2025).

122. C. He, P. He, Y. Ou, X. Tang, H. Wei, Y. Xu, S. Bai, Z. Guo, R. Hu, K. Xiong, G. Du, X. Sun, Rectifying the crosstalk between the skeletal and immune systems improves osteoporosis treatment by core-shell nanocapsules. *ACS Nano* **19**, 5549–5567 (2025).

123. Q. F. Zhang, R. M. Zhao, Y. Lei, X. L. Tian, Y. Hu, L. Zhang, J. Zhang, Cinnamaldehyde-based ROS-responsive polymeric gene vectors for efficient gene delivery and tumor cell growth inhibition. *Biomacromolecules* **26**, 1362–1371 (2025).

124. M. S. Padilla, K. Mrksich, Y. Wang, R. M. Haley, J. J. Li, E. L. Han, R. El-Mayta, E. H. Kim, S. Dias, N. Gong, S. V. Teerdhala, X. Han, V. Chowdhary, L. Xue, Z. Siddiqui, H. M. Yamagata, D. Kim, I. C. Yoon, J. M. Wilson, R. Radhakrishnan, M. J. Mitchell, Branched endosomal disruptor (BEND) lipids mediate delivery of mRNA and CRISPR-Cas9 ribonucleoprotein complex for hepatic gene editing and T cell engineering. *Nat. Commun.* **16**, 996 (2025).

125. J. Lv, Q. Fan, Y. Zhang, X. Zhou, P. Yu, X. Yu, C. Xin, J. Hong, Y. Cheng, A serum resistant polymer with exceptional endosomal escape and mRNA delivery efficacy for CRISPR gene therapy. *Adv. Sci.* **12**, 2413006 (2025).

126. C. Zhao, C. Wang, W. Shan, W. Wang, H. Deng, Fusogenic lipid nanovesicle for biomacromolecular delivery. *Nano Lett.* **24**, 8609–8618 (2024).

127. Y. Zhuo, Z. Luo, Z. Zhu, J. Wang, X. Li, Z. Zhang, C. Guo, B. Wang, D. Nie, Y. Gan, G. Hu, M. Yu, Direct cytosolic delivery of siRNA via cell membrane fusion using cholesterol-enriched exosomes. *Nat. Nanotechnol.* **19**, 1858–1868 (2024).

128. J. Ma, Y. Zhu, J. Kong, D. Yu, W. H. Toh, M. Jain, Q. Ni, Z. Ge, J. Lin, J. Choy, L. Cheng, K. Konstantopoulos, M. F. Konig, S. X. Sun, H.-Q. Mao, Tuning extracellular fluid viscosity to enhance transfection efficiency. *Nat. Chem. Eng.* **1**, 576–587 (2024).

129. H. Lee, G. You, S. Yeo, H. Lee, H. Mok, Effects of histidine oligomers in lipid nanoparticles on siRNA delivery. *Macromol. Biosci.* **24**, 2400043 (2024).

130. M. S. Alqahtani, R. Syed, A. S. Alqahtani, O. M. Almarfadi, M. A. Roni, S. S. Sadhu, Synthesis and bioactivity of a novel surfactin-based lipopeptide for mRNA delivery. *Nanoscale Adv.* **6**, 5193–5206 (2024).

131. T. D. Luong, N. Martel, J. Rae, H. P. Lo, Y.-W. Lim, Y. Wu, K.-A. McMahon, N. Fletcher, K. Thurecht, A. P. R. Johnston, N. Ariotti, T. E. Hall, R. G. Parton, A modular encapsulation system for precision delivery of proteins, nucleic acids and therapeutics. bioRxiv 607124 [Preprint] (2024); https://doi.org/10.1101/2024.08.22.607124.

132. C. R. Palmer, L. E. Pastora, B. R. Kimmel, H. M. Pagendarm, A. J. Kwiatkowski, P. T. Stone, K. Arora, N. Francini, O. Fedorova, A. M. Pyle, J. T. Wilson, C. R. Palmer, L. E. Pastora, B. R. Kimmel, A. J. Kwiatkowski, P. T. Stone, K. Arora, J. T. Wilson, H. M. Pagendarm, N. Francini, Covalent polymer-RNA conjugates for potent activation of the RIG-I pathway. *Adv. Healthc. Mater.* **14**, e2303815 (2024).

133. H. Zhang, C. Meng, X. Yi, J. Han, J. Wang, F. Liu, Q. Ling, H. Li, Z. Gu, Fluorinated lipid nanoparticles for enhancing mRNA delivery efficiency. *ACS Nano* **18**, 7825–7836 (2024).

134. K. Xu, Y. Xu, J. Sun, X. Cheng, C. Lu, W. Chen, B. He, T. Jiang, Piperazine-derived ionizable lipids for enhanced mRNA delivery and cancer immunotherapy. *Nano Res* **17**, 7357–7364 (2024).

135. X. Chen, H. Chen, L. Zhu, M. Zeng, T. Wang, C. Su, G. Vulugundam, P. Gokulnath, G. Li, X. Wang, J. Yao, J. Li, D. Cretoiu, Z. Chen, Y. Bei, Nanoparticle-patch system for localized, effective, and sustained miRNA administration into infarcted myocardium to alleviate myocardial ischemia-reperfusion injury. *ACS Nano* **18**, 19470–19488 (2024).

136. X. Han, M. G. Alameh, N. Gong, L. Xue, M. Ghattas, G. Bojja, J. Xu, G. Zhao, C. C. Warzecha, M. S. Padilla, R. El-Mayta, G. Dwivedi, Y. Xu, A. E. Vaughan, J. M. Wilson, D. Weissman, M. J. Mitchell, Fast and facile synthesis of amidine-incorporated degradable lipids for versatile mRNA delivery in vivo. *Nat. Chem.* **16**, 1687–1697 (2024).

137. P. Schneider, H. Zhang, L. Simic, Z. Dai, B. Schrörs, Ö. Akilli-Öztürk, J. Lin, F. Durak, J. Schunke, V. Bolduan, B. Bogaert, D. Schwiertz, G. Schäfer, M. Bros, S. Grabbe, J. M. Schattenberg, K. Raemdonck, K. Koynov, M. Diken, L. Kaps, M. Barz, Multicompartment polyion complex micelles based on triblock polypept(o)ides mediate efficient siRNA delivery to cancer-associated fibroblasts for antistromal therapy of hepatocellular carcinoma. *Adv. Mater.* **36**, 2404784 (2024).

138. F. Zhang, T. Burghardt, M. Höhn, E. Wagner, Dual effect by chemical electron transfer enhanced siRNA lipid nanoparticles: Reactive oxygen species-triggered tumor cell killing aggravated by Nrf2 gene silencing. *Pharmaceutics* **16**, 779 (2024).

139. J. M. Johansson, H. Du Rietz, H. Hedlund, H. C. Eriksson, E. Oude Blenke, A. Pote, S. Harun, P. Nordenfelt, L. Lindfors, A. Wittrup, Cellular and biophysical barriers to lipid nanoparticle mediated delivery of RNA to the cytosol. bioRxiv 596627 [Preprint] (2024); https://doi.org/10.1101/2024.05.31.596627.

140. X. Bian, L. Yang, D. Jiang, A. J. Grippin, Y. Ma, S. Wu, L. Wu, X. Wang, Z. Tang, K. Tang, W. Pan, S. Dong, B. Y. S. Kim, W. Jiang, Z. Yang, C. Li, Regulation of cerebral blood flow boosts precise brain targeting of vinpocetine-derived ionizable-lipidoid nanoparticles. *Nat. Commun.***15**, 3987 (2024).

141. M. Yazdi, J. Pöhmerer, M. Hasanzadeh Kafshgari, J. Seidl, M. Grau, M. Höhn, V. Vetter, C. C. Hoch, B. Wollenberg, G. Multhoff, A. Bashiri Dezfouli, E. Wagner, In vivo endothelial cell gene silencing by siRNA-LNPs tuned with lipoamino bundle chemical and ligand targeting. *Small* **20**, 2400643 (2024).

142. Z. Wang, J. Zhang, Y. Wang, J. Zhou, X. Jiao, M. Han, X. Zhang, H. Hu, R. Su, Y. Zhang, W. Qi, Overcoming endosomal escape barriers in gene drug delivery using de novo designed pH-responsive peptides. *ACS Nano* **18**, 10324–10340 (2024).

143. I. G. Kim, W. H. Jung, G. You, H. Lee, Y. J. Shin, S. W. Lim, B. H. Chung, H. Mok, Efficient delivery of globotriaosylceramide synthase siRNA using polyhistidine-incorporated lipid nanoparticles. *Macromol. Biosci.* **23**, 2200423 (2023).

144. Q. Bao, X. Liu, Y. Li, T. Yang, H. Yue, M. Yang, C. Mao, Development of magnetic nanoparticles with double silica shells of different porosities for efficient siRNA delivery to breast cancer cells. *Mater. Adv.* **5**, 1626–1630 (2024).

145. H. S. Kim, Y. R. Ahn, M. Kim, J. Choi, S. J. Shin, H. O. Kim, Charge-complementary polymersomes for enhanced mRNA delivery. *Pharmaceutics* **15**, 2781 (2023).

146. Y. Wan, Y. Yang, Q. Lai, W. Wang, M. Wu, S. Feng, Fluorinated cell-penetrating peptide for co-delivering siHIF-1α and sorafenib to enhance in vitro anti-tumor efficacy. *Pharmaceutics* **15**, 2789 (2023).

147. H. Hedlund, H. Du Rietz, J. M. Johansson, H. C. Eriksson, W. Zedan, L. Huang, J. Wallin, A. Wittrup, Single-cell quantification and dose-response of cytosolic siRNA delivery. *Nat. Commun.* **14**, 1075 (2023).

148. M. Gao, M. Tang, W. Ho, Y. Teng, Q. Chen, L. Bu, X. Xu, X. Q. Zhang, Modulating plaque inflammation via targeted mRNA nanoparticles for the treatment of atherosclerosis. *ACS Nano* **17**, 17721–17739 (2023).

149. A. Chakraborty, S. Dharmaraj, N. Truong, R. M. Pearson, Excipient-free ionizable polyester nanoparticles for lung-selective and innate immune cell plasmid DNA and mRNA transfection. *ACS Appl. Mater. Interfaces* **14**, 56440–56453 (2022).

150. H. Zhang, F. Ding, Z. Zhu, Q. Sun, C. Yang, Engineered ionizable lipid nanoparticles mediated efficient siRNA delivery to macrophages for anti-inflammatory treatment of acute liver injury. *Int. J. Pharm.* **631**, 122489 (2023).

151. N. Kong, R. Zhang, G. Wu, X. Sui, J. Wang, N. Y. Kim, S. Blake, D. De, T. Xie, Y. Cao, W. Tao, Intravesical delivery of KDM6A-mRNA via mucoadhesive nanoparticles inhibits the metastasis of bladder cancer. *Proc. Natl. Acad. Sci. U.S.A.* **119**, e2112696119 (2022).

152. A. F. Rodrigues, C. Rebelo, S. Simões, C. Paulo, S. Pinho, V. Francisco, L. Ferreira, A polymeric nanoparticle formulation for targeted mRNA delivery to fibroblasts. *Adv. Sci.* **10**, 2205475 (2023).

153. W. Cai, T. Luo, X. Chen, L. Mao, M. Wang, A combinatorial library of biodegradable lipid nanoparticles preferentially deliver mRNA into tumor cells to block mutant RAS signaling. *Adv. Funct. Mater.* **32**, 2204947 (2022).

154. J. Duan, C. Bao, Y. Xie, H. Guo, Y. Liu, J. Li, R. Liu, P. Li, J. Bai, Y. Yan, L. Mu, X. Li, G. Wang, W. Lu, Targeted core-shell nanoparticles for precise CTCF gene insert in treatment of metastatic breast cancer. *Bioact. Mater.* **11**, 1–14 (2022).

155. X. Zhao, Y. Wang, W. Jiang, Q. Wang, J. Li, Z. Wen, A. Li, K. Zhang, Z. Zhang, J. Shi, J. Liu, Herpesvirus-mimicking DNAzyme-loaded nanoparticles as a mitochondrial DNA stress inducer to activate innate immunity for tumor therapy. *Adv. Mater.* **34**, 2204585 (2022).

156. Y. Wang, P. K. Shahi, X. Wang, R. Xie, Y. Zhao, M. Wu, S. Roge, B. R. Pattnaik, S. Gong, In vivo targeted delivery of nucleic acids and CRISPR genome editors enabled by GSH-responsive silica nanoparticles. *J. Control. Release* **336**, 296–309 (2021).

157. P. Zhang, X. Li, Q. Xu, Y. Wang, J. Ji, Polydopamine nanoparticles with different sizes for NIR-promoted gene delivery and synergistic photothermal therapy. *Colloids Surf. B Biointerfaces* **208**, 112125 (2021).

158. P. Norouzi, H. Motasadizadeh, F. Atyabi, R. Dinarvand, M. Gholami, M. Farokhi, M. A. Shokrgozar, F. Mottaghitalab, Combination therapy of breast cancer by codelivery of doxorubicin and survivin siRNA using polyethylenimine modified silk fibroin nanoparticles. *ACS Biomater Sci. Eng.* **7**, 1074–1087 (2021).

159. J. H. Park, A. Mohapatra, J. Zhou, M. Holay, N. Krishnan, W. Gao, R. H. Fang, L. Zhang, Virus-mimicking cell membrane-coated nanoparticles for cytosolic delivery of mRNA. *Angew. Chem. Int. Ed. Engl.* **61**, e202113671 (2022).

160. S. Lu, X. Bao, W. Hai, S. Shi, Y. Chen, Q. Yu, M. Zhang, Y. Xu, J. Peng, Multi-functional self-assembled nanoparticles for pVEGF-shRNA loading and anti-tumor targeted therapy. *Int. J. Pharm.* **575**, 118898 (2020).

161. Y. Wang, P. K. Shahi, R. Xie, H. Zhang, A. A. Abdeen, N. Yodsanit, Z. Ma, K. Saha, B. R. Pattnaik, S. Gong, A pH-responsive silica–metal–organic framework hybrid nanoparticle for the delivery of hydrophilic drugs, nucleic acids, and CRISPR-Cas9 genome-editing machineries. *J. Control. Release* **324**, 194–203 (2020).

162. H. Tanaka, T. Takahashi, M. Konishi, N. Takata, M. Gomi, D. Shirane, R. Miyama, S. Hagiwara, Y. Yamasaki, Y. Sakurai, K. Ueda, K. Higashi, K. Moribe, E. Shinsho, R. Nishida, K. Fukuzawa, E. Yonemochi, K. Okuwaki, Y. Mochizuki, Y. Nakai, K. Tange, H. Yoshioka, S. Tamagawa, H. Akita, Self-degradable lipid-like materials based on “hydrolysis accelerated by the intra-particle enrichment of reactant (HyPER)” for messenger RNA delivery. *Adv. Funct. Mater.* **30**, 1910575 (2020).

163. W. Tao, A. Yurdagul Jr, N. Kong, W. Li, X. Wang, A. C. Doran, C. Feng, J. Wang, M. Ariful Islam, O. C. Farokhzad, I. Tabas, J. Shi, siRNA nanoparticles targeting CaMKIIγ in lesional macrophages improve atherosclerotic plaque stability in mice. *Sci. Transl. Med.* **12**, eaay1063 (2020).

164. J. Zhuang, H. Gong, J. Zhou, Q. Zhang, W. Gao, R. H. Fang, L. Zhang, Targeted gene silencing in vivo by platelet membrane-coated metal-organic framework nanoparticles. *Sci. Adv.* **6**, eaaz6108 (2020).

165. X. Hou, X. Zhang, W. Zhao, C. Zeng, B. Deng, D. W. McComb, S. Du, C. Zhang, W. Li, Y. Dong, Vitamin lipid nanoparticles enable adoptive macrophage transfer for the treatment of multidrug-resistant bacterial sepsis. *Nat. Nanotechnol.* **15**, 41–46 (2020).

166. X. Zhang, W. Zhao, G. N. Nguyen, C. Zhang, C. Zeng, J. Yan, S. Du, X. Hou, W. Li, J. Jiang, B. Deng, D. W. McComb, R. Dorkin, A. Shah, L. Barrera, F. Gregoire, M. Singh, D. Chen, D. E. Sabatino, Y. Dong, Functionalized lipid-like nanoparticles for in vivo mRNA delivery and base editing. *Sci. Adv.* **6**, eabc2315 (2020).

167. P. Zhang, Q. Xu, X. Li, Y. Wang, pH-responsive polydopamine nanoparticles for photothermally promoted gene delivery. *Mater. Sci. Eng. C* **108**, 110396 (2020).

168. I. B. Kelly, R. B. Fletcher, J. R. McBride, S. M. Weiss, C. L. Duvall, Tuning composition of polymer and porous silicon composite nanoparticles for early endosome escape of anti-microRNA peptide nucleic acids. *ACS Appl. Mater. Interfaces* **12**, 39602–39611 (2020).

169. G. Li, Y. Gao, C. Gong, Z. Han, L. Qiang, Z. Tai, J. Tian, S. Gao, Dual-blockade immune checkpoint for breast cancer treatment based on a tumor-penetrating peptide assembling nanoparticle. *ACS Appl. Mater. Interfaces* **11**, 39513–39524 (2019).

170. S. K. Brar, M. Verma, Measurement of nanoparticles by light-scattering techniques. *TrAC Trends Anal. Chem.* **30**, 4–17 (2011).

171. J. Rodríguez-Fernández, J. Pérez-Juste, L. M. Liz-Marzán, P. R. Lang, Dynamic light scattering of short au rods with low aspect ratios. *J. Phys. Chem. C* **111**, 5020–5025 (2007).

172. A. Ortega, J. García de la Torre, Hydrodynamic properties of rodlike and disklike particles in dilute solution. *J. Chem. Phys.* **119**, 9914–9919 (2003).
